# Contrasting evolutionary patterns of helper and sensor NRC NLRs in lettuce reflect functional divergence following subfunctionalization

**DOI:** 10.1371/journal.pgen.1012245

**Published:** 2026-07-16

**Authors:** Hsuan Pai, Toshiyuki Sakai, Andres Posbeyikian, Raoul Frijters, Yu Sugihara, Mauricio P. Contreras, Jiorgos Kourelis, Hiroaki Adachi, Sophien Kamoun, AmirAli Toghani

**Affiliations:** 1 The Sainsbury Laboratory, University of East Anglia, Norwich Research Park, Norwich, United Kingdom; 2 Laboratory of Crop Evolution, Graduate School of Agriculture, Kyoto University, Kyoto, Japan; 3 Rijk Zwaan Breeding B.V., Department of Biotechnology, Fijnaart, The Netherlands; 4 Department of Life Sciences, Imperial College, London, United Kingdom; 5 JST-PRESTO, Saitama, Japan; Université Paris-Saclay: Universite Paris-Saclay, FRANCE

## Abstract

Nucleotide-binding domain and leucine-rich repeat immune receptors (NLRs) are known for their rapid evolution, even at the intraspecific level, yet the rates of evolution differ significantly across NLRs. However, the degree to which evolutionary patterns reflect functional divergence remains poorly understood, notably in important crop species. Within the NRC (NLR Required for Cell Death) network in Asterids, sensor NLRs detect pathogen presence but require NRC helpers for signaling and to confer immunity. We conducted a comparative analysis of NLRs across 40 Solanales and 29 Asterales genomes to explore NRC network expansion and diversification within the less-studied Asterales order. Our findings reveal that the NRC network has expanded less in Asterales compared to Solanales. We functionally validated an Asterales NRC network with 2 helpers and 9 sensors in common lettuce (*Lactuca sativa*). Through selection analysis and structural modeling of NRC gene family in the *Lactuca* genus, we found distinct evolutionary trajectories between NRC helpers and sensors. Sensors reliant on the phylogenetically conserved helper NRC0 experience limited diversification, whereas sensors dependent on other NRC helpers show higher rates of positive selection and gene duplication. Our results highlight the lineage- and function-specific evolution of the NRC network, offering insights into the evolutionary pressures shaping plant immune receptor networks.

## Author summary

Plant immune systems rely on intracellular receptors that detect pathogen molecules and activate defense responses. In many flowering plants, these receptors operate within signaling networks where “sensor” receptors detect pathogens and depend on “helper” receptors to trigger immunity. We studied one such network, called the NRC network, present across many major asterid crops like tomato, potato, sunflower, and lettuce. The NRC network has expanded to vastly different extents across asterid plant lineages, with some species carrying hundreds of sensors and multiple helpers while others retain only a handful. Despite this variation, we found that the underlying evolutionary patterns within the NRC network are reflected across taxonomic lineages. The evolutionary tempo of sensors is associated with functional dependency within the network: sensors coupled to conserved helpers evolve slowly, whereas sensors dependent on more recently emerged helpers diversify rapidly. Our findings reveal how functional relationships within immune receptor networks impose distinct evolutionary trajectories on their constituent members.

## Introduction

Plants possess a sophisticated innate immune system to defend against a wide array of pathogens [1,2]. A group of intracellular immune receptors, known as nucleotide-binding domain and leucine-rich repeat-containing receptors (NLRs), are essential components of this defense system. These receptors detect pathogen-secreted effector proteins. Upon recognition, NLRs trigger a cascade of defense responses, often culminating in a localized programmed cell death that effectively restricts pathogen spread [3,4]. NLR proteins represent one of the most diverse protein families, with different NLR genes evolving at varying rates [5–7]. However, the relationship between the rate of NLR evolution and their functional roles remains poorly understood.

NLR proteins are typically composed of three main domains: an N-terminal domain, often a coiled-coil (CC) or Toll-interleukin receptor (TIR), which is responsible for signal transduction; a central NB-ARC (nucleotide-binding domain shared with APAF-1, plant R proteins, and CED-4) domain which plays a role in regulating activation and signaling; and a C-terminal leucine-rich repeat (LRR) domain, which directly or indirectly facilitates pathogen detection [2,8,9]. Based on their NB-ARC phylogeny, NLRs are classified into four classes: CC-NLRs (CNLs), CC_G10_-type CC-NLRs, RPW8-type CC-NLRs (CC_R_-NLRs/RNLs), and TIR-NLRs (TNLs) [9,10]. While the tripartite architecture is largely conserved, some NLRs contain additional or non-canonical domains, such as integrated domains (IDs), which contribute to pathogen detection [11–14]. About 20% of CC-NLRs harbor a conserved N-terminal MADA motif essential for triggering cell death [15]. Activated CC-NLRs often form higher-order complexes called “resistosome” [16–20], which likely insert into the plasma membrane to act as calcium channels via their N-terminal alpha-helix [18,21,22]. Recent advances in computational structural biology have allowed for modeling NLR resistosomes in the presence of simulated plasma membrane with high confidence [19,23].

While some NLRs function as independent units, many form functionally dependent pairs or networks of specialized NLRs [4,24]. NLR pairs, often clustered genetically, work together, such as Pik-1/Pik-2 and RGA5/RGA4 CC-NLR pairs in rice (*Oryza sativa*) and the RPS4/RRS1 TIR-NLR pair in *Arabidopsis thaliana* [11,14,25–29]. In NLR networks, sensor NLRs depend on helper NLRs for immunity, following one-to-many or many-to-one configurations [4,24,30,31]. For instance, the CC_R_-NLRs NRG1 and ADR1 function downstream of the TIR-NLR signaling pathway [32–35]. The NRC network, first identified in Solanaceae plants like tomato and *Nicotiana benthamiana*, also exemplifies a CC-NLR network, where sensor NLRs rely on NRC helpers (NRC-H) to activate immune responses [24,30]. NRC-dependent sensors (NRC-S) are divided into two subtypes based on domain architecture: Rx-type sensors have a canonical CC-NB-ARC-LRR structure, while SD-type sensors include an additional N-terminal Solanaceous Domain (SD) that facilitates pathogen recognition [4,36–38]. Upon pathogen effector detection, NRC sensors transmit a signal that triggers the assembly of helper NRCs from a homodimer resting state into hexameric resistosomes that induce immune responses and cell death [19,20,39–42]. Though the exact mechanism of signal transmission between sensor and helper NRCs remains unclear, it likely involves transient “activation-and-release” interactions [38–40].

**Fig 1:**
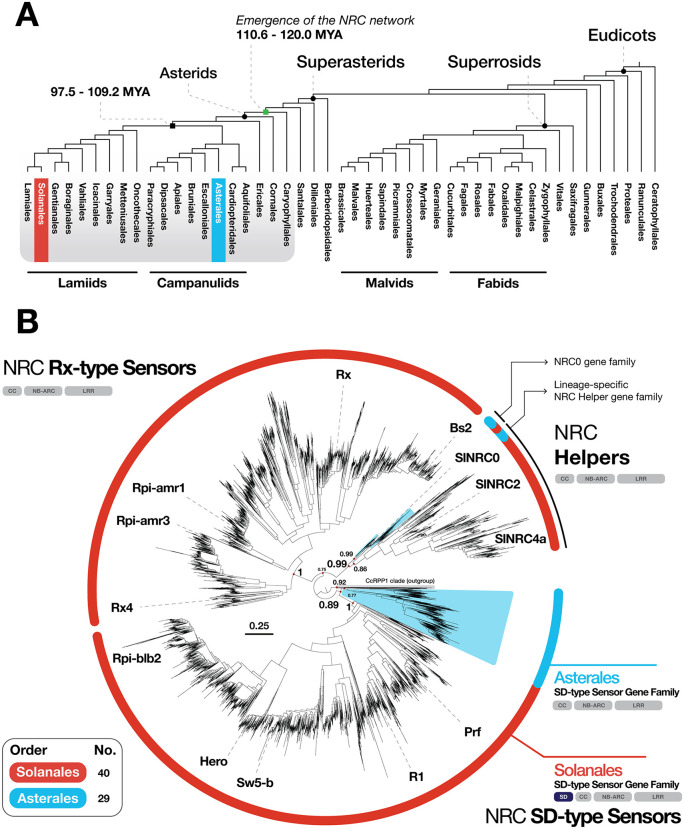
The NRC network has expanded less in Asterales compared to Solanales. **(A)** Taxonomic tree of eudicots highlighting the timeline of NRC network emergence within Asterids [30,43]. Phylogenetic relationships and divergence time estimates were obtained from TimeTree 5 [74]. MYA: Million Years Ago; NRC: NLR Required for Cell-death. **(B)** Phylogenetic tree of the NB-ARC domains from 7,040 sequences belonging to the NRC network, with CcRPP1 sequences used as outgroup, derived from 40 Solanales and 29 Asterales genomes and constructed using FastTree 2 [75]. Asterales branches are highlighted in blue. Numbers on tree nodes indicate bootstrap support values. NRC helpers (NRC-H) and sensors (NRC-S) form distinct, well-supported phylogenetic groups. NRC-H are divided into two major phylogroups: NRC0 and a lineage-specific group expanded in Asterales and Solanales. NRC-S are further divided into SD and Rx type phylogroups, with the Rx-types notably absent in Asterales. SD: Solanaceous Domain.

The NRC network, a gene family consisting of NRC-H and NRC-S (hereafter referred to as NRC network), likely originated ~100 million years ago within the superasterid lineage of land plants and has expanded massively in the lamiid lineage [30,43,44] (Fig 1A). While particularly prominent in Solanaceae, its expansion and complexity vary across plant families [43]. NRC0, the most conserved and ancient NRC helper in asterids, represents the ancestral state of the NRC network and is frequently genetically linked to its dependent NRC-S [43,44]. In contrast, other NRCs have undergone lineage-specific expansions and exhibit diverse genomic arrangements, with some NRC-H physically clustered alongside NRC-S, such as NRC6, and others dispersed throughout the genome [36,43,45]. While sensor NLRs tend to evolve rapidly in response to pathogen evolution, helper NLRs are typically more conserved [43]. However, recent findings show that paralogous helper NLRs experience diversification pressures that maintain homodimerization while preventing heterodimerization, thereby minimizing cross-activation and promoting the isolation of distinct signaling pathways. [42].

NLR numbers vary widely across species, from a few to over a thousand in some species [43,46–49], reflecting the dynamic co-evolutionary arms race between plants and pathogens [50]. NLR gene evolution follows the birth-and-death model, where gene duplication generates new NLRs—some are maintained via selection for their ability to detect pathogens, while others lose function or are deleted [37,50–52]. Recent advances have introduced new evolutionary models, emphasizing the dynamic diversification of NLRs under pathogen pressure [25,50,52,53]. Pathogen effectors often target NLRs to suppress immunity, but NLRs counteract this through mutations in critical regions, while the presence of functional redundancy allows the system to remain robust [24,53]. For instance, within the NRC network, helpers like NRC2 and NRC3 are suppressed by cyst nematode effector SS15 and oomycete effector AVRcap1b [54,55]. However, in some species NRC3 has accumulated mutations in its effector-binding region that enable it to evade suppression [53]. Additionally, signaling redundancy allows non-suppressed nodes, such as NRC4, to activate immune responses even when parts of the pathway are suppressed [24,53,55,56].

Asterales is one of the most diverse flowering plant orders within the Asterid clade, encompassing several commercially important crops, including the common lettuce (*Lactuca sativa*) [57,58]. Lettuce has become a key model for studying genetic resistance to pathogens and NLR evolution [59–68]. Meyers et al. (1998) categorized the lettuce NLRome into 42 phylogenetic groups, known as Resistance Gene Clusters (RGCs) [65,66]. These NLR genes were further grouped into eight Major Resistance Clusters (MRCs) based on their locations on chromosomes 1, 2, 3, 4, 8, and 9 [66,67]. In another study, Kuang et al. (2004) reported that genes at the RGC2 locus in lettuce could be categorized into two types: type I (diverse) and type II (conserved), with contrasting patterns of evolution and copy number between them [69]. This concept has since been explored in other systems and NLR families, including *Arabidopsis thaliana* and *Zea mays* (maize) [70–72]. Despite extensive functional and genetic research on resistance in lettuce, the evolution of the NRC network in Asterales remain poorly understood.

The NRC network underpins resistance to a wide range of pathogens in Solanaceous crops, where its sensor/helper architecture, copy-number variation, and genomic organization are increasingly well characterized. Whether these features are conserved in other agronomically important lineages, and how they relate to functional divergence between helpers and sensors, remains poorly understood. The Asterales include major crops such as lettuce (*Lactuca sativa*), yet the extent of NRC network expansion, the genomic clustering of NRC genes, and the selective pressures acting on helpers versus sensor subgroups in this lineage warrant further investigation. In this study, we set out to (i) characterise the expansion and genomic organisation of the NRC network across 29 Asterales genomes, compared to 40 Solanales genomes; (ii) test whether a functional NRC network operates in common lettuce; and (iii) determine whether helpers and the different sensor subgroups show distinct evolutionary signatures within the *Lactuca* genus. We found that the NRC network in Asterales shows limited expansion compared to Solanales, with NLRs in the Rx-type sensor phylogenetic group absent in Asterales. We confirmed the presence of a functional NRC network in common lettuce, and through selection analyses of NRC-H and NRC-S within the Lactuca genus, we observed distinct patterns of diversification, not only between helpers and sensors but also among different sensor phylogroups. NRC-S dependent on NRC0 show limited diversification and expansion, whereas sensors relying on other NRC helpers have undergone greater expansion and higher rates of diversification. Moreover, we found that in contrast to NRC-H, NRC-S are not predicted by AlphaFold 3 to assemble into resistosome-like structures. Together, these findings provide insights into the evolution of the NRC network and the relationships among NLRs evolving at different rates.

## Results

### The NRC network shows limited gene expansion in Asterales compared to Solanales

The presence of the NRC network across Asterids prompted us to investigate the extent of its gene copy number in Asterales, a Campanulid order, compared to the well-studied Solanales order from Lamiids (Fig 1A) [30]. For this purpose, we extracted a total number of 21,232 non-redundant NLR sequences from a dataset of de-novo annotated 40 Solanales and 29 Asterales genomes with harmonized annotations using NLRtracker, representing a diverse set of species and genera from the two orders (S1 and S2 Data) [9,73]. Based on phylogenetics analysis, we then extracted the monophyletic NRC network comprising 7,020 sequences, including the reference NRC-H and NRC-S from RefPlantNLR (S1 Fig and S3 Data) [9]. In Asterales and Solanales, there are on average 300 and 338.6 non-redundant NLRs per genome, respectively. However, NRC-type NLRs on average only comprise 6.6% of the NLRome in Asterales genomes compared to 50.3% in Solanales (S2 Fig).

Compared to Solanales, Asterales show much less expansion of NRC-S and NRC-H genes (Figs 1B and S2). To further understand the evolutionary patterns of diversification in Asterales we performed phylogenetic analysis on the 592 NRC-H and NRC-S NLRs, 88 and 504, respectively. We divided the 88 Asterales NRC-H into two phylogroups. One group, containing 27 sequences phylogenetically grouped with NRC0, while the remaining 61 sequences formed a separate phylogroup and grouped with known Solanales NRC helpers, such as NRC2 and NRC4, referred to as Ast-NRCs hereafter (Fig 1B) [30]. All 504 Asterales NRC-S formed a well-supported phylogroup next to the Solanales SD-type sensors, but none were grouped with the Rx-type Solanales sensors (Fig 1B). Although Asterales NRC-S belong to the SD-type class, they lack the Solanaceous domain characteristic at their N-termini (Fig 1B and S3 Data) [37]. These observations suggest notable differences in the evolutionary pace and expansion of the NRC network between Solanales and Asterales orders.

In common lettuce, the 17 NRC sequences (comprising 2 helpers and 15 sensors) represent only 5% of the total NLRome, which consists of 359 sequences. In contrast, TIR-NLRs dominate the NLRome with 206 sequences, making up over 57%. The CC-NLR, CC_R_-NLR, and CC_G10_-NLR classes contain 97, 9, and 42 sequences, respectively (S3 Fig). This indicates a disproportionate expansion of different NLR classes, with a limited expansion of NRC-H and NRC-S in lettuce.

### Asterales NRC networks exhibit lineage-specific expansion of NRC-H and NRC-S

**Fig 2:**
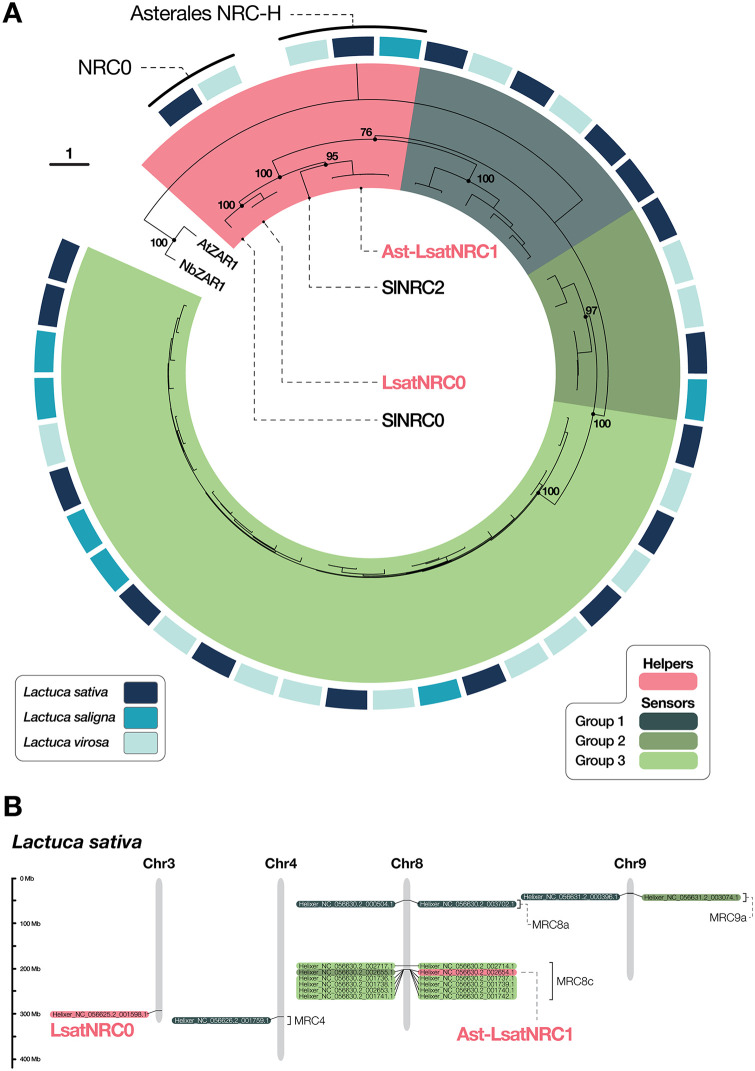
Unlike LsatNRC0, Ast-LsatNRC1 is genetically linked to NRC-S in lettuce. **(A)** Phylogenetic tree of NB-ARC domains from 40 NRC sequences of three *Lactuca* species (*Lactuca sativa*, *Lactuca saligna*, and *Lactuca virosa*) constructed using IQ-TREE 2 [76]. Lactuca NRC gene family is divided into an NRC-H and three well-supported NRC-S phylogenetic orthogropus. Numbers on tree nodes indicate bootstrap support values. AtZAR1 and NbZAR1 were used as outgroup sequences. SlNRC0 and SlNRC2 were used as reference sequences. At: *Arabidopsis thaliana*; Nb: *Nicotiana benthamiana*; Sl: *Solanum lycopersicum*; Ast: Asterales. **(B)** Physical map of *Lactuca sativa* NRC sequences on the chromosomes. While Ast-LsatNRC1 is genetically linked to group 3 NRC-S, LsatNRC0 is located on Chromosome 3, not linked to any sensor NLRs. Previously described Major Resistance Clusters (MRCs) are shown for NRCs [66,67]. Lsat: *Lactuca sativa.*

Given the observed differences in the expansion of NRC network between Solanales and Asterales, we sought to investigate the Asterales NRC network gene copy number and genomic structure in more detail, focusing on lettuce. We analyzed 40 NRC-H and NRC-S sequences from common lettuce (*Lactuca sativa*) and two wild lettuce species (*Lactuca saligna* and *Lactuca virosa*) (S4 Data). Through phylogenetic analysis, we divided the *Lactuca* NRC sequences into four phylogroups: one helper phylogroup containing sequences that grouped with either NRC0 or tomato NRC2 (referred to as Ast-NRC1 in each species hereafter), and sensor phylogroups 1, 2, and 3 (referred to as sensor group 1,2, and 3 hereafters.) (Fig 2A). These phylogenetic groups were defined by well-supported branches (Figs 2A and S5). Based on the previous categorization of NLR families in lettuce, NRC sequences are distributed across four RGC groups: (1) the NRC helper group as RGC7, (2) sensor group 1 as RGC26, (3) sensor group 2 as RGC27, and (4) sensor group 3 as RGC9 (S3 Fig) [65,66].

NRC-H and NRC-S are often found in genetic clusters like NRC0 and the Solanaceae NRC6 [36,44]. Therefore, we examined the physical location of NRC sequences in the lettuce genomes. Unlike previous observations in various Asterid species, lettuce NRC0 (LsatNRC0; Helixer_NC_056625.2_001598.1), on chromosome 3, was not located near any sensor sequences (Fig 2B) [44]. In contrast, Ast-LsatNRC1 (Helixer_NC_056630.2_002654.1) was found to be in a physical cluster (<100kb) with sensors from groups 2 and 3, all residing on chromosome 8. Notably, all of group 3 sensors are clustered together on this chromosome. This genomic cluster is part of the previously reported MRC8c (Fig 2B) [64–66]. The remaining lettuce NRC-S sequences, including those from group 1, are scattered across chromosomes 4, 8, and 9, as part of MRCs 4, 8a, and 9a, respectively (Fig 2B) [64–66]. Only LsatNRC0 is not part of any of the reported MRCs (Fig 2B).

Due to a lack of chromosome-level assemblies for the wild lettuce species, we could not determine if the NRC-H and NRC-S in these genomes are genetically clustered together. However, our genome analysis revealed Ast-LvirNRC1 (Helixer_CAKMRJ010003334.1_001958.1) positioned near group 2 and 3 sensors on contig2395 (Figs 2A and S4). We further examined five other species across Asterales to investigate whether NRC-S are in genetic clusters with the NRC helpers as a general trend (S5 Fig). We classified the NRC sequences in these species based on their phylogenetic relationship to the *Lactuca* NRC-S and NRC-H. In *Cichorium intybus*, CiNRC0 (Helixer_CM042016.1_001204.1) is located within 500K base pair distance of a group 1 NRC sensor on chromosome 8 (Helixer_CM042016.1_001201.1), whereas Ast-CiNRC1a (Helixer_CM042010.1_003195.1) and Ast-CiNRC1b (Helixer_CM042010.1_003196.1) are clustered with sensors from group 2 and 3 on chromosome 2 (S6 Fig and S6 Data). In *Cynara cardunculus*, CcNRC0 (Helixer_NC_037542.1_000084.1) is in close proximity with a group 1 sensor (Helixer_NC_037542.1_001617.1) on chromosome 15, and Ast-CcNRC1 is in a genetic cluster with four group 1 sensors on contig NW_020200580.1 (S7 Fig and S6 Data). In *Helianthus annuus*, *Chrysanthemum lavandulifolium* and *Codonopsis lanceolata*, none of the NRC-H are clustered with any NRC-S (S6 and S7 Figs and S6 Data). These findings suggest variations in the physical clustering of NRC-H and NRC-S within the *Lactuca* genus and between different Asterales species.

### LsatNRC0 and Ast-LsatNRC1 are required for cell death signaling of distinct NRC-S phylogenetic groups

To investigate the functional connections of the lettuce NRC network and to test the phylogeny-based classification of these NLRs into NRC-S and NRC-H, we used Agrobacterium-mediated transient co-expression assays (Agroinfiltration) in an *nrc2/3/4* CRISPR knockout line of *Nicotiana benthamiana* [30]. We agroinfiltrated autoactive mutants of the putative NRC-S and NRC-H to test their functionality, as the cognate effectors for these sensors remain unidentified. These mutants were generated by introducing a D-to-V mutation within the MHD motif of the NLRs, located near the C-terminus of the NB-ARC domain [15]. We first tested whether transiently expressed NRC-S or NRC-H alone could induce cell death in *N. benthamiana*. While none of the NRC-S triggered cell death on their own, the autoactive mutants of the two lettuce NRC-H displayed a macroscopic hypersensitive cell death response (HR) (S8A Fig).

**Fig 3:**
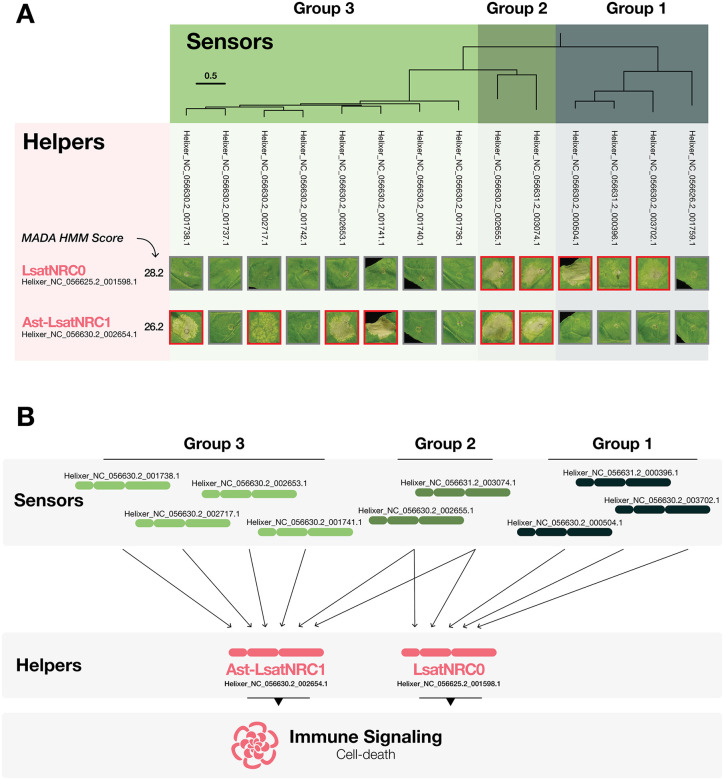
LsatNRC0 and Ast-LsatNRC1 mediate the cell death signaling of distinct NRC-S phylogenetic groups. **(A)** Co-agroinfiltration cell death assays of NRC-H and NRC-S. 14 NRC-S expressed as autoactive mutants (D-to-V mutation in the MHD motif) with two wild-type NRC-H. Sensor-helper pairs agroinfiltrated together causing cell death are outlined with red squares. While group 1 and group 3 sensors can only signal through LsatNRC0 and Ast-LsatNRC1, respectively, group 2 sensors can signal through both. Sensor-helper pairs with no visible cell-death were not assessed in terms of protein accumulation. Lsat: *Lactuca sativa;* Ast: Asterales. **(B)** Lettuce NRC network model. The 9 validated NRC-S can cause cell death through LsatNRC0 and Ast-LsatNRC1 in a partially redundant manner.

We then co-expressed the NRC-S and NRC-H in pairs to assess their potential functional connections and the dependence of lettuce NRC-S on the two NRC helpers. Among the 16 tested NRC-S, 9 displayed visible HR as autoactive mutants when co-expressed with one or both wild-type NRCs. While the cell death phenotype of functional sensors was consistent across two replicates, protein accumulation was not assessed for sensor–helper pairs that did not trigger cell death. Three NRC-S induced cell death through only LsatNRC0, while four responded solely through Ast-LsatNRC1. Notably, two sensors were functionally connected with both LsatNRC0 and Ast-LsatNRC1 (Figs 3 and S8B and S7 Data). Specifically, all LsatNRC0-dependent sensors belonged to group 1, while Ast-LsatNRC1-dependent sensors resided in group 3. NRC-S that functioned with both helpers are group 2 members (Fig 3A). Furthermore, we assessed if lettuce NRC-H and NRC-S possessed the MADA motif required for cell-death activity of previously characterized NRC-H. While both NRC helpers possessed MADA motifs, none of the 15 tested sensors got a hit for MADA HMM profile (Fig 3 and S8 Data) [15]. This was consistent with the cell death assays showing NRC helpers but not NRC sensors are able to cause cell death on their own [15,77].

Although we showed functional connection between NRC-H and NRC-S in common lettuce, this remains to be experimentally determined in other species including those from *Lactuca virosa*, *Lactuca saligna*, and other Asterales species. However, based on phylogenetic relationships and absence of both NRC0 and group 1 sensors, we assumed the NRC network in *Lactuca* species follow a similar specificity profile following phylogenetic grouping. Taken together these observations indicate the presence of a functional NLR network with partially redundant signaling pathways in lettuce (Fig 3B).

### In contrast to NRC helpers, NRC sensors fail to form resistosome-like oligomers when modeled with AlphaFold 3

**Fig 4:**
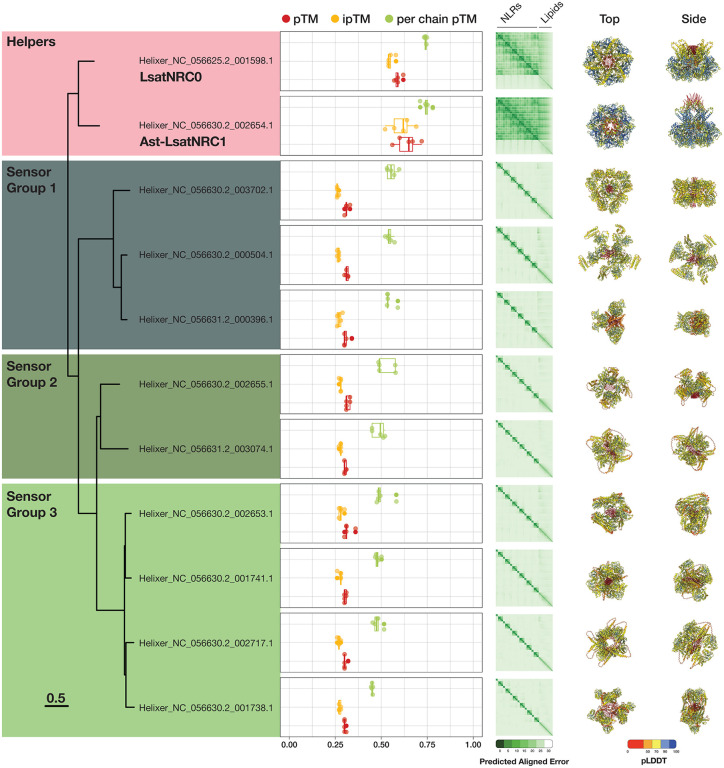
In contrast to NRC-H, NRC-S failed to form resistosome-like structures when modeled with AlphaFold 3. Structural modeling with AlphaFold 3 was used to predict the ability of the tested lettuce NRC-H and NRC-S to form resistosome-like hexameric oligomers. Modeling included 50 oleic acid molecules to approximate the plasma membrane. Key structural metrics, including pLDDT (per-residue measure of local confidence), pTM (predicted template modeling score for overall structure), ipTM (interface-specific pTM), and per-chain pTM, are plotted for each protein (n = 5). Example predicted structures from the fourth replicate are displayed in top and side views, colored with the pLDDT color scale indicating local confidence levels. Lsat: *Lactuca sativa;* Ast: Asterales.

Recently, we demonstrated that AlphaFold 3 [78] can differentiate between helper and sensor NLRs within pairs by predicting resistosome-like structures for helpers but not for sensors [23]. Building on these findings, we applied a similar approach to the lettuce NRC network to investigate potential differences in the *in-silico* oligomerization of NRC-H and NRC-S. Using AlphaFold 3, we modeled five replicates of the two NRC-H and 16 NRC-S sequences from lettuce as hexamers in the presence of 50 oleic acid molecules, simulating the plasma membrane environment. To evaluate these models, we extracted and analyzed key metrics, including pLDDT (per-residue measure of local confidence), pTM (predicted template modeling score for overall structure), ipTM (interface-specific pTM), and per-chain pTM (S8 Data). AlphaFold 3 successfully modeled resistosome-like oligomers for both NRC-H sequences, though it was unable to generate a complete N-terminal funnel in any of the LsatNRC0 predicted structures (Fig 4). This was consistent with our previous observations when modeling NRC0 oligomers [19]. In contrast, none of the NRC-S from the three sensor groups formed resistosome-like structures (Fig 4). Across all replicates, NRC-S models consistently showed low confidence with high predicted aligned errors (Figs 4 and S9). Structurally, NRC-S displayed lower metrics compared to NRC helpers, with pTM and ipTM values below 0.5 and per-chain pTM values below 0.7 (Figs 4 and S9). Conversely, helpers had consistently higher metrics, with pTM and ipTM exceeding 0.5 in all replicates and an average per-chain pTM of 0.75 (Figs 4 and S9). These results suggest that AlphaFold 3 can be used to distinguish between NRC-S and NRC-H and their capacity to form higher-order resistosome-like oligomers.

### NRC-S dependent on Ast-LsatNRC1 are under stronger positive selection compared to LsatNRC0-dependent sensors

To examine the selection pressures that affected mutations in the NRC helper and NRC-S phylogenetic groups, we conducted positive selection tests on *Lactuca* NRC sequences using CodeML in PAML v4.10.7 software [79]. These tests compare evolutionary models that allow a proportion of sites to evolve under positive selection (ω = dN/dS > 1) against null models that do not. A significantly better fit of the positive selection model indicates that diversifying selection has driven amino acid changes at specific positions. Individual sites under positive selection are then identified using Bayes Empirical Bayes (BEB) analysis [80]. For each NRC group, we compared the M8 model allowing positive selection against its null counterpart, M8a, using likelihood ratio tests (see Methods; S10 Data).

**Fig 5:**
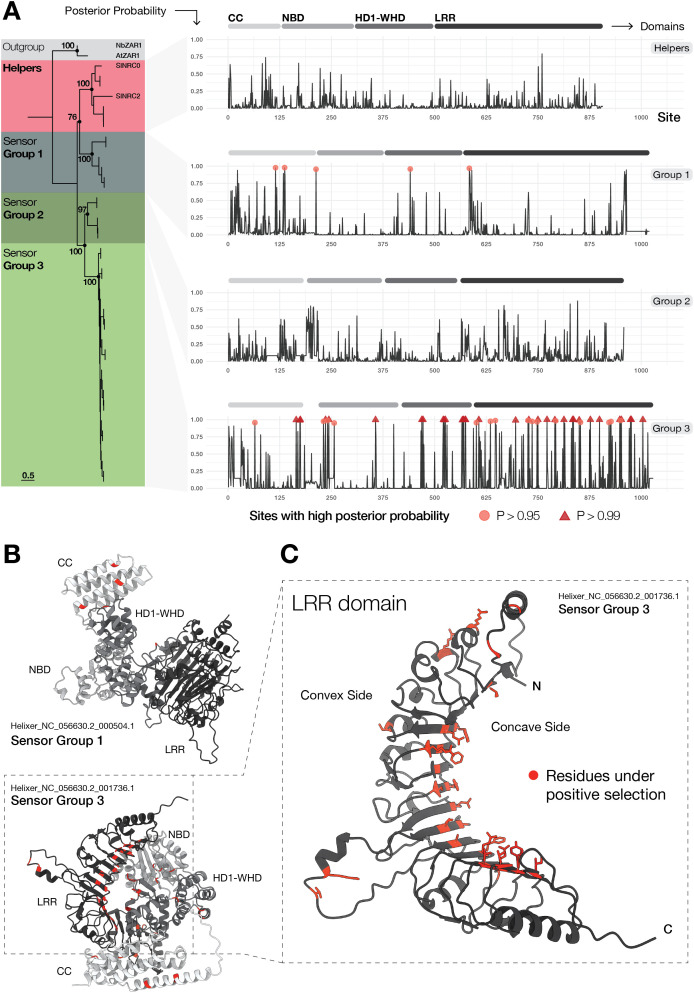
Ast-LsatNRC1-dependent sensors are under more positive selection compared to NRC0-dependent sensors and NRC helpers. **(A)** The y-axis represents the posterior probability that each site belongs to the positively selected site class (ω > 1), as estimated by the Bayes Empirical Bayes (BEB) method under the M8 model (beta + *ω*) from the CodeML package in PAML [79]. Sites with posterior probability (P) > 0.95 and > 0.99 are indicated by circles and triangles, respectively. No sites under positive selection were detected in NRC-H or group 2 sensors. In contrast, group 1 and 3 NRC-S showed several sites under positive selection, with most in group 3 localized to the LRR domain. **(B)** Sites under positive selection (red) mapped onto predicted structures of representative NRC-S from groups 1 and 3, generated using AlphaFold 3. **(C)** Zoomed-in view of the LRR domain from a representative group 3 NRC-S (Helixer_NC_056630.2_001736.1), highlighting sites under positive selection (red). Most sites under positive selection are located at the concave side of the LRR domain. CC: Coiled-Coil; NBD: Nucleotide-binding domain; HD1: Helix domain 1; WHD: Winged-helix domain; LRR: Leucine-rich repeat.

For NRC-H, the M8a vs M8 comparison did not reach our 1% significance threshold (p = 0.037), and BEB analysis did not identify any sites at either posterior probability threshold. Similarly, although the M8a vs M8 comparison was significant for sensor group 2 (p = 0.003), BEB analysis did not identify any sites at either posterior probability threshold (S10 Data). A significant LRT unaccompanied by BEB-supported sites indicates that the improved fit of M8 is not underpinned by identifiable positively selected codons and is most parsimoniously interpreted as a false positive of the M8a vs M8 test, which is known to be sensitive to alignment noise and gene conversion. In contrast, both sensor groups 1 and 3 showed evidence of positive selection (S10 Data). BEB analysis under the M8 model identified five sites under positive selection in group 1 (posterior probability > 0.95), three of which localized to the CC domain when mapped onto an AlphaFold 3-predicted structure (Helixer_NC_056630.2_000504.1; Fig 5B). Sensor group 3 showed the strongest signatures, with 48 sites under positive selection (posterior probability > 0.95), 33 of which exceeded 0.99. The majority of these sites mapped to the LRR domain (Helixer_NC_056630.2_001736.1), localized to its concave face, a region implicated in effector binding [51,70] (Fig 5C).

To formally test whether NRC subclades differed in average selective pressure, we used PAML’s branch model 2 (model = 2, NSsites = 0), assigning each phylogroup (helpers, sensor groups 1, 2, and 3) its own ω parameter and comparing it to the one-ratio model (M0) by likelihood ratio test. The four-ratio model fit significantly better than M0 (2·ΔlnL = 305.66, df = 4, P = 6.5 × 10^−65^), with ω = 0.12 for the helpers, 0.35 and 0.39 for sensor groups 1 and 2, and 0.58 for sensor group 3 (S10 Fig and S10 Data). The helper clade therefore shows the lowest average ω, group 3 the highest, and groups 1 and 2 intermediate values, consistent with the M8 BEB results placing positive selection most prominently in group 3. Although the four subclades differ in sequence number and tree depth, their total tree dS lengths from the M0 fit are broadly comparable (1.51–2.87; S10 Data), with group 3’s larger size (24 sequences) offset by its shallower per-branch divergence (≈0.06 substitutions/branch versus 0.17–0.25 in the other subclades). To visualise branch-level heterogeneity, the free-ratio model (M1) is shown as S11 Fig; per-branch ω estimates are presented descriptively rather than as a formal test, and branches with ω ≥ 10 are flagged in the figure caption as artefacts of low synonymous substitution rates rather than meaningful estimates.

Next, we estimated the pairwise nonsynonymous (*d*_N_) and synonymous (*d*_S_) substitution rates and *d*_N_/*d*_S_ ratios (*ω*) for members of each NRC-H and NRC-S group using the Nei and Gojobori (1986) method (S12 Fig and S11 Data) [81,82]. Analyses were conducted on full-length sequences and individual domains (CC, NB-ARC, and LRR), where *ω* > 1 implied presence of positive selection. Among full-length and domain-based comparisons, NRC-H had lower average ω compared to all NRC-S groups (S13 Fig). While sensor group 3 on average had overall higher ω than the rest of NRC-H and NRC-S, the CC domain in group 1 sensors had *ω* > 1 values, indicative of positive selection (S13 Fig). Overall, the pairwise *d*_N_/*d*_S_ ratios of NRC-H and NRC-S phylogroups and selection analysis pointed to NRC-S dependent on Ast-LsatNRC1 being under stronger positive selection compared to NRC-S dependent on LsatNRC0.

## Discussion

The NRC network gene family originated approximately 100 million years ago within the superasterid lineage of land plants [30]. In this study, we conducted a comparative phylogenetic and diversification analysis of the NRC network in the Asterales and Solanales orders of the Asterid clade. For this purpose, we used a *de novo* generated annotation dataset for 40 Solanales and 29 Asterales genomes using the deep-learning-based Helixer software, ensuring comparable and harmonized gene models across all analyzed genomes [83,84]. We then focused on common lettuce as a model Asterales species and experimentally validated a functional and partially redundant NRC network that was identified using phylogenetics analysis. We used this model NRC network to study the diversification and evolution of different NRC-H and NRC-S gene families.

The NRC network has expanded in the lamiid lineage of Asterids over tens of millions of years, whereas in the campanuliid lineage, including Asterales, it has experienced much less expansion [43]. Our analysis of 40 Solanales and 29 Asterales genomes revealed marked differences in NRC network evolution between these orders. Notably, NRC type NLRs make up only 6.6% of the total NLR repertoire in Asterales, compared to 50.3% in Solanales. Despite similar average total NLR counts (338.6 in Solanales vs. 300 in Asterales), Asterales appear to have expanded other NLR classes, such as TIR-NLRs. In lettuce, for example, TIR-NLRs comprise a majority of the NLRome (57.4%; 206/359), while NRC sequences represent only 5% (18/359). This aligns with previous findings of extensive proliferation of the NRC network in lamiids versus campanuliids [43]. Our study found no evidence of Rx-type sensors in Asterales, a class significantly expanded in Solanales and linked to many characterized R-genes in Solanaceous species [30]. Moreover, although Asterales SD-type sensors phylogenetically cluster with their Solanales counterparts, they lack the N-terminal SD-domain. It was previously suggested that this is due to a loss of the sensors with SD integration in Asterales [37]. However, the presence of SD-type-like sensors in sugar beet (*Beta vulgaris*; Caryophyllales) suggests that sensors with and without the SD integration were already present in the common ancestor of superasterids [37], but the sensors harboring the SD integration were possibly lost in the ancestral Asterales species. More in-depth phylogenetic analysis across superasterids may provide insights into the origin of SD-type sensors and possible SD integration/emergence events.

In lettuce, NRCs are divided into four distinct phylogenetic groups: an NRC-H and three NRC-S groups (C1, C2, and C3). Mapping these phylogenetic groups to the classification by Meyers et al. (1998) revealed their alignment with four gene clusters (RGC7, RGC9, RGC26, and RGC27) [65]. Notably, Ast-LsatNRC1 (RGC7), all group 3 sensors (RGC9), and a single group 2 sensor (RGC27) are part of Major Resistance Cluster 8c, which has been linked with QTLs for resistance to *Fusarium oxysporum* and *Verticillium dahliae* [64]. Other NRC-S from groups 1 and 2 are associated with MRCs 4, 8a, and 9a, while *NRC0* is not linked to any reported MRC. Although MRC4 and 9a have been implicated in downy mildew resistance, to date, NRC type NLRs have not yet been implicated in resistance to pathogens in common lettuce [64,65]. However, further investigation into the role of NRC network components classified as MRC8c in resistance against *F. oxysporum* and *V. dahliae* would be valuable, as they represent 12 out of 26 immune receptor genes in this genomic cluster [64].

*NRC0* is the most conserved NRC helper across superasterids [43,44], while other NRC helpers, such as NRC2, NRC3, and NRC4 in Solanaceae, show lineage-specific expansions [42,43]. Although Asterales have a less extensive overall expansion of NRCs compared to Solanales, many species in Asterales retain an additional NRC helper besides *NRC0*. Previous studies have demonstrated that NRC helpers, including *NRC0* and *NRC6*, can be genetically clustered with NRC sensors in several plant species [36,44]. However, in common lettuce, while Ast-LsatNRC1 is clustered with group 2 and 3 sensors, *LsatNRC0* is not physically clustered with any sensor NLRs. Further examination across Asterid species revealed species-specific patterns in the genetic linkage of NRC genes. For instance, in *Cichorium intybus* (chicory), *CiNRC0* is proximal to a group 1 sensor, and both *Ast-CiNRC1* copies cluster with group 2 and 3 sensors. However, in *Helianthus annuus* (sunflower), no helpers are closely physically associated with sensors. These observations suggest that genetic linkage of *NRC0* with sensors is ancestral [44], but the physical clustering of NRC genes may be influenced by species-specific genomic rearrangements.

We expanded Goh et al. (2024) analyses by determining the functional dependency of lettuce NRC sensors on NRC helpers using a transient co-expression system in *N. benthamiana* [43]. Of the 15 NRC-S tested, 9 triggered hypersensitive cell death when co-expressed with at least one lettuce NRC helper. The phylogenetic analysis revealed a shift in helper dependency among lettuce sensors: group 1 sensors exclusively rely on LsatNRC0, while group 3 sensors depend solely on Ast-LsatNRC1. Group 2 sensors can signal through both LsatNRC0 and Ast-LsatNRC1. This gradual evolutionary shift in helper dependency provides us a simplified system to study the diversification and function of NRC-S and NRC-H.

Previously, we demonstrated that, in monocot NLR pairs, helpers could form resistosome-like oligomers when modeled using AlphaFold 3, whereas sensor NLRs were unable to form similar structures [23]. However, whether this finding extends to dicots and NLR networks remained unclear. In this study, none of the tested NRC-S triggered cell death when expressed on its own. Multiple studies have also shown that, NRC-S cannot independently induce cell death or form higher-order complexes before or after activation [39,41,42]. Given these observations, we modeled NRC-H and NRC-S as hexameric complexes using AlphaFold 3. Similar to results with sensor-helper pairs in rice, NRC helpers were predicted to form resistosomes, whereas NRC-S failed to yield meaningful or high-confidence structural predictions. Combined with our functional data and biochemical evidence from other NRC-S, this finding is consistent with the view that NRC-dependent sensors have lost the ability to form resistosomes [39–41]. However, further structural studies on NRC-S in their resting and active states are needed to elucidate the mechanisms underlying sensor activation and sensor-helper interactions.

Our selection analyses revealed that NRC-H generally show low variability and lack signature of positive selection, consistent with helper NLRs like ADR1, which are classified among low-variability NLR classes [37,70,71]. In contrast, NRC-S demonstrate group-specific variation patterns. Group 1 sensors, reliant on NRC0, show high variability in the CC domain and the N-terminal part of the LRR domain, aligning with previous findings by Sakai et al. (2024) [44]. The cause of this diversification—whether due to relaxed selection or co-evolution with pathogens—remains unclear. Group 2 sensors exhibit generally low variation and can signal through both LsatNRC0 and Ast-LsatNRC1. Group 3 sensors, dependent on Ast-LsatNRC1, display highly variable regions concentrated on the concave surface of the LRR domain, with numerous sites under positive selection, resembling highly variable NLRs like RPP1 in *Arabidopsis thaliana* [70]. The observed physical clustering within group 3 suggests that recent tandem duplication events and accelerated co-evolution with pathogens may have driven their diversification [50]. Together, these analyses support a model in which selective pressure on NRC-S is group-specific, with positive selection acting most strongly on Ast-LsatNRC1-dependent group 3 sensors, while NRC-H remain under purifying selection. We note, however, that the recent duplication history and uneven comparison sizes could introduce phylogenetic non-independence and sampling bias into the entropy and selection analyses, and these patterns should be interpreted with this caveat in mind.

**Fig 6:**
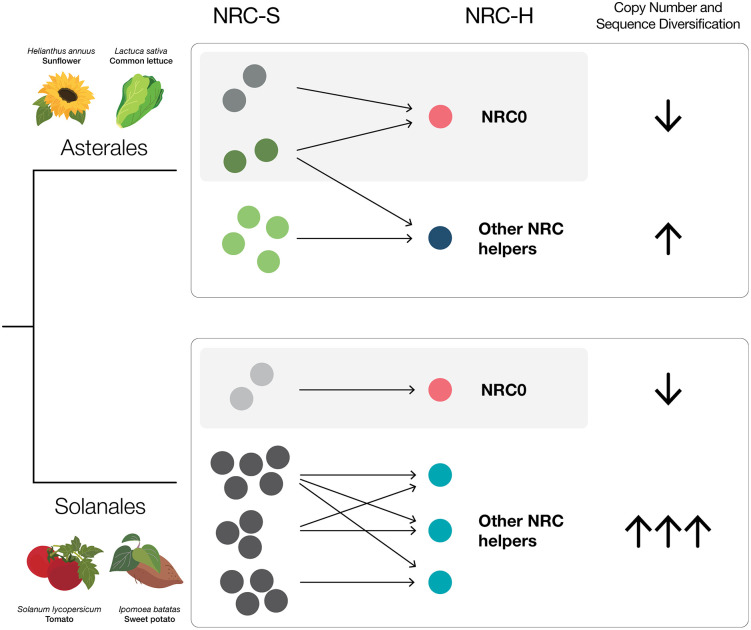
Evolutionary constraints and diversification patterns in NRC helpers and sensors. In our proposed model of the NRC network NRC-S dependent on NRC0 are characterized by lower copy number and are under lower positive selection, likely due to evolutionary constraints associated with their dependency on NRC0. In contrast, other NRC-H and the NRC-S depending on them have undergone significant expansions, exhibiting higher copy numbers and diversification rates. Representative species from each order are shown. NRC-H: NRC helper NLRs; NRC-S: NRC-dependent sensor NLRs.

Why have genes in within the NRC network evolved differentially? The evolutionary trajectories of different sub-types are probably shaped by their distinct roles, sensor-helper dependencies, and interactions with pathogens. The contrasting conservation of NRC0 and its functionally connected sensors versus the diversification of other NRC-H and NRC-S highlights varying selective pressures acting on these groups. NRC0 and its dependent sensors are highly conserved across most Asterid lineages [43,44] (Fig 6). The limited copy number and conservation of NRC0-dependent sensors, particularly in their LRR domains (which usually mediate pathogen recognition), may reflect detection of conserved effector molecules or guarding essential host components that are targeted by effectors, analogous to the ZAR1-ZRK system [85]. This conservation may limit the diversification of corresponding plant receptors. Such constraints could also arise from the need to maintain compatibility with NRC0, ensuring that evolutionary changes do not disrupt functional interactions or downstream signaling processes. In contrast, other NRC helpers and their associated sensors, especially those in lineages like Solanales, show greater diversification and expansion. This may stem from selective pressures such as detection a broader range of pathogen effectors, evading suppression, or introducing redundancy in signaling pathways [31,53–55,86]. Non-NRC0-dependent sensors, often physically cluster together, exhibit strong signatures of positive selection, particularly in their LRR domains, which likely reflects their engagement in a faster co-evolutionary arms race with pathogens compared to NRC0-dependent sensors.

In summary, our study highlights the contrasting evolutionary trajectories between helper and sensor NLRs, as well as among distinct sensor NLR phylogroups within the NRC network. The NRC network mediates robust immune responses against a diverse array of pathogens, including bacteria, fungi, oomycetes, nematodes, and viruses. Genes such as *NRC6* demonstrate specialization by providing tissue-specific or pathogen-specific resistance [36]. This multi-paced evolution of helpers and sensor phylogroups has likely granted the network the flexibility to diversify and counter a broad spectrum of pathogens while allowing specialization and potential adoption of novel immune functions. The simple Asterales NRC network in lettuce provided insights into a proposed model of how a shift in helper dependency may have facilitated sensor diversification and expansion distinct from their more conserved ancestral counterparts (Fig 6). Furthermore, our findings illustrate how closely related NLRs with a functional connection, such as lettuce NRCs, can exhibit varying diversification patterns, similar to those seen in more distantly related genes or in NLRs displaying allelic diversity within a single locus, such as RGC2 [69]. Addressing the molecular mechanisms governing helper specificity and sensor-helper interactions in future studies will illuminate how these immune receptors have adapted and evolved across plant lineages. This will facilitate breeding and immune engineering efforts in Asterid crop species.

## Methods

### Extraction of NRC network sequences

A total number of 29,261 NLRs were compiled from the NLRtracker output of 39 Solanales and 29 Asterales *de-novo* annotated high-quality genome assemblies from NCBI (https://www.ncbi.nlm.nih.gov/datasets/) and a *S. melongena* chromosome-level genome assembly from GWH genome warehouse datasets [87] (S1 Data) [73,88]. Based on the simple domain annotation from NLRtracker we only kept NLRs with “CNL”, “CNLO”, “CN”, “OCNL”, “CONL”, “NL”, “NLO”, “ONL”, “BCNL”, “BNL”, “BCN”, “BCCNL”, “BNLO”, “BOCNL”, “RNL”, “TN”, “TNL”, “TNLO”, “TNLJ” domain architectures. This led to keeping 25,042 NLRs. The remaining NLRs were then deduplicated using an R script (remove redundancy based on 100% sequence similarity) to keep 24,518 non-redundant sequences. Finally, based on NLRtracker domain output, any entry with NB-ARC domain shorter than 250 amino acids and longer than 400 amino acids were removed. This led to a final 21,232 sequences that was used for the phylogenetics analysis.

To construct a phylogenetic tree of the extracted NLRs, the NB-ARC domain sequences were aligned with RefPlantNLR NB-ARCs using FAMSA v2.2.2 [default options] [89]. The alignment was then used to construct a phylogenetic tree using FastTree v2.1.11 [-lg] [9,75]. The NRC network along with CcRPP1 and its orthologs, including 7,040 sequences, was extracted based on the presence of reference sequences from a well-supported branch containing NRC-H and NRC-S. The NB-ARC domain of the extracted sequences were aligned using MAFFT v7.525 [90]. The alignment was used to construct a new phylogenetic tree in the same way as before. The resulting tree was rooted at the CcRPP1 phylogroup.

### Phylogenetics and gene distance analyses

Plant taxonomy trees and taxa divergence times were obtained from TimeTree 5 database (timetree.org) [74]. 122 NRC sequences including helpers and sensors from *Lactuca sativa*, *Lactuca saligna*, *Lactuca virosa*, *Cichorium intybus*, *Helianthus annuus*, *Chrysanthemum lavandulifolium*, *Cynara cardunculus*, and *Codonopsis lanceolata* present in the dataset were extracted based on phylogeny. The NB-ARC sequences of extracted proteins were aligned with AtZAR1, NbZAR1, NbNRC2, SlNRC0, as references, using MAFFT v7.525 [--anysymbol] [90]. FastTree v2.1.11 [-lg] was used to generate the tree of NRC network in the mentioned species [75]. We then used IQtree v2.3.0 [-B 1000 -m MFP] to generate a phylogenetic tree of 40 sequences in the NRC network in the *Lactuca* genus [76,91]. The resulting tree was divided to helpers, sensor group 1, sensor group 2, and sensor group 3 based on branch lengths and reference sequences.

To obtain the physical location of the genes on the chromosome, first the GFF output from Helixer was simplified using GffRead [default options] and then imported into R using rtracklayer package [92,93]. The physical distances between genes was calculated using a custom R script and gene coordinates were exported to be visualized on chromosomes using MG2C online tool [94]. The output was then visualized manually.

### Linking previous lettuce NLRome classifications to the new annotation

Lettuce NLRome IDs from Christopoulou, et al., 2015, and Lettuce V8 genome assembly from Phytozome 13 were extracted [64,95,96]. We then used BLASTP as part of BLAST+ v2.16.0 to identify corresponding gene identifiers in the Phytozome 13 annotation to the new Helixer annotation [97]. The BLAST output was then processed in R and the gene coordinates, major resistance loci information, and RGC groups were retrieved for the new Helixer annotation. The corresponding genes between the Phytozome 13 and Helixer annotations were identified based on BLAST hits with >98% sequence identity and bit scores above 1500 (S12 Data).

The same approach was used to retrieve corresponding sequences from the RefSeq reference annotation of Lsat_Salinas_v11 (GCF_002870075.4) [98] (S12 Data).

### Agroinfiltration and cell death assays

The NRC sequences used in the cell death assays were originally retrieved from the Phytozome 13 annotation. These sequences were cross-checked against the Helixer annotation, with two sequences absent from the latter. All tested sequences are listed in S8 Fig.

To test the functionality of lettuce NRCs, annotated genes were cloned using the Golden Gate Modular Cloning (MoClo) kit [99] and the MoClo Plant Parts kit [100]. In brief, coding sequences were synthesized by GENEWIZ as Golden Gate Level 0 pICH41155 modules and subsequently transferred to the binary vector pICH86988 through *Bsa*I digestion (Weber et al., 2011). Native *Bsa*I sites in the original sequences were domesticated as needed. In addition to wild-type clones, a D-to-V mutation within the MHD motif was introduced to generate autoactive versions [15] of the lettuce NRCs. Verified clones were transformed into *A. tumefaciens* strain GV3101 pMP90 for ectopic expression in *N. benthamiana*.

For cell death assays, wild-type and nrc2/3/4 *N. benthamiana* plants were grown in a controlled environment growth chamber at 22–25°C with a 16-hour light period and 45%/65% relative humidity during the light/dark cycle. Light intensity was approximately 200 µmol/m²/s. Four- to five-week-old plants were used for agroinfiltration, following the methods described by Bos et al. (2006) [101]. The final OD_600_ of all Agrobacterium suspensions was adjusted to 0.5 in infiltration buffer (10 mM MES, 10 mM MgCl_2_, and 150 µM acetosyringone, pH 5.6). This concentration was used for both expressing clones individually and co-expressing NRC-H and NRC-S of interest. The cell death phenotype was imaged 4–5 days post-agroinfiltration.

### Sequence polymorphism and selection analyses

Nucleotide sequence of the NRC sequences were obtained using a custom python script from a compiled CDS file of the annotated genomes. Codon-based nucleotide alignments were generated for each NRC-H and NRC-S phylogroup generated using MACSE v2.07 [-prog alignSequences -seq] [102]. Positive selection tests were conducted on resulting alignments using CodeML in PAML v4.10.7 [79]. For each NRC phylogroup (helpers, sensors C1, C2, and C3), two site models were evaluated: M8a (beta + ω = 1) and M8 (beta + ω). Likelihood ratio tests (LRT; 2ΔL = 2(lnL_alternative – lnL_null)) were performed comparing M8a vs M8 (df = 1, χ² critical value = 6.63 at 1% significance). Sites under positive selection were identified using the Bayes Empirical Bayes (BEB) method [80] under the M8 model, with posterior probabilities > 0.95 (and the more stringent > 0.99) used to indicate strong support.

To compare average selective pressure across NRC subclades, we ran PAML branch model 2 in CodeML (PAML v4.10.7; model = 2, NSsites = 0, CodonFreq = 2) [79]. assigning each of the four subclades (helper, sensor groups 1, 2, and 3) its own ω parameter via $1–$4 labels placed at each subclade’s most recent common ancestor in the rooted tree. This four-ratio model was tested against the one-ratio model (M0; model = 0) by likelihood ratio test (df = 4). Per-subclade tree dS lengths were obtained by summing the M0 branch-level dS estimates over each subclade’s stem and internal branches. The free-ratio model (M1; model = 1) was also run for exploratory visualisation of branch-level heterogeneity (S10 Fig) and is not used for hypothesis testing; in this model, branches with ω ≥ 10 reflect low synonymous substitution rates (dS ≈ 0) and are flagged as ambiguous rather than indicative of positive selection.

Pairwise nonsynonymous (*d*_N_) and synonymous (*d*_S_) substitution rates were estimated for full-length sequences and individual domains (CC, NB-ARC, and LRR) in each phylogroup using the Nei and Gojobori (1986) method [81,82]. These rates were extracted from the “2NG.dN” and “2NG.dS” outputs of CodeML using a custom R script. All figures were generated with the ggplot2 package in R [103].

### Protein structure prediction and mapping Shannon entropy to protein structures

AlphaFold 3 [Seed = 1, Ligand = ADP] was used to predict the monomeric structures of representative sequences from each phylogroup (S9 Data) [78]. For oligomeric structures, AlphaFold 3 with [Ligand = OLA x 50] was used to model sequences (S9 Data). First model for each sequence was modeled with Seed = 1. Four other replicates were modeled for each sequence with random seeds. Model confidence and metadata were processed and plotted with R. All scripts available at [https://github.com/amiralito/lettuce_salad].

## Supporting information

S1 Fig Phylogenetics tree of NB-ARC domains from 21,645 Solanales and Asterales NLRs with RefPlantNLR as the reference. (DOCX)

S2 Fig Total number of NLRs, NRC network sequences, NRC0s, other NRC helpers, Rx-type sensors, SD-type sensors, and percentage of NRC sequences out of total number of NLRs in studied Asterales and Solanales species. (DOCX)

S3 Fig Phylogenetic tree of common lettuce (*Lactuca sativa*) NLRome featuring 359 sequences. (DOCX)

S4 Fig Physical map of *Lactuca saligna* and *Lactuca virosa* NRC sequences. (DOCX)

S5 Fig Phylogenetic tree of NRC sequences of Lactuca species (*Lactuca sativa*, *Lactuca saligna*, and *Lactuca virosa), Codonopsis lanceolata*, *Helianthus annuus*, *Cichorium intybus*, *Chrysanthemum lavandulifolium*, and *Cynara cardunculus.* (DOCX)

S6 Fig Physical map of *Codonopsis lanceolata*, *Helianthus annuus*, and *Cichorium intybus* NRC sequences. (DOCX)

S7 Fig Physical map of *Chrysanthemum lavandulifolium* and *Cynara cardunculus* NRC sequences. (DOCX)

S8 Fig Hypersensitive response (HR) cell-death assays of NRC-H and NRC-S. (DOCX)

S9 Fig Structural modeling of lettuce NRC-H and NRC-S sequences with AlphaFold 3. (DOCX)

S10 Fig Multi-Ratio Model branch-specific dN/dS (ω) values across the *Lactuca* NRC phylogeny. (DOCX)

S11 Fig Free-Ratio Model branch-specific dN/dS (ω) values across the *Lactuca* NRC phylogeny. (DOCX)

S12 Fig Pairwise dN and dS values for full-length, and CC, NB-ARC, and LRR domains of NLRs within the Lactuca NRC phylogroups. (DOCX)

S13 Fig Histogram of pairwise dN/dS ratios for full-length, and CC, NB-ARC, and LRR domains of NLRs within the Lactuca NRC phylogroups. (DOCX)

S1 Data List of genome assemblies and species used in this study. (XLSX)

S2 Data List of NLR sequences and metadata used for the phylogenetic analysis. (XLSX)

S3 Data List of NRC helper and sensor sequences used for the phylogenetic analysis. (XLSX)

S4 Data List of *Lactuca* NRC helper and sensor sequences and metadata. (XLSX)

S5 Data List of selected Asterales NRC helper and sensor sequences and metadata. (XLSX)

S6 Data Gene-distance matrix of NRC helpers and sensors. (XLSX)

S7 Data Raw cell death scores of tested lettuce NRC helpers and sensors in *Nicotiana benthamiana.* (XLSX)

S8 Data HMMsearch output of MADA motif for NRC sequences in *Lactuca* genus. (OUT)

S9 Data Model confidences for the predicted models used in different analysis. (XLSX)

S10 Data PAML results tables for the NRC phylogroups. (XLSX)

S11 Data Pairwise *d*_N_ and *d*_S_ values for the four NRC phylogroups. (XLSX)

S12 Data BLASTP output table of *L. sativa* NLRome from helixer against Phytozome 13 and RefSeq Lsat_Salinas_v11 proteomes. (XLSX)
