# Supplementary material for: Contrasting evolutionary patterns of helper and sensor NRC NLRs in lettuce reflect functional divergence following subfunctionalization
Source: PLoS Genet. 2026 Jul 16;22(7):e1012245. doi: 10.1371/journal.pgen.1012245 (PMC13390941; doi:10.1371/journal.pgen.1012245)
Supplement: S1 Fig — (DOCX) [file pgen.1012245.s001.docx]

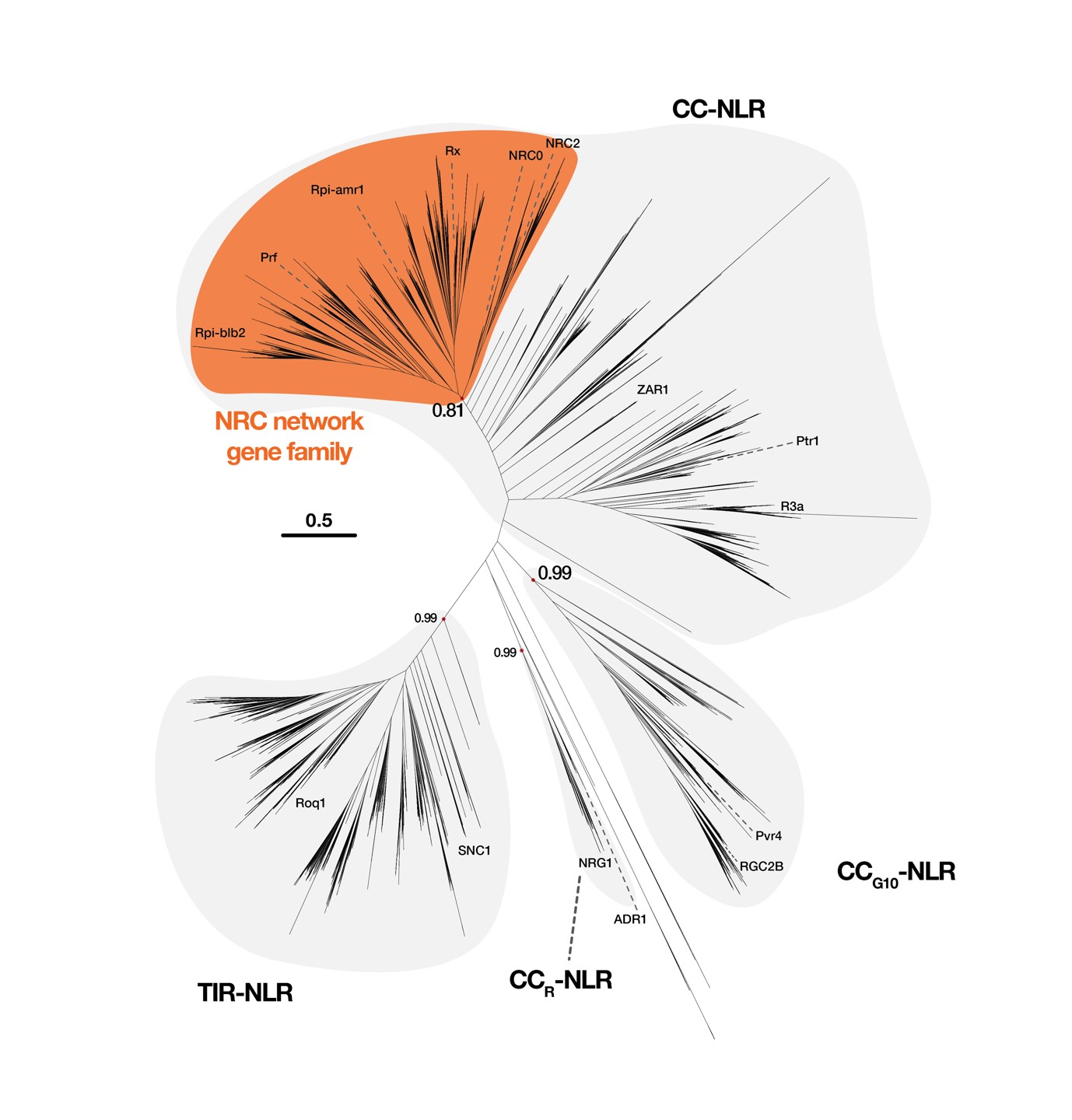


**Figure S1. Phylogenetics tree of NB-ARC domains from 21,645 Solanales and Asterales NLRs**  **with RefPlantNLR as the reference.**

NRC network, consisting of NRC helpers (NRC-H) and sensors (NRC-S), forms a well-supported phylogroup within the CC-NLR class. NLR classes are annotated on the tree.
