# Supplementary material for: Contrasting evolutionary patterns of helper and sensor NRC NLRs in lettuce reflect functional divergence following subfunctionalization
Source: PLoS Genet. 2026 Jul 16;22(7):e1012245. doi: 10.1371/journal.pgen.1012245 (PMC13390941; doi:10.1371/journal.pgen.1012245)
Supplement: S2 Fig — (DOCX) [file pgen.1012245.s002.docx]

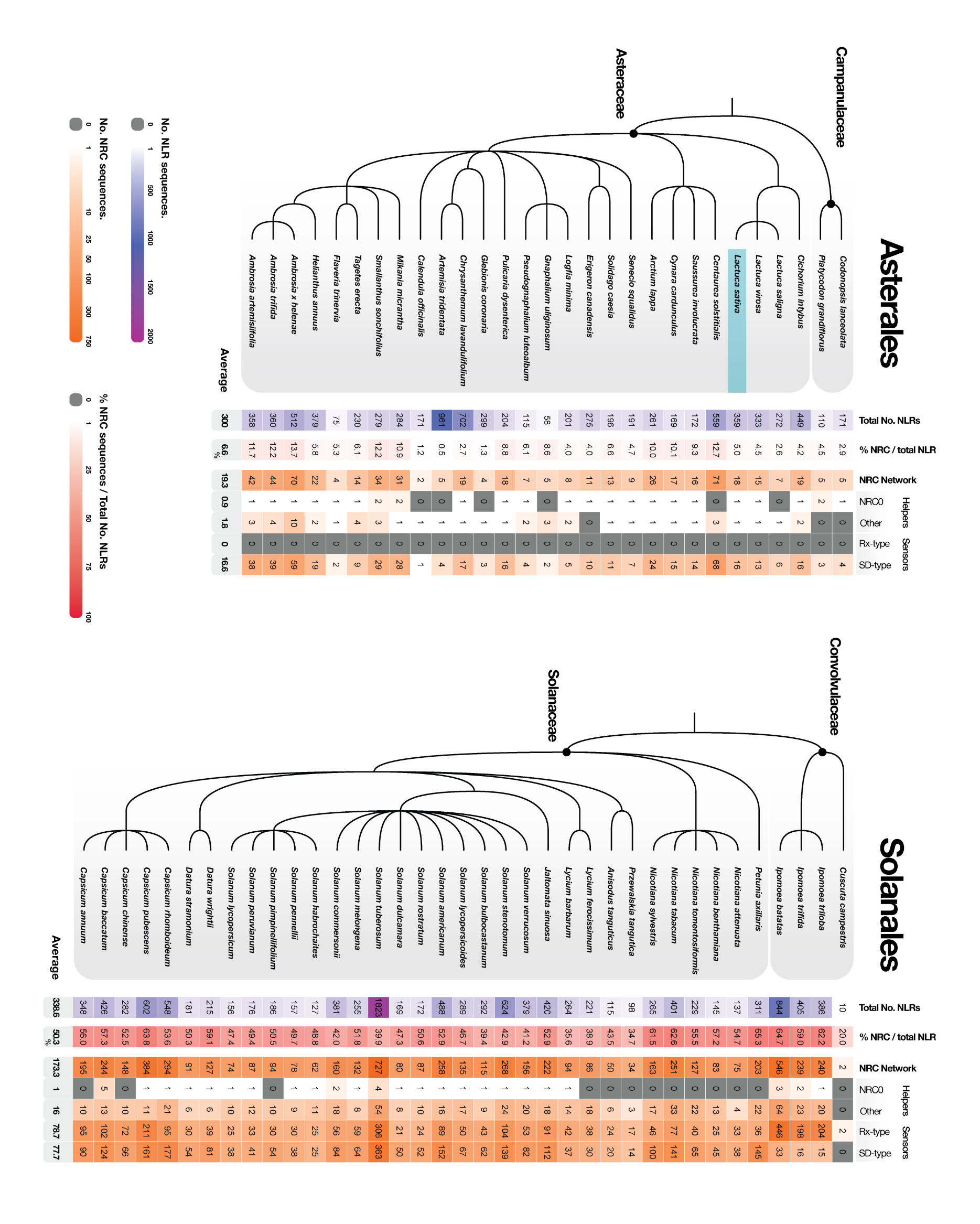


**Figure S2. Total number of NLRs, NRC network sequences, NRC0s, other NRC helpers, Rxtype sensors, SD-type sensors, and percentage of NRC sequences out of total number of NLRs in studied Asterales and Solanales species.**

*Lactuca sativa* (common lettuce) is highlighted in blue. NLR numbers were calculated after deduplication and filtering steps leading to a final set of 21,645 NLRs. SD: Solanaceous Domain
