# Supplementary material for: Contrasting evolutionary patterns of helper and sensor NRC NLRs in lettuce reflect functional divergence following subfunctionalization
Source: PLoS Genet. 2026 Jul 16;22(7):e1012245. doi: 10.1371/journal.pgen.1012245 (PMC13390941; doi:10.1371/journal.pgen.1012245)
Supplement: S3 Fig — (DOCX) [file pgen.1012245.s003.docx]

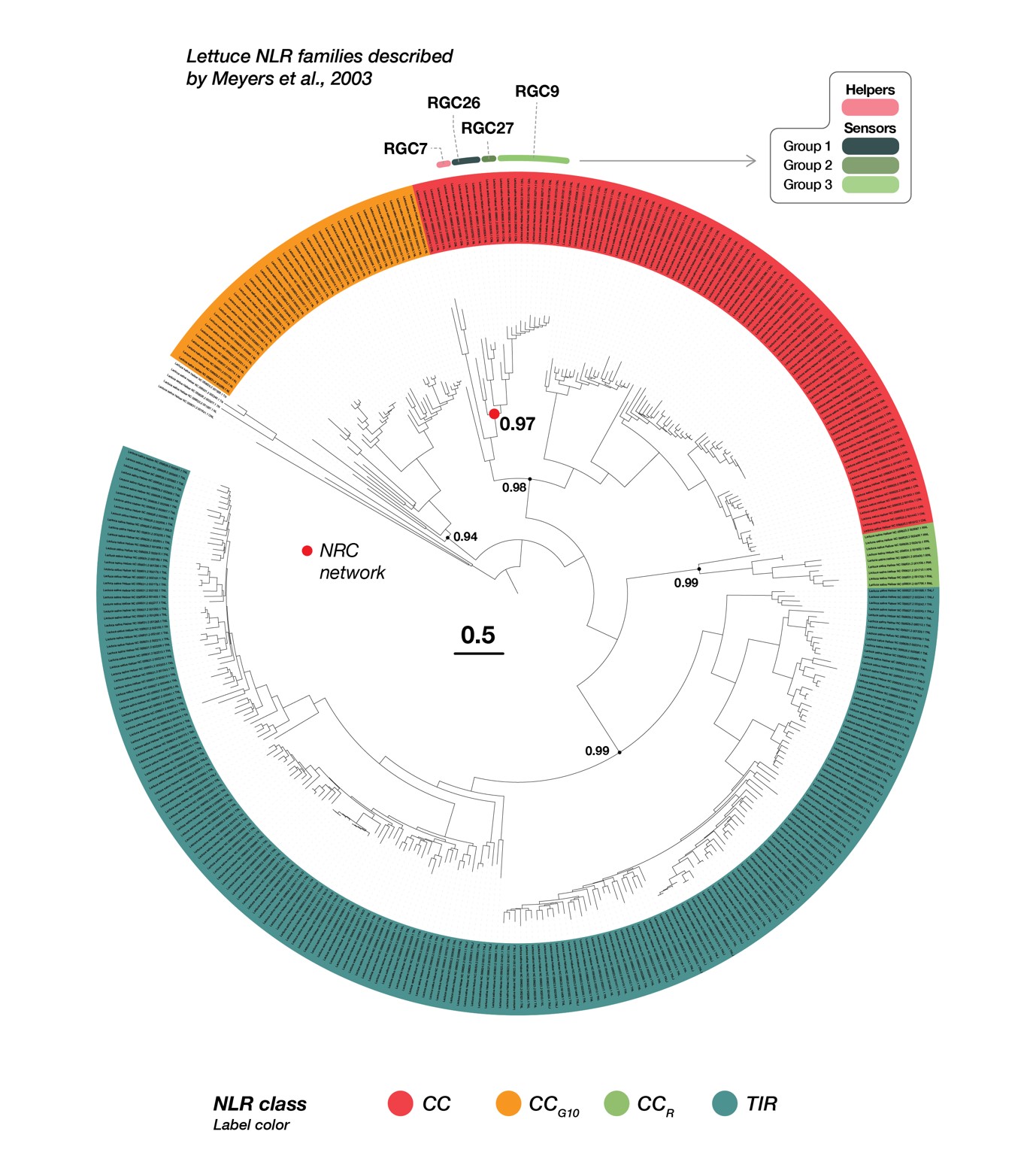


**Figure S3. Phylogenetic tree of common lettuce (*Lactuca sativa*) NLRome featuring 359 sequences.**

Different NLR clades are highlighted in color. Numbers on tree nodes indicate bootstrap values. Phylogenetic tree was made using FastTree 2 [75] based on NB-ARC domains and rooted at midpoint. NRC network superclade is highlighted with the red dot. NLR family classification by Meyers et al. (1998) is shown for the NRC network superclade [64,65]. RGC: Resistance Gene Cluster.
