# Supplementary material for: Contrasting evolutionary patterns of helper and sensor NRC NLRs in lettuce reflect functional divergence following subfunctionalization
Source: PLoS Genet. 2026 Jul 16;22(7):e1012245. doi: 10.1371/journal.pgen.1012245 (PMC13390941; doi:10.1371/journal.pgen.1012245)
Supplement: S5 Fig — (DOCX) [file pgen.1012245.s005.docx]

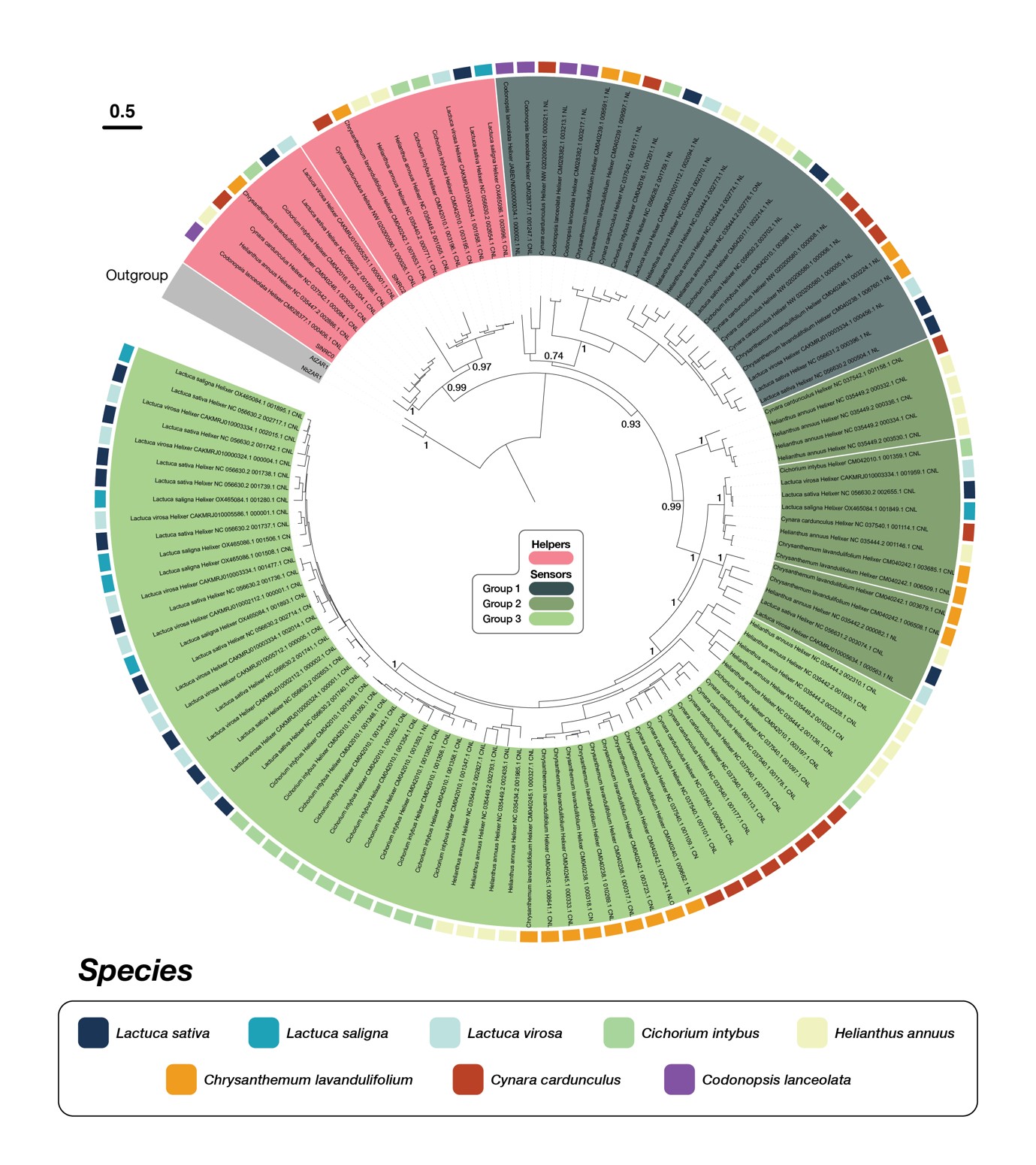


**Figure S5. Phylogenetic tree of NRC sequences of Lactuca species (*Lactuca sativa*, *Lactuca saligna*, and *Lactuca virosa*), *Codonopsis lanceolata*, *Helianthus annuus*, *Cichorium intybus*, *Chrysanthemum lavandulifolium*, and *Cynara cardunculus*.**

NRC-S are divided in three phylogenetic groups based on previous classification in the *Lactuca* genus. Numbers on tree nodes indicate bootstrap values. AtZAR1 and NbZAR1 were used as outgroup sequences. SlNRC0 and SlNRC2 were used as reference sequences. At: *Arabidopsis thaliana*; Nb: *Nicotiana benthamiana*; Sl: *Solanum lycopersicum*.
