# Supplementary material for: Contrasting evolutionary patterns of helper and sensor NRC NLRs in lettuce reflect functional divergence following subfunctionalization
Source: PLoS Genet. 2026 Jul 16;22(7):e1012245. doi: 10.1371/journal.pgen.1012245 (PMC13390941; doi:10.1371/journal.pgen.1012245)
Supplement: S6 Fig — (DOCX) [file pgen.1012245.s006.docx]

#
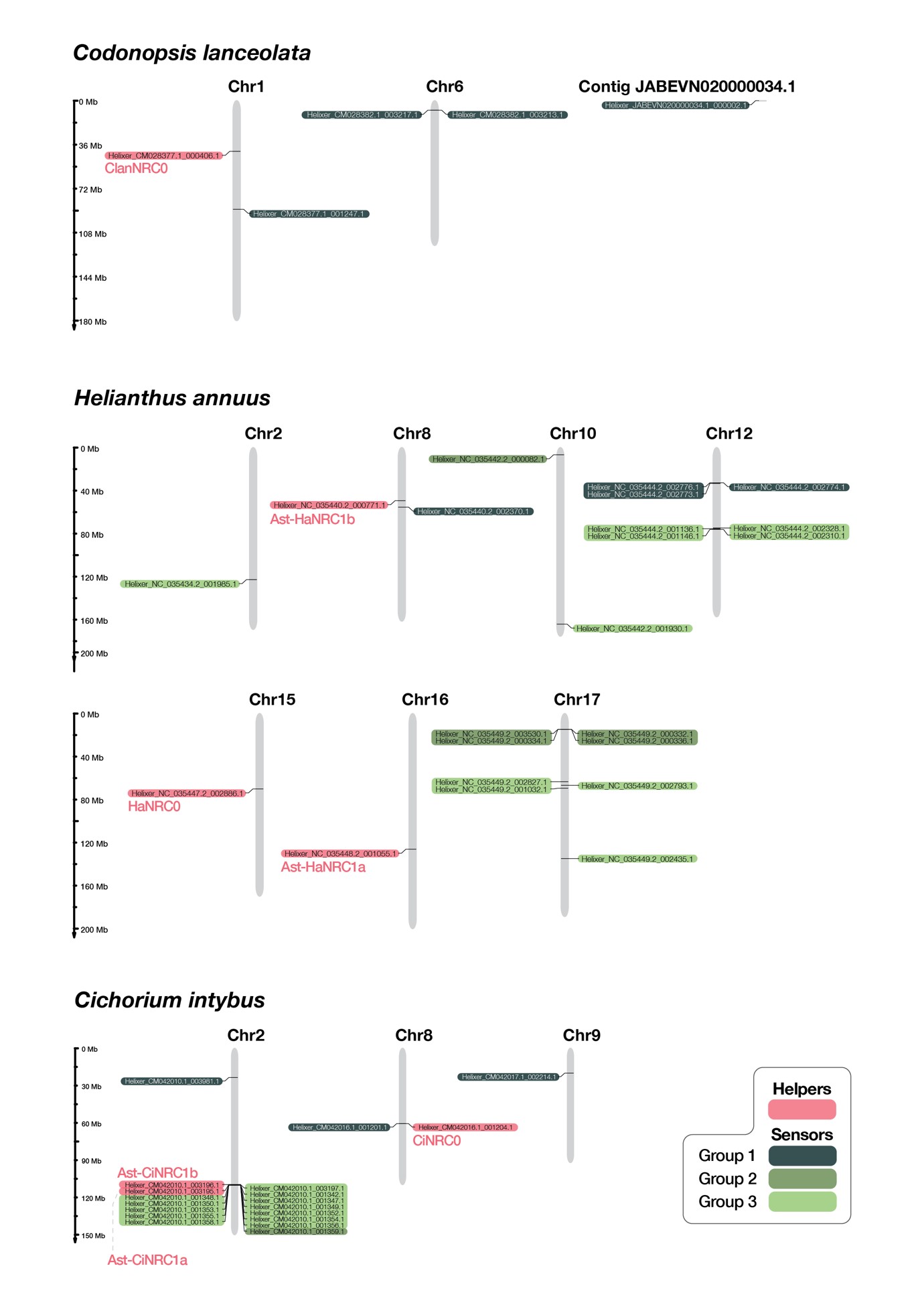


**Figure S6. Physical map of *Codonopsis lanceolata*, *Helianthus annuus*, and *Cichorium intybus***  **NRC sequences.** Ast: Asterales.
