# Supplementary material for: Contrasting evolutionary patterns of helper and sensor NRC NLRs in lettuce reflect functional divergence following subfunctionalization
Source: PLoS Genet. 2026 Jul 16;22(7):e1012245. doi: 10.1371/journal.pgen.1012245 (PMC13390941; doi:10.1371/journal.pgen.1012245)
Supplement: S8 Fig — (DOCX) [file pgen.1012245.s008.docx]

#
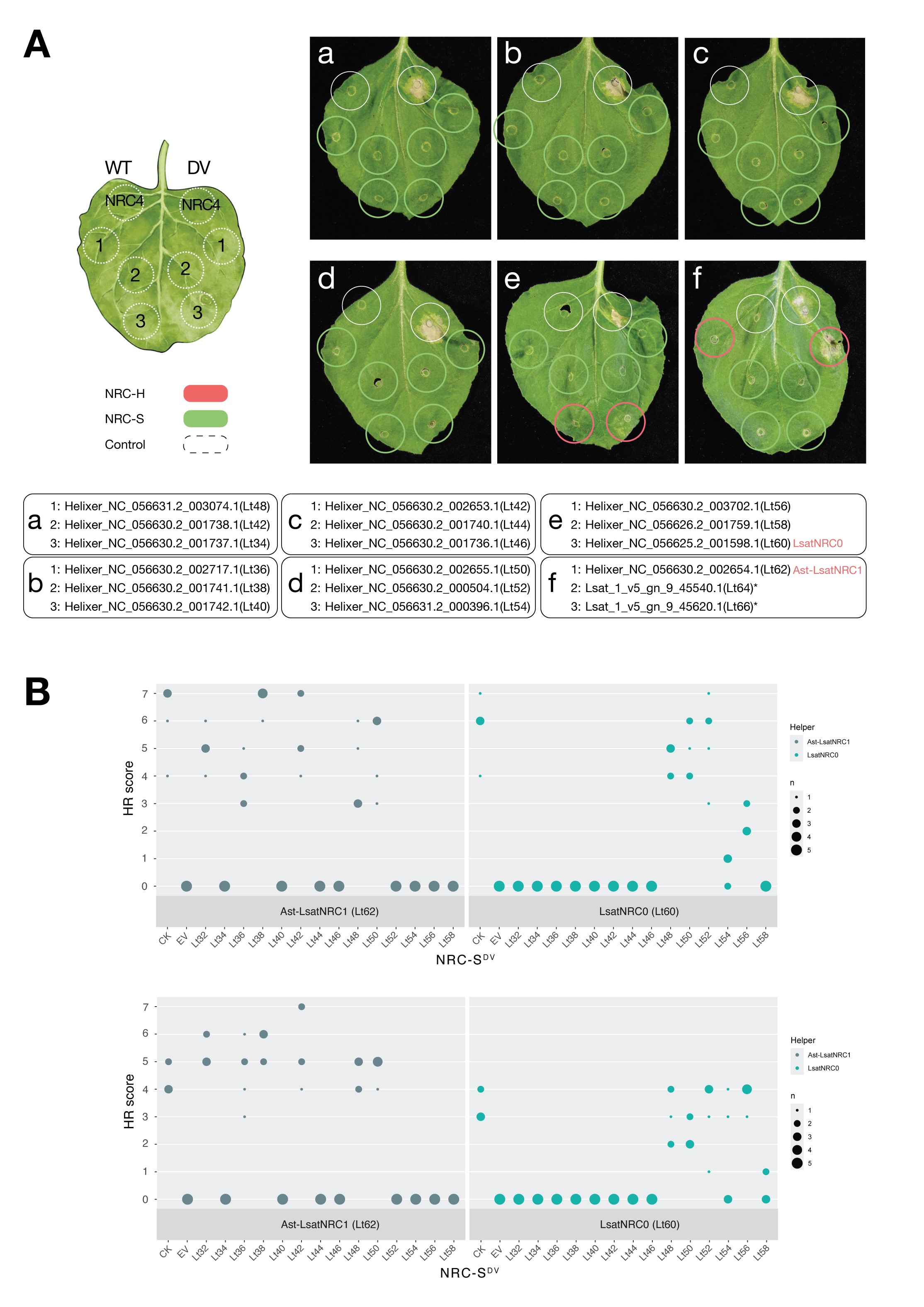


**Figure S8. Hypersensitive response (HR) cell-death assays of NRC-H and NRC-S.**

1. Representative *N. benthamiana* leaves expressing wild-type (WT) or MHD mutated (DV) NRC-H and NRC-S, photographed at 5 days post agroinfiltration. NRC4^WT^ and NRC4^DV^ were infiltrated here as negative and positive controls for HR, respectively. Sequences marked with star (*) are from Phytozome 13 annotation not included in the Helixer annotation. Ast: Asterales.

Quantification of co-agroinfiltration HR assays of NRC-H and NRC-S. NRC-S expressed as autoactive mutants with wild-type NRC helpers. CK: NRC4^DV^ infiltrated alone as positive control of HR. EV: NRC-H co-infiltrated with empty vector, as a negative control. Number (n) of data points are reflected as different sized circles. Two biological replicates are displayed as independent panels.
