# Supplementary material for: Contrasting evolutionary patterns of helper and sensor NRC NLRs in lettuce reflect functional divergence following subfunctionalization
Source: PLoS Genet. 2026 Jul 16;22(7):e1012245. doi: 10.1371/journal.pgen.1012245 (PMC13390941; doi:10.1371/journal.pgen.1012245)
Supplement: S9 Fig — (DOCX) [file pgen.1012245.s009.docx]

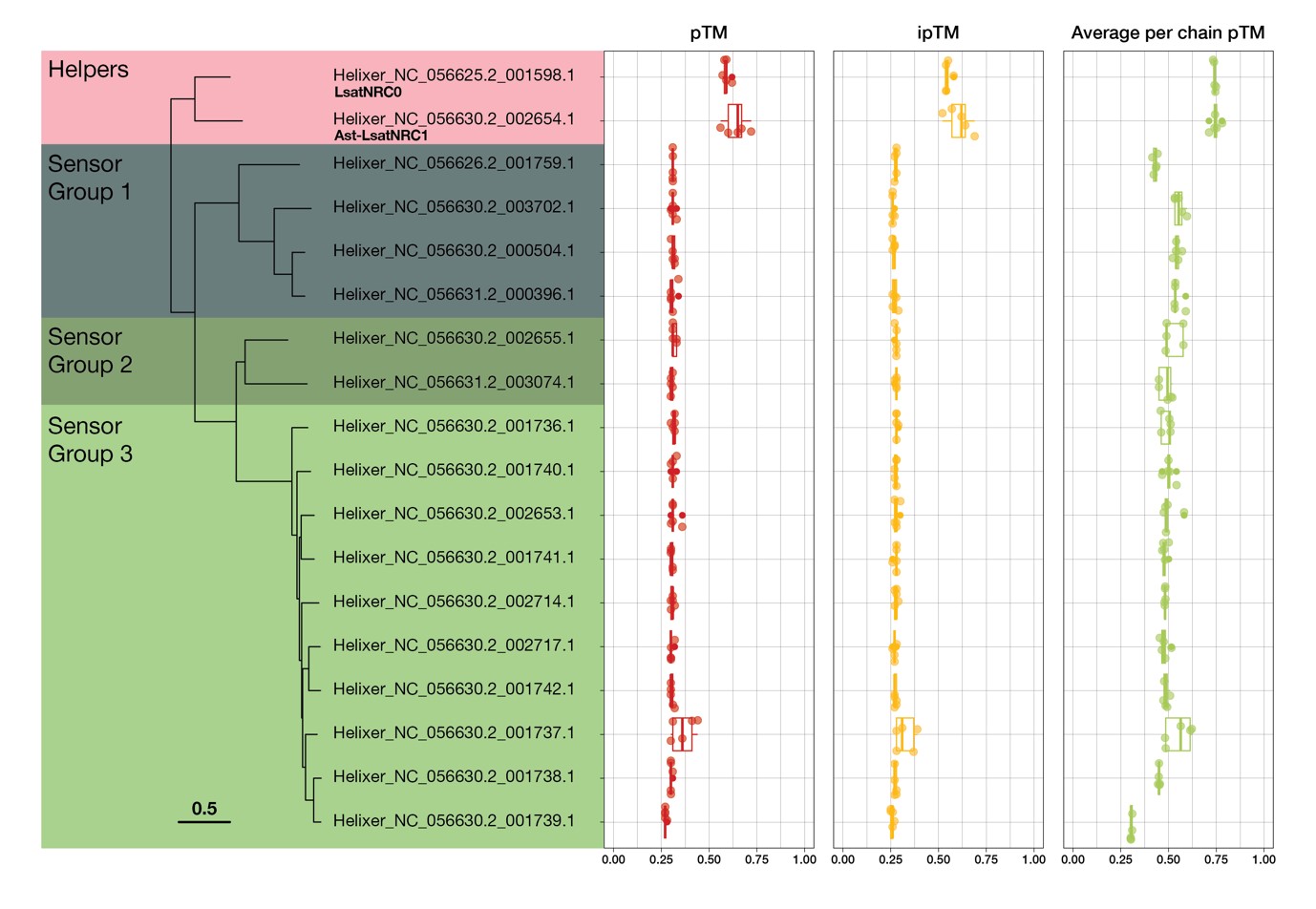


**Figure S9. Structural modeling of lettuce NRC-H and NRC-S clade sequences with AlphaFold**

**3.**

Simulations included 50 oleic acid molecules to approximate the plasma membrane. Key structural metrics, including pTM (predicted template modeling score for overall structure), ipTM (interfacespecific pTM), and per-chain pTM, are plotted for each protein (n=5).
