# Supplementary material for: Contrasting evolutionary patterns of helper and sensor NRC NLRs in lettuce reflect functional divergence following subfunctionalization
Source: PLoS Genet. 2026 Jul 16;22(7):e1012245. doi: 10.1371/journal.pgen.1012245 (PMC13390941; doi:10.1371/journal.pgen.1012245)
Supplement: S10 Fig — (DOCX) [file pgen.1012245.s010.docx]

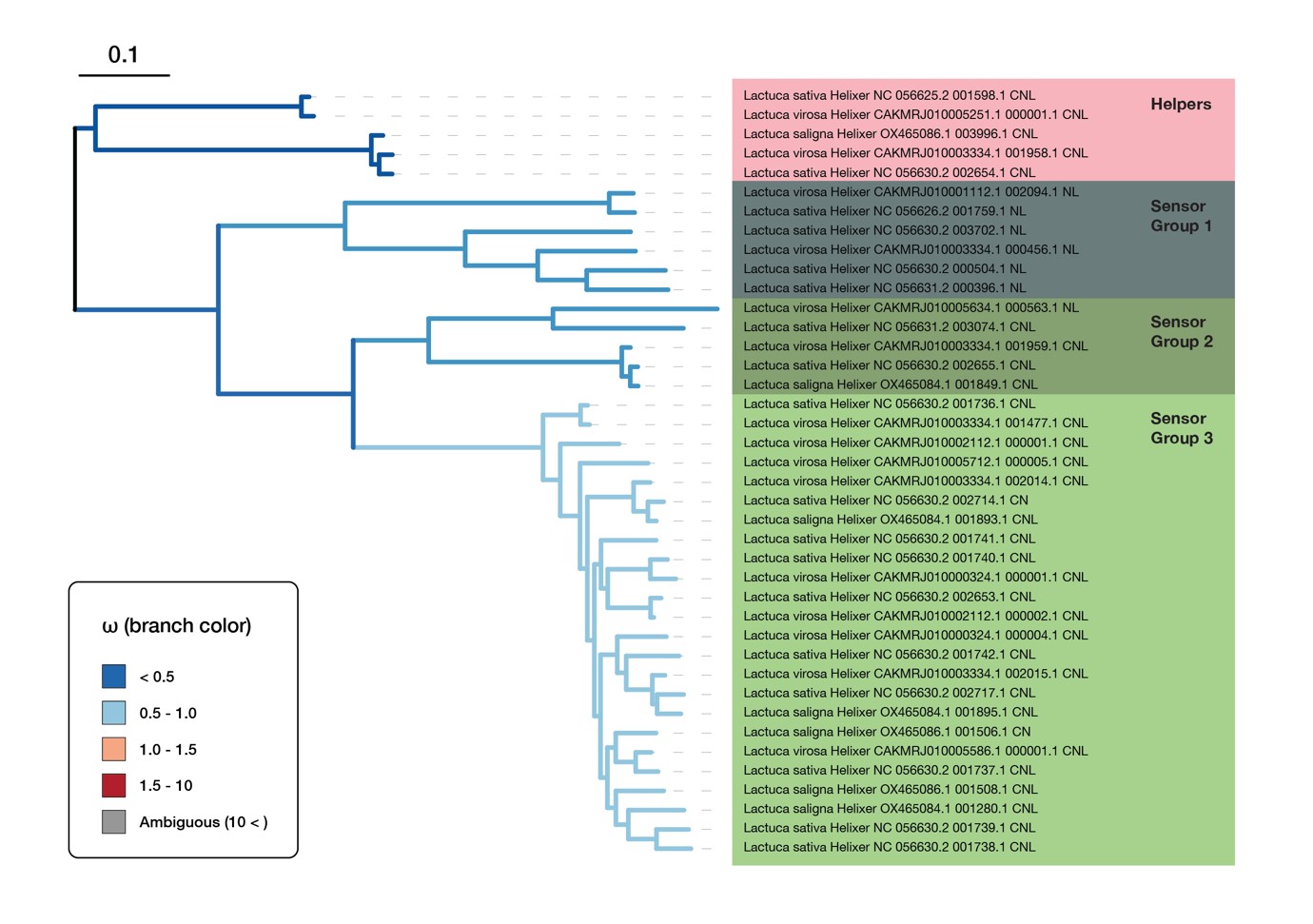


**Figure S10. Multi-Ratio Model branch-specific dN/dS (ω) values across the *Lactuca* NRC phylogeny.**

Phylogenetic tree of NRC-H and NRC-S with branches colored according to ω values estimated under the Multi-Ratio Model (M2) in CodeML. Branch colors indicate: ω < 0.5 (dark blue), 0.5 ≤ ω < 1.0 (light blue), 1.0 ≤ ω < 1.5 (light red), 1.5 ≤ ω < 10 (dark red), and ω ≥ 10 (grey, ambiguous due to low synonymous substitution rates). Branches with ω > 1 are annotated with their values.
