# Supplementary material for: Contrasting evolutionary patterns of helper and sensor NRC NLRs in lettuce reflect functional divergence following subfunctionalization
Source: PLoS Genet. 2026 Jul 16;22(7):e1012245. doi: 10.1371/journal.pgen.1012245 (PMC13390941; doi:10.1371/journal.pgen.1012245)
Supplement: S12 Fig — (DOCX) [file pgen.1012245.s012.docx]

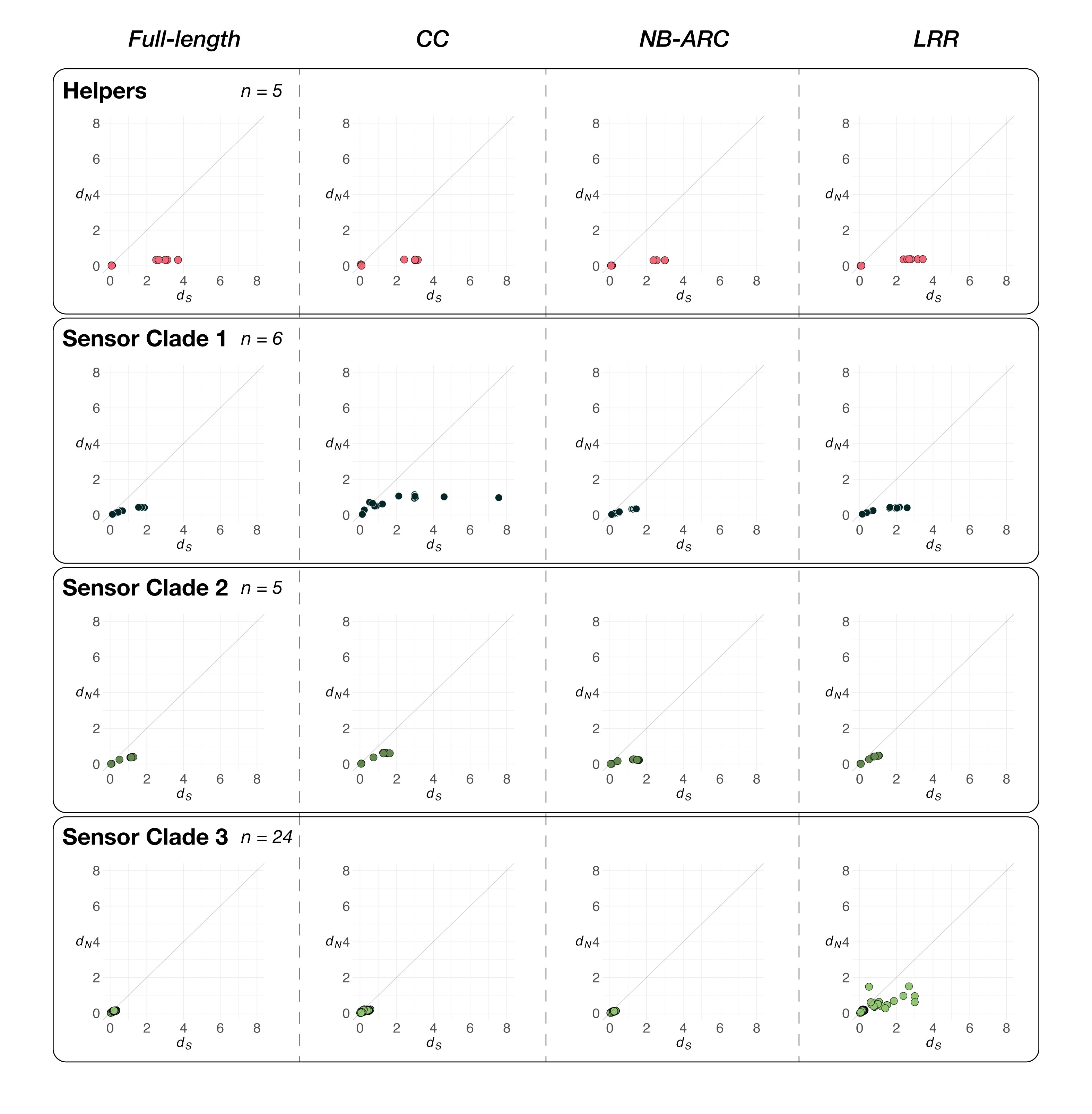


**Figure S12. Pairwise *d*_N_ and *d*_S_ values for full-length, and CC, NB-ARC, and LRR domains of NLRs within the *Lactuca* NRC phylogroups.**

The nonsynonymous (dN) and synonymous (dS) substitution rates were estimated using the approximate method of Nei and Gojobori (1986), implemented in the PAML software [79,82]. A diagonal line represents *d*_N_ = *d*_S_, indicating neutral selection. Points above this line correspond to positive selection (w = *d*_N_ / *d*_S_ > 1).
