# Supplementary material for: Contrasting evolutionary patterns of helper and sensor NRC NLRs in lettuce reflect functional divergence following subfunctionalization
Source: PLoS Genet. 2026 Jul 16;22(7):e1012245. doi: 10.1371/journal.pgen.1012245 (PMC13390941; doi:10.1371/journal.pgen.1012245)
Supplement: S13 Fig — (DOCX) [file pgen.1012245.s013.docx]

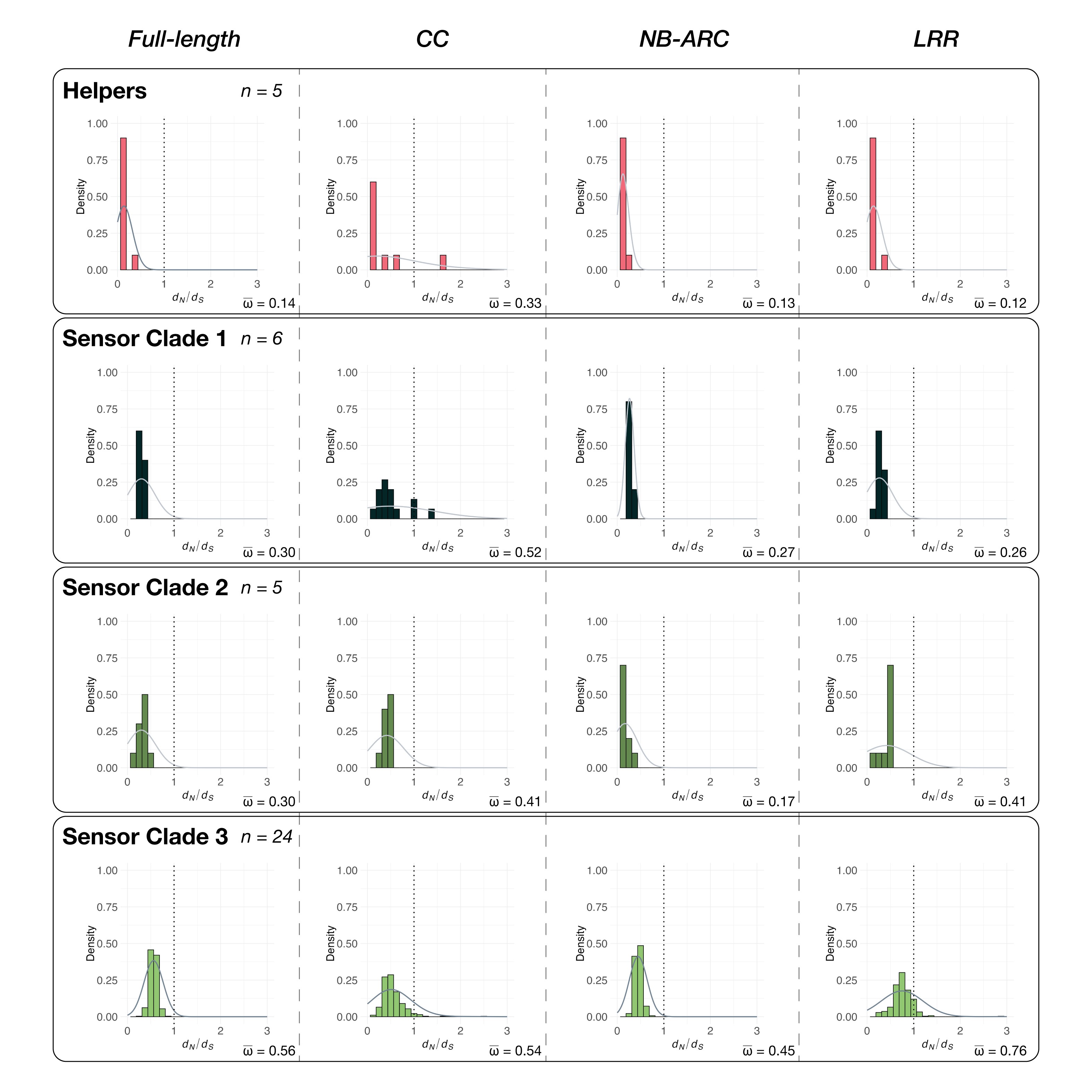


**Figure S13. Histogram of pairwise *d*_N_/*d*_S_ ratios for full-length, and CC, NB-ARC, and LRR domains of NLRs within the *Lactuca* NRC phylogroups.**

The nonsynonymous (dN) to synonymous (dS) substitution rate ratios were estimated using the approximate method of Nei and Gojobori (1986), implemented in the PAML software [79,82]. *d*_N_/*d*_S_ (w) = 1 is highlighted with dotted line. Average w for each graph is displayed at the bottom right corner.
