# Supplementary material for: Contrasting evolutionary patterns of helper and sensor NRC NLRs in lettuce reflect functional divergence following subfunctionalization
Source: PLoS Genet. 2026 Jul 16;22(7):e1012245. doi: 10.1371/journal.pgen.1012245 (PMC13390941; doi:10.1371/journal.pgen.1012245)
Supplement: Attachment — Submitted filename: PGENETICS_respond_to_reviews_v3.docx [file pgen.1012245.s027.docx]

**Respond to reviewers**

**Editor Comments:**

**We have now received four referee reports on your manuscript on immune receptors (NLRs) in lettuce. All reviewers found the study valuable and original, but they also raised several concerns, particularly regarding the statements about current knowledge on NLRs, as well as the use of evolutionary terminology and analyses. The referees recommend clarifying the scientific questions framing the manuscript, performing additional experimental validation, accounting for phylogenetic non-independence in the dataset, and using more appropriate methods to assess diversity and positive selection.

I also found the study very interesting and within the scope of PLOS Genetics; however, I concur with the reviewers’ comments and add further recommendations below. I strongly encourage resubmission provided the manuscript is revised accordingly. The reviewers also offer many excellent suggestions that should be carefully addressed.

More specific comments:**
**-The title and abstract will be difficult to understand for most readers of PloS Genetics, try to make it clearer for a broad range of readers. For example, explain what are helper and sensor NLRs, what you mean about "functional connections" and "networks". Overall, it is very difficult for non-specialist to understand the findings, the main take-home message and the significance for the broad readership of PloS Genetics.**

RESPONSE: We thank the Editor and modified the title accordingly. The revised title: “Contrasting evolutionary patterns in a genetic network of NLR immune receptors in lettuce reflect functional divergence” is more concise and replaces specialized terminology, such as “hierarchical”, “sensor”, and “helper” – with “functionally divergence”, which more clearly communicates the core evolutionary focus of the study to the broad readership.

**-Figure 2 and elsewhere: the term "phylogeny" should only be used for species relationships, not gene genealogies.**

RESPONSE: We thank the editor for this comment on the terminology. However, we respectfully disagree with this point. While we appreciate the distinction between species trees and gene trees, the term "phylogenetic" is widely and routinely used in the NLR literature and more broadly in molecular evolution to describe gene trees (e.g., "phylogenetic analysis," "phylogenetic tree," "phylogenetic clade"). This usage is well-established and unlikely to cause confusion, particularly in a study where the distinction between species relationships and gene genealogies is made clear by context. We prefer to retain the current terminology for consistency with the field.

**-L379: this is M&M not results**

RESPONSE: This is now fixed. Part of this section is moved to the materials and methods.

**--L380-386: awkward wording, explain better what are the models and what are these tests: what are you testing here exactly? -L388, L394, L396, L399: explain to the reader what one can infer from this**

RESPONSE: This now has been rephrased according to the new analysis and adapted for better explanation of the methods.

**-L508: comma after that
-L519: lack signature
-L67, L103: awkward wording, this sounds Panglossian: you cannot evolve a trait because it will allow future evolution, natural selection does not foresee anything and only operate among standing variation
-L135: comma after that if you keep the one after helpers
-L137: unclear what "differentially evolving" means**

RESPONSE: This is now change to “NLRs evolving at different rates”.

**-L144: unclear what "expansion" means here**

RESPONSE: This is now rephrased to “gene copy number”

**-L148: from the two, not both (species cannot belong to both orders)**

RESPONSE: we addressed all specific comments. We have revised the Introduction to remove teleological language (L100, 115, 117-118, paragraph L122 onwards), framing evolutionary changes as outcomes of selection acting on standing variation rather than forward-looking strategies. Specifically, phrasing such as “to prevent” or “developing redundancy” has been replaced with more accurate, reactive descriptions (e.g., “that minimize” or “presence of redundancy”) to better reflect the mechanisms of NLR evolution.

**-L150: unclear what "network superclade" means, L532: what does "network clade" mean?**

RESPONSE: L150, L532. We normally define the “NRC network superclade” as comprising of both sensor and helper NRCs. We have now rephrased the text for better clarity.

**-L155: what statistical test allows you to say "significantly"?**

RESPONSE: This is now changed to “much”.

**-L214: "genetically clustered together" is ambiguous, I had to read several times, it usually means clustering in a phylogeny, and the whole paragraph is about clustering in genealogies right?**

RESPONSE: L214. We refer to it as “genetically clustered” to emphasize physical proximity of the genes in the genome, keeping the phrasing consistent with previous NLR/NRC literature (Sakai et al. 2024).

**-Do not refer to figures in the discussion, they should have been explained well enough in the result section**

RESPONSE: We now removed all the citations to figures throughout the discussion apart from referencing Figure 7 as it is the conceptual model.

**ii) Figure 7. Please confirm whether you drew the images / clip-art within the figure panels by hand. If you did not draw the images, please provide (a) a link to the source of the images or icons and their license / terms of use; or (b) written permission from the copyright holder to publish the images or icons under our CC BY 4.0 license. Alternatively, you may replace the images with open source alternatives. See these open source resources you may use to replace images / clip-art:
-** [**https://commons.wikimedia.org**](https://commons.wikimedia.org) **-** [**https://openclipart.org/**](https://openclipart.org/)**.**

RESPONSE: The images in Figure 7 were created by the first author specifically for this manuscript and were not sourced from any third-party materials. Please let us know if any additional confirmation or documentation is required for author-generated figures under the journal’s CC BY 4.0 license.

**Reviewers' comments:**

**Reviewer #1: In this manuscript, Pai and colleagues explore the CC NLR Helper and Sensor networks outside of the solanales, namely in the Asterales. The authors identify sensors, which are a sister clade of the SD type sensors, albeit lacking an SD domain, and helpers, which cluster with NRC helpers from Solanales, in asterales genomes. The authors then focus on a set of 2 helpers (containing the MADA motif and able to elicit cell death / HR as autoactive variants) and 9 sensors from lettuce and show that autoactive mutants of the helpers elicit cell death, while autoactive versions of the sensors do not. However, when co-infiltrated with wild-type helpers, some (but not all) autoactive sensors trigger a cell death response. Interestingly, there is helper-sensor specificity, and sensors from different clades are able to elicit cell death with help from one (NRC0 or NRC1) helper, or in the case of sensor clade II with both helpers.
The authors then move towards a computational analysis using alphafold3, confirming previous observations that helpers are modeled as multimeric resistosome-like structures, while sensors are not. The authors then continue with a computational analysis of the helper and sensor sequences across the Lactuca genus, and observe diversification of sensors, with the CC domain diversifying in clade I sensors, and the LRR domain diversifying in clade III; while clade II is most conserved. The author then identify a number of sites that diversify as being under positive selection.

Comments:
Overall the work is presented clearly, the rationale is well explained, the findings are discussed in the context of relevant literature. However, I feel that the second half of the manuscript (Fig 4 onwards) would benefit from some further explorations:**

**It is not clear why only the experimentally validated NLRs from L. sativa are investigated using alphafold3, but the whole Lactuca genus is used for further phylogenetic and evolutionary analysis.**

RESPONSE: Given the computational constraints of modeling resistosomes, AlphaFold 3 analysis was limited to the validated network, as representatives of each clade.

**Similarly, as I understand, the interactions between sensor clades I II and III with the two helpers are inferred from the phylogeny for the other Lactuca species. I think it would be valuable to confirm these interactions experimentally, given that the authors discuss the observed diversification in the context of the sensor-helper network.**

RESPONSE: We thank the reviewer for the valuable suggestion. While we agree this would strengthen the study, these experiments require extensive *de novo* gene synthesis of large NLR genes, which currently exceeds our project budget. However, we believe that sharing insights focused on common lettuce remains highly beneficial for the community to understand the diversification of the NRC superclade in campanuliid lineages. To address the reviewer’s concern, we have added disclaimers to the text (L334-337, L625-628) stating that the interactions in related species are phylogenetic inferences that serve as a predictive model requiring future experimental validation.

**I suggest validating the interactions for all sensors & helpers used in the comparative analysis, also to confirm that the diversification does not alter sensor-helper specificities.**

RESPONSE: Although the reviewer raises a valid point about the molecular basis of sensor-helper specificity, this topic is too complex to include in the current paper and involves multiple domains and polymorphisms as currently being investigated in our lab. Basically, this topic is not yet ready for publication and would require much deeper analyses.

**Minor comments:
I disagree on the statement that "[..] AlphaFold 3 can distinguish between NRC-S and NRC-H based solely on sequence and their capacity to form higher-order resistosome-like oligomers." (l. 313 ) af3 has no concept of NLRs, NRCs, or helpers and sensors. All that this suggests is that NRC-S and NRC-H sequences differ (which is also evident form the presence / absence of the MADA motif), that as consequence of these differences, the sequences are modeled into different structures, and these structures can in turn be modeled into higher-order complexes in some cases, but not others. This may be useful for researchers to distinguish between NRC-S and -H, but this is not a distinction provided by af3.**

RESPONSE: We appreciate the reviewer’s feedback regarding the application of AlphaFold 3 (AF3). Our argument is that AF3 serves as a powerful diagnostic tool precisely because it translates primary sequence data into distinct, predictable structural outcomes and is independent of any prior phylogenetic classification or manual motif searching.

In our analysis, AF3 consistently predicted high-confidence hexameric resistosomes for NRC helpers, while NRC sensors consistently failed to yield meaningful higher-order structures. This consistent bifurcation in structural modeling provides an objective, sequence-based signature that aligns with the known loss of resistosome-forming ability in NRC-dependent sensors. By demonstrating that structural modeling can reliably predict functional categories without other parameters such as phylogeny or presence of particular motifs (e.g. MADA) beforehand, we show its value as a diagnostic framework for the community. For further demonstration on the topic, please refer to: Toghani, A., Frijters, R., Bozkurt, T.O., Terauchi, R., Kamoun, S. and Sugihara, Y. 2025. Can AI modelling of protein structures distinguish between sensor and helper NLR immune receptors? New Phytologist, 248:17-23.

**Reviewer #2: Review of the paper entitled “A hierarchical immune receptor network in lettuce reveals contrasting patterns of evolution in sensor and helper NLRs” by Pai H. et al. submitted to PLoS Genetics

This paper describes genetic diversity and evolution of a subset of NLR-type resistance proteins of Asterales plant group including lettuce. In the previous studies, the authors revealed the presence of NLR networks in Solanales consisting of the NRC-type NLRs. In this manuscript, the authors expanded NRC analysis to Asterales, which is a sister group of Solanales. Based on the genome sequences of 40 Solanales and 29 Asterales species, the authors classified ~20,000 NLR gene sequences, which revealed that representation of NRC-type NLR in Asterales NLRs is only 6.6% as compared to 50% in Solanales (Fig. 1; Fig. S1, S2). The Helper NRC (NRC-H) has two groups represented by Ast-LsatNRC1 and Lsat NRC0, whereas the Sensor NRC has three groups, Clade 1, 2 and 3 (Fig. 2A). Physical linkage of Helper and Sensor NRCs were analyzed (Fig. 2B; Fig. S4), which showed variable patterns. Function of Helper and Sensor NRCs in lettuce was tested using HR-like cell death assay using Nicotiana benthamiana transient expression assay (Fig. 3), revealing that the Helper Ast-LsaNRC1 is coupled with Clade-3 Sensors and the Helper LsaNRC0 with Clade-1 Sensors, while Clade-2 Sensors can function with both the Helpers.**

**NLR multimer formation was predicted by Alphafold3, which showed Helper NRCs may form “resistosome” structure, whereas Sensor NRCs may not (Fig. 4). Domain-wise amino acid diversity was studied for the Helpers and Sensors, showing that Clade-3 Sensor has highly variable amino acids, particularly in the LRR domain (Fig. 5). Also, signature of positive selection was detected in Clade-3 Sensor LRR domain (Fig. 6).**

RESPONSE: We thank the reviewer and we are pleased that they found our various analyses, including AlphaFold 3, to be a useful addition to the literature.

**NLR genes in plants are known to be highly variable due to their co-evolution with pathogen effector genes. However, how NLR genes evolve is not fully understood and the studies on NLR evolution are urgently needed. Genome sequences became available from a large number of plant species, opening an avenue to study the mode and tempo of evolution of NLRs. This timely paper addresses the diversity and evolution of NRC-type NLRs of Asterales. Phylogenetic analysis and classification of NRCs to Helper and Sensor NLRs were validated with functional analysis of HR-like cell death assay, and were further addressed by Alphafold-mediated prediction of multimer resistosome formation. The results of evolution analysis are interesting. Overall, the analyses and experiments are solid, and interpretation of data is appropriate.**

RESPONSE: We thank the reviewer for the kind words.

**Minor:
L53: which and plays – remove “and”
L83: Citation no. 39 is duplicated
L223: “chromosome 3” should be “chromosome 2”**

RESPONSE: We addressed all the minor corrections.

**Reviewer #3: I was pleased to review this manuscript on the evolutionary history and function of NRC (NLR Required for Cell Death) immune receptors in Asterales. The manuscript's strengths lie in the quality of the writing, the figures (though good eyesight is sometimes required), the structural predictions, and the analyses of functional connections between NRCs.**

RESPONSE: We thank reviewer and pleased to note that they enjoyed reading the paper.

**The manuscript's weaknesses primarily concern the evolutionary analyses, and the description of the state of knowledge on NLR evolution. I present below several ideas that could help to refine and strengthen the analyses and conclusions.

State of knowledge about NLR evolution

L40: “NLR proteins represent one of the most diverse protein families, with different NLR clades evolving at varying rates [5,6].” None of the cited papers support the claim that NLR proteins represent one of the most diverse protein families. Both papers are based on the same Arabidopsis RenSeq data and do not compare NLR with other genes. The Progozhin et al. study does not really measure evolutionary rates, since it is based on the construction of groups of sequences that are not necessarily orthologous and on a metric (per-codon Shannon entropy) that is not directly a measure of evolutionary rate. The few, most advanced analyses of NLRs vs other genes’ diversity that I am aware of are those of Bakker et al. 2006 Plant Cell (doi: 10.1105/tpc . 106.042614) and** **Gladieux et al. 2024 Curr Biol (doi: 10.1016/j.cub.2024.07.061), which are not cited in the manuscript.**

RESPONSE: We added the articles suggested by the reviewer.

**Clade

Futuyama & Kirkpatrick 2023, Evolution, 5th edition:
Clade: The set of species descended from a particular ancestral species.
Gene family: Two or more loci with similar nucleotide sequences that have been derived from a common ancestral sequence and that have diverged to at least some degree in their functions.

None of what is described as a clade in this manuscript is a clade in evolutionary biology terms. I can’t really blame the authors, as several previous papers on the subject used this nomenclature. However, it’s particularly puzzling that the NRC aficionados use the term “clade” to refer to a “gene family”.**

RESPONSE: We note the comment of the reviewer, but we stick to the usage of clade to refer to a specific gene cluster that are monophyletic and clearly descended from an ancestral sequence. The definition of Futuyama & Kirkpatrick is outdated and doesn’t fit with the current usage in phylogenomics. However, to avoid any confusion we now define our meaning of clade in the text and methods: “*The NRC network, a monophyletic clade consisting of NRC-H and NRC-S (hereafter referred to as NRC network superclade), likely originated ~100 million years ago within the superasterid lineage of land plants and has expanded massively in the lamiid lineage*”

**L180 “the paraphyletic CcRPP1 clade“: by definition, a clade cannot be paraphyletic.**

RESPONSE: We thank the reviewer for this comment. We have now modified text accordingly.

**Shannon entropy

The authors use amino acid-specific estimates of Shannon entropy in alignments to identify the most variable regions within NRC subfamilies and to compare NRC subfamilies in terms of protein sequence diversity.**

RESPONSE: We fully agree with the reviewer’s comments and, to be honest, some of us think it’s totally useless analyses. However, given that it has been used in the NLR literature, we felt that we should include it here in addition to selection tests. After extensive discussion, we decided to retain the Shannon entropy analyses but have now included the concerns of the reviewers to the discussion. If anything, this study offers a direct side by side comparison of the methods.

**There are several problems with this approach: phylogenetic non-independence, differences in sample size between lineages, heterogeneity in alignment quality, and alignment composition. The authors should consider these factors, or at least justify their omission, so that readers are aware of the limitations of the analyses.**

**An approach based on synonymous or non-synonymous diversity calculations on sliding windows might be more informative than a noisy approach based on amino acid-specific estimates.

Sample size: While the definition of Shannon entropy is independent of sample size, its estimation from experimental data is highly dependent on it, with smaller samples leading to greater underestimation of the true underlying diversity (Konopiński 10.7717/peerj.9391). In the present case, there are more sites with high entropy in the largest subfamily, suggesting a bias. Using rarefaction methods could eliminate the effects of sample size differences and allow to verify the existence of this bias.**

RESPONSE: The reviewer correctly notes that smaller samples tend to underestimate entropy (Konopiński, 2020). In our study, the smallest clades — helpers (n=5) and sensor clade 2 (n=5) — are precisely those we identify as having low variability. Any correction for this bias would therefore increase the contrast with sensor clades 1 and 3. Furthermore, the CodeML analysis independently identified no positively selected sites in helpers or clade 2 but numerous sites in clades 1 and 3 (Figure 6A), confirming the entropy-based patterns. We have added a note in the revised manuscript acknowledging sample size considerations.

**Alignment composition: NRC sequences were identified from a set of NLR sequences filtered to remove redundancy. This removal of redundancy can upwardly bias the diversity estimate because some sequences are ignored, but at the same time it reduces the sample size, making the estimator less accurate.
In addition, these analyses are performed on alignments that mix varying assortments of paralogs and orthologs, which are strongly dependent on the number of species included in the alignment, making the values quite useless for comparisons between studies and organisms.**

RESPONSE: Our redundancy removal eliminated only sequences with 100% identity, primarily removing assembly duplicates rather than biologically meaningful variation. The entropy analysis was performed separately within each phylogenetically defined subclade using the same species set and methodology. Our conclusions are based on relative comparisons between subclades analysed under identical conditions, and the fact that different subclades show distinct diversification patterns argues against methodological artifacts. We acknowledge that absolute entropy values should be compared across studies with caution and have noted this in the revised text.

**Phylogenetic non-independence: The issue is the duplication structure of the gene family. Ancient duplications, recent duplications, or gene losses make genes not independent from each other. Paralogous copies arising from the same duplication share evolutionary history and thus have correlated traits (sequence, function, evolutionary rate). The authors might want to use a method that incorporates gene phylogeny (e.g., phylogenetic generalized least squares or mixed models with a phylogenetic covariance matrix) or at least acknowledge the bias.
There are other ways to estimate diversity in the genealogy of a gene family, and I encourage authors to consider them. These include: calculating the mean identity of sequences, distances between sequences within or between subfamilies, sums of branch lengths (Faith's PD)…**

RESPONSE: We acknowledge that gene duplication history introduces phylogenetic non-independence, which is an inherent feature of studying multigene family evolution. We have added a statement in the revised manuscript acknowledging this.

**Alignments: The presence of many more high-entropy sites in the largest subfamily and in the LRR domain, which contains repeats and is therefore more difficult to align, supports the view that alignment quality is a significant confounding factor. Can the authors show that the concentration of sites with high entropy in the LRR of the third subfamily is not simply a consequence of alignment errors, in addition to the higher number of sequences?**

RESPONSE: We thank the reviewer for this critical technical comment. We took steps to mitigate alignment artifacts: alignments were generated with MAFFT [--localpair] option and trimmed with ClipKIT's gappy mode to remove poorly aligned columns. Three observations argue against alignment error as the source of elevated LRR entropy in clade 3. First, when mapped onto predicted structures, high-entropy sites cluster specifically on the concave LRR face (Figure 5B). Second, the CodeML analysis, using independently generated codon alignments from MACSE, identifies positively selected sites in the same domain and structural surface (Figure 6C). Third, clade 1 shows high entropy in the CC domain but not the LRR, demonstrating that elevated entropy is not uniformly associated with larger clade size or the LRR domain per se but reflects clade-specific patterns.

**Positive selection

The authors conducted positive selection analyses independently for each gene subfamily rather than on the overall alignment. Reducing the sample size reduces the power of the analysis. This is especially significant when the number of sequences is limited, as in the helper subfamily, where only 5 sequences are available. It may also be necessary to correct for multiple tests if the same outgroups are included in different tests.**

RESPONSE: We chose to analyse each subclade independently because the biological question concerns differences in selective regime between subclades — helpers versus sensors, and between sensor clades with different helper dependencies. A single analysis on the combined alignment would test for positive selection across the entire NRC superclade but would obscure these clade-specific patterns, which are the focus of our study. We acknowledge that the helper clade (n=5) has limited power, but note that the absence of positively selected sites in helpers is consistent with all other evidence in our study: low entropy, low pairwise dN/dS, high sequence conservation, and the known functional constraints on helper NLRs across asterids. Regarding multiple testing, the outgroup sequences (AtZAR1, NbZAR1) were used for rooting the phylogenetic tree and were not included in the CodeML analyses themselves. **Another issue is again alignment quality. Properly aligning homologous coding sequences is a deceptively difficult task, but it has a major impact on the outcome of dN/dS analyses. Here again, the difficulty of aligning the LRR could have an impact and explain the large number of sites detected as being under positive selection in this domain.**

RESPONSE: Please refer to the argument for alignment quality for entropy.

**The authors compare the dn/ds across the different subfamilies but do not perform any tests; given the distributions (Figure S11), it is unlikely that the tests would be significant. Why not perform a branch-by-branch analysis on the complete phylogeny?**

RESPONSE: The reviewer raises a valid point. We did not perform formal statistical tests comparing dN/dS distributions between subclades, and we agree that given the overlapping distributions in Figure S11, such tests may lack power. In the revised manuscript, we added CodeML branch-site tests (free-ratio vs. one-ratio models) on the Lactuca NRC clade phylogenetic tree (see Figure S10).

**Copy number variation

L187 and on: This paragraph is difficult to follow and of relatively little interest because the authors do not clearly explain the hypothesis they have in mind.**

RESPONSE: We rephrased the text and provided a rational explanation for the analysis. The “copy number variation” is now rephrased.

**Reviewer #4**: **"A hierarchical immune receptor network in lettuce reveals contrasting patterns of evolution in sensor and helper NLRs" (Pai et al.).

In this study a de novo annotation of NLR in Astragales and Solanales species is generated. As I understand (but the Introduction is not very clear about it), the NRCs have been characterised in Astragales species and found to be part of large families. In this paper, their counterparts in the Solanales lineages (especially Lactuca spp) are studied, showing a lesser level of differentiation (in terms of numbers of copies). Clades in Solanales NRCs are described. Their physical clustering is investigated, revealing constrasted patterns, indicative of an ancestral clustered status followed by latter rearrangements in the different lineages. The functional role of NRC copies in triggering cell death is investigated, showing the patterns of dependency between helper and sensor NRCs. In silico protein structure is determined, showing that only helper NRCs are able to form resistosome structures. Finally, patterns of molecular evolution are investigated, indicating patterns of adaptive evolution caused by coevolution with pathogens.

I found the article to be very well written, the analyses are well conducted and the article benefics from a multi-disciplinary approach to tackle the evolution of NRCs.**

RESPONSE: We thank the reviewer for their kind words.

**My big issue is that the paper is not really focused on one (or several) scientific question. Actually no question or hypothesis seems to be formulated neither in the Abstract nor the Introduction. Because of this, readers outside the field of plant immunity might see the paper as a collection of data with less relevance to them than what the Authors certainly have in mind. Without going to details, while reading the draft I frequently asked myself why the results are important, to what question do they answer, and what was I supposed to expect in the first place.**

RESPONSE: We thank the reviewer for raising this issue. The abstract is now rephrased to better formulate our questions and hypothesis: *“However, the degree to which evolutionary patterns reflect functional divergence remains poorly understood, notably in important crop species.”*

**Some readers might find the title to be misleading because a specific class of NLRs (namely the NRCs) is being investigated.**

RESPONSE: We have modified the title based on a suggestion from the editor. There are conflicting comments on how specific the title should be. We think the rephrased title is understandable by broad audience and not misleading by referring to specific network.

**In the Abstract, the expression "helper dependency" lacks context (L.35). The Introduction is complete and instructive but the level of details might be excessive, particularly concerning the structure of the NLR family which are of relative interest for this paper. In contrast, the introduction of NRCs is rather indirect (L.73), then with more focus (L.84) but without really saying why we need to pay attention to them.**

RESPONSE: We have modified the introduction and abstract and this is now addressed.

**I don't see why Figure S2 is called on L.160, and Figure 2A on L.217.**

RESPONSE: This is now addressed and fixed.

**Text on lines 189-200 does not match the header.**

RESPONSE: We thank the reviewer for this comment. The first paragraph first explains and introduces the phylogeny of the network in Lactuca genus and then we dive deeper into the genetic clustering of the sensors and helper. We now rephrased the starting sentence for better clarity.

**Regarding the analysis of position selection, I recommend following the advice given in PAML documentation and compare the M2a model to M1a (instead of M2 vs. M1) and M8 to M8a (instead of M7), as both tests will be more conservative. What is the use model M3 in the analysis?**

RESPONSE: We appreciate the reviewer comment. We have now updated the comparisons to M2a vs M1a and M8 vs M8a. This was changed in the text as well.

**There is no need to use a predicted 3D structure to assign sites to a domain (L.391).**

RESPONSE: We appreciate the comment from the reviewer however we feel it would be insightful to structurally visualize the sites under positive selection, considering the prior studies and extensive structural work on NLR proteins.

**I am not sure it is relevant to claim that pairwise dN/dS ratios are consistent with CodeML (L.416) as this is actually the same signal.**

REPONSE: This is now fixed.

**Posterior probability of what? (L.423).**

REPONSE: We have now addressed this in the figure legend.

**The NRC network is qualified as "minimal" but is it really minimal? A minimal network would be a mere pair of NRC-S / NRC-H proteins.**

RESPONSE: This now is fixed, we avoid using the term “minimal.”.

**"A shift in helper dependency may have facilitated sensor diversification and expansion" (L.560) seems highly speculative.**

RESPONSE: We thank the reviewer for this comment, we agree that this point is speculative. However, as it is presented in the Discussion, we intend it as a hypothesis to stimulate further investigation.
