# Supplementary material for: Contrasting evolutionary patterns of helper and sensor NRC NLRs in lettuce reflect functional divergence following subfunctionalization
Source: PLoS Genet. 2026 Jul 16;22(7):e1012245. doi: 10.1371/journal.pgen.1012245 (PMC13390941; doi:10.1371/journal.pgen.1012245)
Supplement: Attachment — Submitted filename: PGENETICS_reviewer_comments_response_v4.docx [file pgen.1012245.s028.docx]

**Editor Comments:**

I was pleased to see that this manuscript has been carefully revised. The referees also found the revisions overall satisfactory, but they had additional, very valuable suggestions left. I agree that using exact terms is important, and that past errors should not be perpetuated, so please correct the use of clade and clustered. It is also important to use more appropriate analyses than Shannon entropy, to revise the positive selection analyses and to make clear that the sensor-helper relationships for the other Lactuca species have not been experimentally validated. I also find very important to clearly state the questions addressed at the end of the introduction. Other than that, I congratulate the authors for this nice piece of work that will be a great contribution to the journal.

**RESPONSE: We thank the editor for the kind words and constructive feedback. We have now carefully revised the manuscript according to the new reviewer comments and feedback. We addressed the concerns around the positive selection analysis. We have removed the Shannon Entropy analysis from this study. We have also clarified the objectives and goals of the study in the introduction. You can find the details of our revisions below.**

**Reviewers' comments:**

Reviewer's Responses to Questions

**Comments to the Authors:
Please note here if the review is uploaded as an attachment.**

Reviewer #1: In their revisions, the authors have addressed most concerns raised by the editor and reviewers. The authors have not provided any additional data that address my main concern: the sensor-helper relationships for the other Lactuca species are inferred from sequence similarity. Strong statements, such as the heading in L391-392 (“Sensors dependent on Ast-LsatNRC1 are under the higher positive selection compared to LsatNRC0-dependent sensors“) remain without experimental validation of the sensor-helper relationships. Should these assumed relationships not hold, those groups were incorrectly defined, and the consequent interpretations on differences in selection are invalid.
I understand that there are financial and other constrains that prevent the authors from including additional experiments. To strengthen the authors hypothesis, I would appreciate if the authors acknowledged correlative evidence in their favour: NRC0 is absent from L. saligna, and so are class I sensors (Figure 2a). Explicitly mentioning this fact would strengthen the claim of class 2 and 3 sensors acting through NRC1.
Notably, the absence of NRC0 in L. saligna may conflict with L32 of the abstract: “the phylogenetically conserved helper NRC0” and similar statements on the conservation of NRC0 (e.g. L497, L558), which I think should be consolidated with the findings presented in the manuscript. I was unable to find any mention regarding the absence of NRC0 from L. saligna in the manuscript main text, although there is extensive discussion of NRC0, specifically regarding the degree of conservation, but not in regard to presence / absence variation.
Absence of NRC0 and Class 1 sensors in L. saligna raises the question if the difference in species composition of the three sensor classes has any implications for the diversity & selection analysis, since there is species imbalance between classes of NLR sensors.

**Response: We thank the reviewer for their useful comments. We have indeed observed the same pattern in presence/absence of NRC0 and its dependent NRC-S but missed to mention it in the text. We have now added a paragraph expanding on this topic at the end of the relevant results section (L466-472; v5 tracked).**

„To address the reviewer’s concern, we have added disclaimers to the text (L334-337, L625-628) stating that the interactions in related species are phylogenetic inferences that serve as a predictive model requiring future experimental validation.“
I assume the authors refer to L293-296 and L586-590, which include statements that future work is required.

Minor comment:

The observation in L290 (“Interestingly, NRC-S that functioned with both helpers are clade 2 members positioned between clades 1 and 3“) is not very meaningful, as one could flip the node where clades 2 and 3 split without affecting the overall tree.

**Response: We removed this statement in the revised version.**

Reviewer #2: I judge the authors have addressed the majority of concerns raised by the reviewers.

**Response: We thank the reviewer for their constructive feedback during the previous round of reviews.**

Reviewer #3: I commend the authors for revising their article. I agree with most of the changes, but I regret the use of Shannon entropy on this dataset and in previous studies, even if the authors are satisfied with the results. It's a bit like using the dN/dS test at the intraspecific level: common in the literature despite being inappropriate.

**Response: We thank the reviewer for their suggestions. As we mentioned before we agreed with the reviewers’ points of view about this analysis. We have now decided to remove the Shannon entropy analysis altogether from the study.**

I also stand by my comment about the term "clade" because I believe (1) word meanings matter, especially in the post-truth era, and (2) there's no obligation in science to perpetuate past errors. The authors still use "clade" to refer to what is clearly a "gene family." A clade is a monophyletic group of organisms, while a gene family is a group of homologous sequences. Keeping this confusion is like mixing up the passenger (the NLR gene) with the vehicle (the Asterales/Solanales taxa). For clarity in specialized literature, it's important to use "gene family" or "orthogroup" instead of "clade" (or even worse, "superclade") to prevent a fundamental biological category error. L96: monophyletic clade is tautological.

**Response: We have now revised the term “clade” and “superclade” in the manuscript to “phylogroups”, “phylogenetic groups”, and “gene family” throughout the manuscript, whenever necessary.**

Reviewer #4: "A hierarchical immune receptor network in lettuce reveals contrasting patterns of evolution in sensor and helper NLRs" (Pai et al.), revision.

I have evaluated the first revision of the manuscript and I would like to thank the authors for taking my comments on the previous version in consideration.

Below are my comments for this revised version. Line numbers correspond to revised version of the manuscript (without edition marks). My main comments concern (1) the definition of objectives of the conducted research, (2) clarity of the text about clusters1, (3) definition of NRC sensors clades, and (4) remarks on molecular evolution analysis.

**Response: We thank the reviewer for their thorough comments and feedback. We’ve addressed all their points. You can find our responses under each point.**

1. Questions and objectives.

I made the remark that the paper was sometimes difficult to follow because research questions were not clearly expressed. This was also the cause of a comment of Reviewer #3 concerning the paragraph starting at L.202 (new version). My comment was addressed by adding the sentence, "the degree to which evolutionary patterns reflect functional divergence remains poorly understood, notably in important crop species." I still think the Introduction should list the objectives of the paper and why they are important. To be more specific, the last paragraph of the Introduction doesn't introduce why it is interesting to monitor the number of NRC copies in Asterales and to look into they genomic organization.

**Response: To further clarify the objectives of this study we reformulated the last paragraph of the introduction. The new version provides clarity on the objectives and goals of the study.**

2. Clusters.

There was some confusion around the use of the term "clustered". I agree that in most cases the meaning is understandable through context but it is better to avoid relying on context. For example, I understand tht RGCs are phylogenetic clusters and MRCs are physical clusters (L.126 and following). I find that counter-intuitive. Of course one cannot rewrite the litterature but it would clarify to write explicitly that "cluster" has completely different meanings for RGCs and MRCs. Rather subtly, the term "further" (L.128) even reinforces the idea that we speak of the same type of clusters. For me "genetically clustered" is ambiguous because, when copies are clustered in a tree, it is also connected to genetic information. So I would suggest using "physically" rather than "genetically" (or "clustered in the genome") versus "phylogenetically clustered" or, better for me, "grouped in tree" (possibly "phylogenetically grouped").

**Response: We addressed this comment by changing the “genetically” to “physically” or “genomic” whenever necessary. In other cases, its clearly stated what kind of clustering is meant in the text.**

3. Definition of NRC sensors clades.

L.211: Figure 2A is used to define three sensor clades based on ambiguous criteria. I assume that the definition of a subclade here is the smallest possible monophyletic group with a basal node with at least 70% bootstrap support, but I might assume incorrectly.

**Response: Reviewers assumption is correct. However, the grouping of NRC-H and NRC-S was also biased by our previous knowledge of phylogenetic relationships within this family. This now has been clarified in the text (L296-304).**

4. Molecular evolution.

M2a vs. M1a and M8 vs. M8a are two different tests that are essentially identical in spirit. Why perform both? I would say that the latter is more conservative and (in general) should be preferred if the amount of information available is limited, but might be too simplified if data allow to discriminate between 10 different classes of dN/dS ratios.

**Response: We have now removed M2a vs. M1a analysis and only retained the M8 and M8a comparison.**

A critical threshold of 1% has been used for PAML model comparisons while 5% is classically used. At 5%, there is also mildly convincing evidence of positive selection (with the M8 model) in the helper clade. These tests for positive selection, especially M8 vs. M8a, are known to be rather liberal and, furthermore, sensitive to alignment issues (see comments by Reviewer #3) as well as gene conversion events. Besides, the existence / location of predicted sites in the helper clade and in sensor clades 1 and 2 does not support strongly the hypothesis of position selection in these clades. Finally, the likelihood ratios for clade 3 are overwhelming (even if this may just reflect an increased power of detection). So I find it convincing to conclude that positive selection seems to be convincingly concluded for sensor clade 3 but should be considered with caution for the other clades.

**Response: We thank the reviewer for these comments. We have addressed each point as follows.**

**We have made our safeguards explicit in the Methods: a 1% LRT threshold, corroboration by BEB sites at two posterior thresholds (P > 0.95 and P > 0.99), and codon-aware alignment with MACSE. We have also removed the M1a/M2a comparison in response to the reviewer's related point, retaining M8a/M8.**

**The two-tier BEB reporting directly addresses the liberality concern. For sensor clade 3, 33 of 48 sites exceed P > 0.99 and cluster on the LRR concave face — a pattern unlikely to reflect test liberality or alignment artefacts. For the helper clade, the marginal M8 result at an uncorrected 5% threshold is not supported by any BEB site at either threshold, consistent with a false positive. We have tempered the Results and Discussion accordingly: positive selection is now presented as robustly supported only for clade 3, the clade 1 signal is described as weaker, and the helper clade as showing no support under our criteria.**

Of course, the comparison of the different clades is difficult due to the fact that they comprise different numbers of gene copies, but also they seem the have different depths (sensor clade 3 seems to be composed of more similar sequences, which also limits power but also maybe increases the risk of false positives). Possibly, the total length of the different trees (PAML reports dS total length, which is informative) can help argue that the four subtrees are not so different as it seems (the depth of other clades compensating their smaller size) but I am not sure it would set the debate. Nevertheless, in the end of the day, the evidence of positive selection in sensor clade 3 is still conclusive enough to warrant the conclusion that molecular adaptation is acting specifically in this clade.

**Response: We have added a supplementary table reporting, for each subclade, the number of sequences, total tree dS length, and mean pairwise dS from the codeml output. As the reviewer anticipated, the shallower clade 3 (now group 3) and the deeper but smaller subclades are not as different in total divergence as sequence counts suggest. We have adopted the reviewer's overall framing in the manuscript: group 3 is conclusive, the other subclades warrant caution.**

The free ratio branch model of PAML was used to estimate dN/dS for all individual branches. The amount of information available for each branch is ofter too low to make this model really useful. The mere fact branch-specific dN/dS estimates "saturate" due to a lack of dS variation is an indication that this is a somewhat unreliable metric. The objective here is again not very explicit ("to further investigate lineage-specific selection pressures"). If the aim is to demonstrate that positive selection is occurring specifically in either subclade, running branch-site model A of PAML might be appropriate. In my view it would partially alleviate the issue of different clade sizes. If the aim is to show that some sites evolve under different selective pressures according to the clade (without specifically searching for positive selection), branch-site models C and D of PAML are appropriate. Finally, if the aim is to show that the overall dN/dS ratio across the whole protein is different in the different clades, it is possible to compute an average ratio per clade using the branch model 2. The interest of these three options is that they all allow formal hypothesis testing against an appropriate null model. This does not eliminate the interest of having branch-specific dN/dS ratios but allow designing a test and precisely answers to a given question.

It seems to me that the branch model 2, which I proposed above, would be a better way to compare average dN/dS per clade, while, as I said, allowing formal testing (this relates to the paragraph 426-435).

**Response: We agree that the free-ratio model does not provide a formal test and that our stated aim was insufficiently specific. As the aim of the paragraph at L426–435 is to compare average dN/dS across subclades, we have replaced the free-ratio analysis with branch model 2 (each subclade assigned its own ω, tested against M0 by LRT). Results are reported in the Results section and supplementary table. The free-ratio analysis is retained in Figure S11 as an exploratory visualization, with saturated ω ≥ 10 branches flagged in the caption. The Methods have been updated accordingly.**

Other comments:

Title:
I agree that the title is more understandable now but now I have the feeling that it contains too much information. What I precisely mean is that the title is a sentence where the subject is separated from the verb by an information-rich clause ("in a genetic network of NLR immune receptors in lettuce"), making the title difficult to grasp fully in one go.

**Response: We have amended the title to be more simplified and concise.**

L.96 & 162 If you use the term "clade" you can't qualify it as "monophyletic" because a clade is by definition monophyletic

**Response: The terms “clade” have been revised throughout the manuscript.**

L.105 "sensor NLRs evolve rapidly in response to pathogen evolution": I would call it a possibility, but is it demonstrated at this point? Similarly, "diversification pressures that minimize cross-activation" is awkwardly formulated (do you mean divergent selection?) and I also wondered whether it is demonstrated.

**Response: Both claims are mentioned in the cited sources. For clarification we also reworded the sentences.**

L.168 "much less expanded": the formulation implies that both lineages underwent an expansion of NRC gene families. Is there evidence for that?

**Response: Given our observations of NRC gene family copy numbers across different lineages, together with comparisons to previous reports of NRC diversification in other lineages, we concluded that NRC expansion and diversification have occurred independently across lineages (Goh et al. 2024; PMID: 38922300).**

L.202: the header does not seem to describe accurately the content of the section.

**Response: The header is now changed to match the text.**

Figure 2A: I would have appreciated a highlight on the copies used in the functional analysis (i.e. those who are used in Figure 3).

**Response: We have now added the number of sequences to the figure legends.**

L.313 and 332: I tend to agree with Reviewer #1's comment on the ability of AlphaFold 3 to discriminate helper vs. sensor NRCs. For me it is merely an issue of wording. As I understand, AlphaFold is not used as a classificiation model but provide a prediction that allows to clearly assign proteins to either category. In that case it is more correct to say that AlphaFold can be used to distinguish helper vs. sensor NRCs.

**Response: We added “can be used” for clarification.**

L.347: every protein family has a "common evolutionary origin", which makes the whole sentence pointless. Do you mean a "recent" origin?

L.367-369: a sentence has been added to mention the limitations of using entropy statistics. I feel this sentence causes more harm than good because the reader may wonder whether these analyses are relevant at all. Despite these limitations, what can we do of these values? Do they confirm/complement selective pressure (i.e. dN/dS) analysis? Obviously, more detailed discussion should go in the Discussion section.

**Response: Given the reviewers’ suggestions we have now removed the Entropy analysis from the study.**

L.550-552: similarly, a sentence has been added to acknowledge limitations of the phylogenetic approach, but tends to break the development since we don't clearly understand what conclusion should be trusted before moving on to the next paragraph.

**Response: We agree the previous placement of the limitations sentence interrupted the flow before a clear conclusion was reached. We have restructured the paragraph to end on a brief synthesis of what the selection analyses support, after which the caveat about phylogenetic non-independence and uneven comparison sizes is retained as a closing qualification rather than an interruption.**

L.391: please review spelling of the header.

**Response: It is now fixed.**

L.408: don't you mean "both" comparisons?

**Response: Fixed.**

L.510-512 "Our phylogenetic analysis provides a high-confidence framework for the evolution of these clades across the Lactuca genus": sentence not clear.

**Response: It is removed the discussion.**

L.558 and following: I would not cite Figure 7 more that once per paragraph and, besides, I would introduce it explicitly as a proposed model of evolution of the NRC network.

**Response: It is now fixed and the model is clarified in the figure legend.**

L.576 "certain nodes": I agree that a member of a genetic network can be designed as a node (or possibly a vertex) but in this context I think that "genes" will appear more natural.

**Response: We agree with the reviewer. It is now corrected.**

L.698: the use of the term "significant" might cause confusion with a frequentist-type statistical test, which the BEB procedure is not.

**Response: We agree and have removed "considered significant" to avoid implying a frequentist test. The sentence now describes the two BEB posterior probability thresholds (P > 0.95 and P > 0.99) used to indicate strong support for positive selection.**

Minor typos:
L.30 "Lactuca": in italics
L.46 "are remarkably consistent that sensors": miss a few words
L.47 "the evolutionary tempo of sensors is also shaped by": maybe use "is associated with" to avoid the risk of a premature conclusion.
L.523 a comma is needed between "we demonstrated that" and "in monocot NLR pairs".
L.679 "to calculate" - typo.

**Response: All taken into account and fixed.**

L.158 I wonder whether Figure 1A should be cited in the Introduction.

**Response: We took the reviewer suggestion and added this citation in the part about evolution and emergence of the NRC network in the introduction.**

L.79 maybe it could clarify to use the terms "homologous/heterologous complex" around here.

**Response: Given that this is an introduction on functional dependency of NLR proteins using homologous/heterologous may imply structural association. Instead, we clarified this by adding “functional dependency” in the text.**

**Have all data underlying the figures and results presented in the manuscript been provided?**
Large-scale datasets should be made available via a public repository as described in the *PLOS Genetics* [data availability policy](http://track.editorialmanager.com/CL0/http:%2F%2Fjournals.plos.org%2Fplosgenetics%2Fs%2Fdata-availability/1/010f019ddf94e820-5b54fe6a-40a5-4cb4-a6db-eb1b0ef52304-000000/Db79TwafLo2QwzRZz9SNWbIeooQKTpE5817KHLc2Nyo=258), and numerical data that underlies graphs or summary statistics should be provided in spreadsheet form as supporting information.

Reviewer #1: Yes

Reviewer #2: Yes

Reviewer #3: Yes

Reviewer #4: Yes

PLOS authors have the option to publish the peer review history of their article ([what does this mean?](https://track.editorialmanager.com/CL0/https:%2F%2Fjournals.plos.org%2Fplosgenetics%2Fs%2Feditorial-and-peer-review-process%23loc-peer-review-history/1/010f019ddf94e820-5b54fe6a-40a5-4cb4-a6db-eb1b0ef52304-000000/SjOayjMznKiTKCYUXpmuw40stf5ilEP7SJ4_RTF0bTA=258)). If published, this will include your full peer review and any attached files.


If you choose “no”, your identity will remain anonymous but your review may still be made public.


**Do you want your identity to be public for this peer review?** For information about this choice, including consent withdrawal, please see our [Privacy Policy](https://track.editorialmanager.com/CL0/https:%2F%2Fwww.plos.org%2Fprivacy-policy/1/010f019ddf94e820-5b54fe6a-40a5-4cb4-a6db-eb1b0ef52304-000000/Esa1MX9e9cy3AnT2HSHfq2dtgTrzcHEf8SbUcSPXJgA=258).

Reviewer #1: **Yes:** Niklas Schandry

Reviewer #2: No

Reviewer #3: No

Reviewer #4: No

[NOTE: If reviewer comments were submitted as an attachment file, they will be attached to this email and accessible via the submission site. Please log into your account, locate the manuscript record, and check for the action link "View Attachments". If this link does not appear, there are no attachment files.]

**Figure resubmission:**

While revising your submission, we strongly recommend that you use PLOS’s NAAS tool (<https://ngplosjournals.pagemajik.ai/artanalysis>) to test your figure files. NAAS can convert your figure files to the TIFF file type and meet basic requirements (such as print size, resolution), or provide you with a report on issues that do not meet our requirements and that NAAS cannot fix.
After uploading your figures to PLOS’s NAAS tool - <https://ngplosjournals.pagemajik.ai/artanalysis>, NAAS will process the files provided and display the results in the "Uploaded Files" section of the page as the processing is complete. If the uploaded figures meet our requirements (or NAAS is able to fix the files to meet our requirements), the figure will be marked as "fixed" above. If NAAS is unable to fix the files, a red "failed" label will appear above. When NAAS has confirmed that the figure files meet our requirements, please download the file via the download option, and include these NAAS processed figure files when submitting your revised manuscript.

**Reproducibility:**

To enhance the reproducibility of your results, we recommend that authors deposit laboratory protocols in protocols.io, where a protocol can be assigned its own identifier (DOI) such that it can be cited independently in the future. Additionally, PLOS ONE offers an option to publish peer-reviewed clinical study protocols. Read more information on sharing protocols at <https://plos.org/protocols?utm_medium=editorial-email&utm_source=authorletters&utm_campaign=protocols>

*In compliance with data protection regulations, you may request that we remove your personal registration details at any time.* [*(Remove my information/details)*](https://track.editorialmanager.com/CL0/https:%2F%2Fwww.editorialmanager.com%2Fpgenetics%2Flogin.asp%3Fa=r/1/010f019ddf94e820-5b54fe6a-40a5-4cb4-a6db-eb1b0ef52304-000000/w9MmCZYmCac0jQQj3wx_v0N7LcUUWXtQHhfumqo3gHg=258)*. Please contact the publication office if you have any questions.*
