# Supplementary material for: Contrasting evolutionary patterns of helper and sensor NRC NLRs in lettuce reflect functional divergence following subfunctionalization
Source: PLoS Genet. 2026 Jul 16;22(7):e1012245. doi: 10.1371/journal.pgen.1012245 (PMC13390941; doi:10.1371/journal.pgen.1012245)
Supplement: Attachment — Submitted filename: PGENETICS_reviewer_comments_response_v5.docx [file pgen.1012245.s029.docx]

**We thank the editor for their feedback. Please find our responses below:**

Editor Comments:

This manuscript has been further improved and the referees’s comment have been taken into account. I only have a few minor suggestions left.

-In the title, it is unclear contrasting and divergence between what and what

**We updated the title to be more assertive and reflect the main findings of the study**

-L43: consistent with what?

**We’ve rephrased this sentence for more clarity.**

-L143 : unclear, do you mean « We further showed that, unlike NRC-H, NRC.. » ?

**Added more details to this sentence for better understanding.**

-L154, L194 : comma after analysis

**Fixed.**

-L157 : NRC-type

**Fixed.**

-L162 : the bracket is misplaced

**Fixed.**

-L165 : distinct from what ? specific ?

**Fixed.**

-L166 : delete phylogenetically

**Fixed.**

-Define all abbreviations in all legends

**Added abbreviations to all figure legends.**

-L201 : unclear what this means

**Rephrased for clarity.**

-L320, L363 : « under higher positive selection » : awkward wording ; stronger ?

**Fixed.**

-L331-332 : the two sentences seem contradictory. The next sentence is more clearly phrased ; An additional explanation would be better.

**We added more details and clarified this paragraph and the following on group 1 and 3 sensors.**

-L335 : the groups do not show « support » but « evidence » or signs » ; analyses show support

**Fixed.**

-L343 : differed

**Fixed.**

-keep homogeneous tenses within sentences

-L393 : it is not the lineage that expanded but the gene family within this lineage

**Fixed.**

-L397 : appear ?

**Fixed.**

-L400 and elsewhere : why network and not gene family for example ? how is it a network ?

**We have used both terms throughout but more importantly we introduce the NRC network as a gene family in the discussion and early in the manuscript. Therefore we stick to the same terminology throughout for ease of reading. For further clarity we added the “NRC gene family” at the beginning of discussion as well.**

-L404 : suggested that

**Fixed.**

-L406 : it is (no abbreviation like this in written text)

**Fixed.**

-L407 : cut the sentence in two, thie sentence is too long and complex.

**The sentence is now divided into two with more clarity.**

-L439 : The phylogenetic analysis or plural

**Fixed.**
