# Supplementary material for: Contrasting evolutionary patterns of helper and sensor NRC NLRs in lettuce reflect functional divergence following subfunctionalization
Source: PLoS Genet. 2026 Jul 16;22(7):e1012245. doi: 10.1371/journal.pgen.1012245 (PMC13390941; doi:10.1371/journal.pgen.1012245)
Supplement: Peer review history

# Peer review: Decision Letter 0

**DOI:** 10.1371/journal.pgen.1012245.r001

**Authors:** Quan Wang, Tatiana Giraud

1 Dec 2025

PGENETICS-D-25-01114

A hierarchical immune receptor network in lettuce reveals contrasting patterns of evolution in sensor and helper NLRs

PLOS Genetics

Dear Dr. Toghani,

Thank you for submitting your manuscript to PLOS Genetics. After careful consideration, we feel that it has merit but does not fully meet PLOS Genetics's publication criteria as it currently stands. Therefore, we invite you to submit a revised version of the manuscript that addresses the points raised during the review process.

Please submit your revised manuscript by Jan 30 2026 11:59PM. If you will need more time than this to complete your revisions, please reply to this message or contact the journal office at plosgenetics@plos.org. When you're ready to submit your revision, log on to https://www.editorialmanager.com/pgenetics/ and select the 'Submissions Needing Revision' folder to locate your manuscript file.

Please include the following items when submitting your revised manuscript:

* A rebuttal letter that responds to each point raised by the editor and reviewer(s). You should upload this letter as a separate file labeled 'Response to Reviewers'. This file does not need to include responses to any formatting updates and technical items listed in the 'Journal Requirements' section below.

* A marked-up copy of your manuscript that highlights changes made to the original version. You should upload this as a separate file labeled 'Revised Manuscript with Track Changes'.

* An unmarked version of your revised paper without tracked changes. You should upload this as a separate file labeled 'Manuscript'.

If you would like to make changes to your financial disclosure, competing interests statement, or data availability statement, please make these updates within the submission form at the time of resubmission. Guidelines for resubmitting your figure files are available below the reviewer comments at the end of this letter.

We look forward to receiving your revised manuscript.

Kind regards,

Tatiana Giraud

Academic Editor

PLOS Genetics

Quan Wang

Section Editor

PLOS Genetics

Aimée Dudley

Editor-in-Chief

PLOS Genetics

Anne Goriely

Editor-in-Chief

PLOS Genetics

**Additional Editor Comments:**

We have now received four referee reports on your manuscript on immune receptors (NLRs) in lettuce. All reviewers found the study valuable and original, but they also raised several concerns, particularly regarding the statements about current knowledge on NLRs, as well as the use of evolutionary terminology and analyses. The referees recommend clarifying the scientific questions framing the manuscript, performing additional experimental validation, accounting for phylogenetic non-independence in the dataset, and using more appropriate methods to assess diversity and positive selection.

I also found the study very interesting and within the scope of PLOS Genetics; however, I concur with the reviewers’ comments and add further recommendations below. I strongly encourage resubmission provided the manuscript is revised accordingly. The reviewers also offer many excellent suggestions that should be carefully addressed.

More specific comments:

-The title and abstract will be difficult to understand for most readers of PloS Genetics, try to make it clearer for a broad range of readers. For example, explain what are helper and sensor NLRs, what you mean about "functional connections" and "networks". Overall, it is very difficult for non-specialist to understand the findings, the main take-home message and the significance for the broad readership of PloS Genetics.

-L67, L103: awkward wording, this sounds Panglossian: you cannot evolve a trait because it will allow future evolution, natural selection does not foresee anything and only operate among standing variation

-L135: comma after that if you keep the one after helpers

-L137: unclear what "differentially evolving" means

-L144: unclear what "expansion" means here

-L148: from the two, not both (species cannot belong to both orders)

-L150: unclear what "network superclade" means

-L155: what statistical test allows you to say "significantly"?

-L214: "genetically clustered together" is ambiguous, I had to read several times, it usually means clustering in a phylogeny, and the whole paragraph is about clustering in genealogies right?

-Figure 2 and elsewhere: the term "phylogeny" should only be used for species relationships, not gene genealogies.

-L379: this is M&M not results

--L380-386: awkward wording, explain better what are the models and what are these tests: what are you testing here exactly?

-L388, L394, L396, L399: explain to the reader what one can infer from this

-Do not refer to figures in the discussion, they should have been explained well enough in the result section

-L508: comma after that

-L519: lack signature

-L532: what does "network clade" mean?

**Journal Requirements:**

If the reviewer comments include a recommendation to cite specific previously published works, please review and evaluate these publications to determine whether they are relevant and should be cited. There is no requirement to cite these works unless the editor has indicated otherwise.

1) We ask that a manuscript source file is provided at Revision. Please upload your manuscript file as a .doc, .docx, .rtf or .tex. If you are providing a .tex file, please upload it under the item type u2018LaTeX Source Fileu2019 and leave your .pdf version as the item type u2018Manuscriptu2019.

2) Please provide an Author Summary. This should appear in your manuscript between the Abstract (if applicable) and the Introduction, and should be 150-200 words long. The aim should be to make your findings accessible to a wide audience that includes both scientists and non-scientists. Sample summaries can be found on our website under Submission Guidelines:

https://journals.plos.org/plosgenetics/s/submission-guidelines#loc-parts-of-a-submission

3) Please upload all main figures as separate Figure files in .tif or .eps format. For more information about how to convert and format your figure files please see our guidelines:

https://journals.plos.org/plosgenetics/s/figures

4) We notice that your supplementary Figures are included in the manuscript file. Please remove them and upload them with the file type 'Supporting Information'. Please ensure that each Supporting Information file has a legend listed in the manuscript after the references list.

5) Some material included in your submission may be copyrighted. According to PLOSu2019s copyright policy, authors who use figures or other material (e.g., graphics, clipart, maps) from another author or copyright holder must demonstrate or obtain permission to publish this material under the Creative Commons Attribution 4.0 International (CC BY 4.0) License used by PLOS journals. Please closely review the details of PLOSu2019s copyright requirements here: PLOS Licenses and Copyright. If you need to request permissions from a copyright holder, you may use PLOS's Copyright Content Permission form.

Please respond directly to this email and provide any known details concerning your material's license terms and permissions required for reuse, even if you have not yet obtained copyright permissions or are unsure of your material's copyright compatibility. Once you have responded and addressed all other outstanding technical requirements, you may resubmit your manuscript within Editorial Manager.

Potential Copyright Issues:

i) Please confirm (a) that you are the photographer of 3A, and S8A, or (b) provide written permission from the photographer to publish the photo(s) under our CC BY 4.0 license.

ii) Figure 7. Please confirm whether you drew the images / clip-art within the figure panels by hand. If you did not draw the images, please provide (a) a link to the source of the images or icons and their license / terms of use; or (b) written permission from the copyright holder to publish the images or icons under our CC BY 4.0 license. Alternatively, you may replace the images with open source alternatives. See these open source resources you may use to replace images / clip-art:

- https://commons.wikimedia.org

- https://openclipart.org/.

6) Please amend your detailed Financial Disclosure statement. This is published with the article. It must therefore be completed in full sentences and contain the exact wording you wish to be published.

1) State what role the funders took in the study. If the funders had no role in your study, please state: "The funders had no role in study design, data collection and analysis, decision to publish, or preparation of the manuscript."

2) If any authors received a salary from any of your funders, please state which authors and which funders..

If you did not receive any funding for this study, please simply state: u201cThe authors received no specific funding for this work.u201d

7) Please send a completed 'Competing Interests' statement, including any COIs declared by your co-authors. If you have no competing interests to declare, please state "The authors have declared that no competing interests exist". Otherwise please declare all competing interests beginning with the statement "I have read the journal's policy and the authors of this manuscript have the following competing interests"

**Reviewers' comments:**

Reviewer's Responses to Questions

**Comments to the Authors:**

**Please note here if the review is uploaded as an attachment.**

Reviewer #1: In this manuscript, Pai and colleagues explore the CC NLR Helper and Sensor networks outside of the solanales, namely in the Asterales. The authors identify sensors, which are a sister clade of the SD type sensors, albeit lacking an SD domain, and helpers, which cluster with NRC helpers from Solanales, in asterales genomes. The authors then focus on a set of 2 helpers (containing the MADA motif and able to elicit cell death / HR as autoactive variants) and 9 sensors from lettuce and show that autoactive mutants of the helpers elicit cell death, while autoactive versions of the sensors do not. However, when co-infiltrated with wild-type helpers, some (but not all) autoactive sensors trigger a cell death response. Interestingly, there is helper-sensor specificity, and sensors from different clades are able to elicit cell death with help from one (NRC0 or NRC1) helper, or in the case of sensor clade II with both helpers.

The authors then move towards a computational analysis using alphafold3, confirming previous observations that helpers are modeled as multimeric resistosome-like structures, while sensors are not. The authors then continue with a computational analysis of the helper and sensor sequences across the Lactuca genus, and observe diversification of sensors, with the CC domain diversifying in clade I sensors, and the LRR domain diversifying in clade III; while clade II is most conserved. The author then identify a number of sites that diversify as being under positive selection.

Comments:

Overall the work is presented clearly, the rationale is well explained, the findings are discussed in the context of relevant literature. However, I feel that the second half of the manuscript (Fig 4 onwards) would benefit from some further explorations:

It is not clear why only the experimentally validated NLRs from L. sativa are investigated using alphafold3, but the whole Lactuca genus is used for further phylogenetic and evolutionary analysis.

Similarly, as I understand, the interactions between sensor clades I II and III with the two helpers are inferred from the phylogeny for the other Lactuca species. I think it would be valuable to confirm these interactions experimentally, given that the authors discuss the observed diversification in the context of the sensor-helper network. I suggest validating the interactions for all sensors & helpers used in the comparative analysis, also to confirm that the diversification does not alter sensor-helper specificities.

Minor comments:

I disagree on the statement that "[..] AlphaFold 3 can distinguish between NRC-S and NRC-H based solely on sequence and their capacity to form higher-order resistosome-like oligomers." (l. 313 ) af3 has no concept of NLRs, NRCs, or helpers and sensors. All that this suggests is that NRC-S and NRC-H sequences differ (which is also evident form the presence / absence of the MADA motif), that as consequence of these differences, the sequences are modeled into different structures, and these structures can in turn be modeled into higher-order complexes in some cases, but not others. This may be useful for researchers to distinguish between NRC-S and -H, but this is not a distinction provided by af3.

Reviewer #2: Review of the paper entitled “A hierarchical immune receptor network in lettuce reveals contrasting patterns of evolution in sensor and helper NLRs” by Pai H. et al. submitted to PLoS Genetics

This paper describes genetic diversity and evolution of a subset of NLR-type resistance proteins of Asterales plant group including lettuce. In the previous studies, the authors revealed the presence of NLR networks in Solanales consisting of the NRC-type NLRs. In this manuscript, the authors expanded NRC analysis to Asterales, which is a sister group of Solanales. Based on the genome sequences of 40 Solanales and 29 Asterales species, the authors classified ~20,000 NLR gene sequences, which revealed that representation of NRC-type NLR in Asterales NLRs is only 6.6% as compared to 50% in Solanales (Fig. 1; Fig. S1, S2). The Helper NRC (NRC-H) has two groups represented by Ast-LsatNRC1 and Lsat NRC0, whereas the Sensor NRC has three groups, Clade 1, 2 and 3 (Fig. 2A). Physical linkage of Helper and Sensor NRCs were analyzed (Fig. 2B; Fig. S4), which showed variable patterns. Function of Helper and Sensor NRCs in lettuce was tested using HR-like cell death assay using Nicotiana benthamiana transient expression assay (Fig. 3), revealing that the Helper Ast-LsaNRC1 is coupled with Clade-3 Sensors and the Helper LsaNRC0 with Clade-1 Sensors, while Clade-2 Sensors can function with both the Helpers. NLR multimer formation was predicted by Alphafold3, which showed Helper NRCs may form “resistosome” structure, whereas Sensor NRCs may not (Fig. 4). Domain-wise amino acid diversity was studied for the Helpers and Sensors, showing that Clade-3 Sensor has highly variable amino acids, particularly in the LRR domain (Fig. 5). Also, signature of positive selection was detected in Clade-3 Sensor LRR domain (Fig. 6).

NLR genes in plants are known to be highly variable due to their co-evolution with pathogen effector genes. However, how NLR genes evolve is not fully understood and the studies on NLR evolution are urgently needed. Genome sequences became available from a large number of plant species, opening an avenue to study the mode and tempo of evolution of NLRs. This timely paper addresses the diversity and evolution of NRC-type NLRs of Asterales. Phylogenetic analysis and classification of NRCs to Helper and Sensor NLRs were validated with functional analysis of HR-like cell death assay, and were further addressed by Alphafold-mediated prediction of multimer resistosome formation. The results of evolution analysis are interesting. Overall, the analyses and experiments are solid, and interpretation of data is appropriate.

Minor:

L53: which and plays – remove “and”

L83: Citation no. 39 is duplicated

L223: “chromosome 3” should be “chromosome 2”

Reviewer #3: I was pleased to review this manuscript on the evolutionary history and function of NRC (NLR Required for Cell Death) immune receptors in Asterales. The manuscript's strengths lie in the quality of the writing, the figures (though good eyesight is sometimes required), the structural predictions, and the analyses of functional connections between NRCs. The manuscript's weaknesses primarily concern the evolutionary analyses, and the description of the state of knowledge on NLR evolution. I present below several ideas that could help to refine and strengthen the analyses and conclusions.

State of knowledge about NLR evolution

L40: “NLR proteins represent one of the most diverse protein families, with different NLR clades evolving at varying rates [5,6].” None of the cited papers support the claim that NLR proteins represent one of the most diverse protein families. Both papers are based on the same Arabidopsis RenSeq data and do not compare NLR with other genes. The Progozhin et al. study does not really measure evolutionary rates, since it is based on the construction of groups of sequences that are not necessarily orthologous and on a metric (per-codon Shannon entropy) that is not directly a measure of evolutionary rate. The few, most advanced analyses of NLRs vs other genes’ diversity that I am aware of are those of Bakker et al. 2006 Plant Cell (doi: 10.1105/tpc . 106.042614) and Gladieux et al. 2024 Curr Biol (doi: 10.1016/j.cub.2024.07.061), which are not cited in the manuscript.

Clade

Futuyama & Kirkpatrick 2023, Evolution, 5th edition:

Clade: The set of species descended from a particular ancestral species.

Gene family: Two or more loci with similar nucleotide sequences that have been derived from a common ancestral sequence and that have diverged to at least some degree in their functions.

None of what is described as a clade in this manuscript is a clade in evolutionary biology terms. I can’t really blame the authors, as several previous papers on the subject used this nomenclature. However, it’s particularly puzzling that the NRC aficionados use the term “clade” to refer to a “gene family”.

L180 “the paraphyletic CcRPP1 clade“: by definition, a clade cannot be paraphyletic.

Shannon entropy

The authors use amino acid-specific estimates of Shannon entropy in alignments to identify the most variable regions within NRC subfamilies and to compare NRC subfamilies in terms of protein sequence diversity.

There are several problems with this approach: phylogenetic non-independence, differences in sample size between lineages, heterogeneity in alignment quality, and alignment composition. The authors should consider these factors, or at least justify their omission, so that readers are aware of the limitations of the analyses.

An approach based on synonymous or non-synonymous diversity calculations on sliding windows might be more informative than a noisy approach based on amino acid-specific estimates.

Sample size: While the definition of Shannon entropy is independent of sample size, its estimation from experimental data is highly dependent on it, with smaller samples leading to greater underestimation of the true underlying diversity (Konopiński 10.7717/peerj.9391). In the present case, there are more sites with high entropy in the largest subfamily, suggesting a bias. Using rarefaction methods could eliminate the effects of sample size differences and allow to verify the existence of this bias.

Alignment composition: NRC sequences were identified from a set of NLR sequences filtered to remove redundancy. This removal of redundancy can upwardly bias the diversity estimate because some sequences are ignored, but at the same time it reduces the sample size, making the estimator less accurate.

In addition, these analyses are performed on alignments that mix varying assortments of paralogs and orthologs, which are strongly dependent on the number of species included in the alignment, making the values quite useless for comparisons between studies and organisms.

Phylogenetic non-independence: The issue is the duplication structure of the gene family. Ancient duplications, recent duplications, or gene losses make genes not independent from each other. Paralogous copies arising from the same duplication share evolutionary history and thus have correlated traits (sequence, function, evolutionary rate). The authors might want to use a method that incorporates gene phylogeny (e.g., phylogenetic generalized least squares or mixed models with a phylogenetic covariance matrix) or at least acknowledge the bias.

There are other ways to estimate diversity in the genealogy of a gene family, and I encourage authors to consider them. These include: calculating the mean identity of sequences, distances between sequences within or between subfamilies, sums of branch lengths (Faith's PD)…

Alignments: The presence of many more high-entropy sites in the largest subfamily and in the LRR domain, which contains repeats and is therefore more difficult to align, supports the view that alignment quality is a significant confounding factor. Can the authors show that the concentration of sites with high entropy in the LRR of the third subfamily is not simply a consequence of alignment errors, in addition to the higher number of sequences?

Positive selection

The authors conducted positive selection analyses independently for each gene subfamily rather than on the overall alignment. Reducing the sample size reduces the power of the analysis. This is especially significant when the number of sequences is limited, as in the helper subfamily, where only 5 sequences are available. It may also be necessary to correct for multiple tests if the same outgroups are included in different tests.

Another issue is again alignment quality. Properly aligning homologous coding sequences is a deceptively difficult task, but it has a major impact on the outcome of dN/dS analyses. Here again, the difficulty of aligning the LRR could have an impact and explain the large number of sites detected as being under positive selection in this domain.

The authors compare the dn/ds across the different subfamilies but do not perform any tests; given the distributions (Figure S11), it is unlikely that the tests would be significant. Why not perform a branch-by-branch analysis on the complete phylogeny?

Copy number variation

L187 and on: This paragraph is difficult to follow and of relatively little interest because the authors do not clearly explain the hypothesis they have in mind.

Reviewer #4: "A hierarchical immune receptor network in lettuce reveals contrasting patterns of evolution in sensor and helper NLRs" (Pai et al.).

In this study a de novo annotation of NLR in Astragales and Solanales species is generated. As I understand (but the Introduction is not very clear about it), the NRCs have been characterised in Astragales species and found to be part of large families. In this paper, their counterparts in the Solanales lineages (especially Lactuca spp) are studied, showing a lesser level of differentiation (in terms of numbers of copies). Clades in Solanales NRCs are described. Their physical clustering is investigated, revealing constrasted patterns, indicative of an ancestral clustered status followed by latter rearrangements in the different lineages. The functional role of NRC copies in triggering cell death is investigated, showing the patterns of dependency between helper and sensor NRCs. In silico protein structure is determined, showing that only helper NRCs are able to form resistosome structures. Finally, patterns of molecular evolution are investigated, indicating patterns of adaptive evolution caused by coevolution with pathogens.

I found the article to be very well written, the analyses are well conducted and the article benefics from a multi-disciplinary approach to tackle the evolution of NRCs.

My big issue is that the paper is not really focused on one (or several) scientific question. Actually no question or hypothesis seems to be formulated neither in the Abstract nor the Introduction. Because of this, readers outside the field of plant immunity might see the paper as a collection of data with less relevance to them than what the Authors certainly have in mind. Without going to details, while reading the draft I frequently asked myself why the results are important, to what question do they answer, and what was I supposed to expect in the first place.

Some readers might find the title to be misleading because a specific class of NLRs (namely the NRCs) is being investigated.

In the Abstract, the expression "helper dependency" lacks context (L.35). The Introduction is complete and instructive but the level of details might be excessive, particularly concerning the structure of the NLR family which are of relative interest for this paper. In contrast, the introduction of NRCs is rather indirect (L.73), then with more focus (L.84) but without really saying why we need to pay attention to them.

I don't see why Figure S2 is called on L.160, and Figure 2A on L.217. Text on lines 189-200 does not match the header.

Regarding the analysis of position selection, I recommend following the advice given in PAML documentation and compare the M2a model to M1a (instead of M2 vs. M1) and M8 to M8a (instead of M7), as both tests will be more conservative. What is the use model M3 in the analysis?

There is no need to use a predicted 3D structure to assign sites to a domain (L.391).

I am not sure it is relevant to claim that pairwise dN/dS ratios are consistent with CodeML (L.416) as this is actually the same signal.

Posterior probability of what? (L.423).

The NRC network is qualified as "minimal" but is it really minimal? A minimal network would be a mere pair of NRC-S / NRC-H proteins.

"A shift in helper dependency may have facilitated sensor diversification and expansion" (L.560) seems highly speculative.

**********

**Have all data underlying the figures and results presented in the manuscript been provided?**

Large-scale datasets should be made available via a public repository as described in the *PLOS Genetics*  data availability policy, and numerical data that underlies graphs or summary statistics should be provided in spreadsheet form as supporting information.

Reviewer #1: Yes

Reviewer #2: Yes

Reviewer #3: Yes

Reviewer #4: Yes

**********

PLOS authors have the option to publish the peer review history of their article (what does this mean?). If published, this will include your full peer review and any attached files.

If you choose “no”, your identity will remain anonymous but your review may still be made public.

**Do you want your identity to be public for this peer review?**  For information about this choice, including consent withdrawal, please see our Privacy Policy.

Reviewer #1: **Yes:** Niklas Schandry

Reviewer #2: No

Reviewer #3: No

Reviewer #4: No

**Figure resubmission:**

While revising your submission, we strongly recommend that you use PLOS’s NAAS tool (https://ngplosjournals.pagemajik.ai/artanalysis) to test your figure files. NAAS can convert your figure files to the TIFF file type and meet basic requirements (such as print size, resolution), or provide you with a report on issues that do not meet our requirements and that NAAS cannot fix.

After uploading your figures to PLOS’s NAAS tool - https://ngplosjournals.pagemajik.ai/artanalysis, NAAS will process the files provided and display the results in the "Uploaded Files" section of the page as the processing is complete. If the uploaded figures meet our requirements (or NAAS is able to fix the files to meet our requirements), the figure will be marked as "fixed" above. If NAAS is unable to fix the files, a red "failed" label will appear above. When NAAS has confirmed that the figure files meet our requirements, please download the file via the download option, and include these NAAS processed figure files when submitting your revised manuscript.

**Reproducibility:**

To enhance the reproducibility of your results, we recommend that authors of applicable studies deposit laboratory protocols in protocols.io, where a protocol can be assigned its own identifier (DOI) such that it can be cited independently in the future. Additionally, PLOS ONE offers an option to publish peer-reviewed clinical study protocols. Read more information on sharing protocols at https://plos.org/protocols?utm_medium=editorial-email&utm_source=authorletters&utm_campaign=protocols

# Author comment: Author response to Decision Letter 1

**DOI:** 10.1371/journal.pgen.1012245.r002

31 Mar 2026

Attachment Submitted filename: PGENETICS_respond_to_reviews_v3.docx

# Peer review: Decision Letter 1

**DOI:** 10.1371/journal.pgen.1012245.r003

**Authors:** Quan Wang, Tatiana Giraud

30 Apr 2026

PGENETICS-D-25-01114R1

Contrasting evolutionary patterns in a genetic network of NLR immune receptors in lettuce reflect functional divergence

PLOS Genetics

Dear Dr. Toghani,

Thank you for submitting your manuscript to PLOS Genetics. After careful consideration, we feel that it has merit but does not fully meet PLOS Genetics's publication criteria as it currently stands. Therefore, we invite you to submit a revised version of the manuscript that addresses the points raised during the review process.

Please submit your revised manuscript within by May 30 2026 11:59PM. If you will need more time than this to complete your revisions, please reply to this message or contact the journal office at plosgenetics@plos.org. When you're ready to submit your revision, log on to https://www.editorialmanager.com/pgenetics/ and select the 'Submissions Needing Revision' folder to locate your manuscript file.

Please include the following items when submitting your revised manuscript:

* A letter that responds to each point raised by the editor and reviewer(s). You should upload this letter as a separate file labeled 'Response to Reviewers'. This file does not need to include responses to formatting updates and technical items listed in the 'Journal Requirements' section below.

* A marked-up copy of your manuscript that highlights changes made to the original version. You should upload this as a separate file labeled 'Revised Manuscript with Track Changes'.

* An unmarked version of your revised paper without tracked changes. You should upload this as a separate file labeled 'Manuscript'.

If you would like to make changes to your financial disclosure, competing interests statement, or data availability statement, please make these updates within the submission form at the time of resubmission. Guidelines for resubmitting your figure files are available below the reviewer comments at the end of this letter.

We look forward to receiving your revised manuscript.

Kind regards,

Tatiana Giraud

Academic Editor

PLOS Genetics

Quan Wang

Section Editor

PLOS Genetics

Aimée Dudley

Editor-in-Chief

PLOS Genetics

Anne Goriely

Editor-in-Chief

PLOS Genetics

**Additional Editor Comments:**

I was pleased to see that this manuscript has been carefully revised. The referees also found the revisions overall satisfactory, but they had additional, very valuable suggestions left. I agree that using exact terms is important, and that past errors should not be perpetuated, so please correct the use of clade and clustered. It is also important to use more appropriate analyses than Shannon entropy, to revise the positive selection analyses and to make clear that the sensor-helper relationships for the other Lactuca species have not been experimentally validated. I also find very important to clearly state the questions addressed at the end of the introduction. Other than that, I congratulate the authors for this nice piece of work that will be a great contribution to the journal.

**Reviewers' comments:**

Reviewer's Responses to Questions

**Comments to the Authors:**

**Please note here if the review is uploaded as an attachment.**

Reviewer #1: In their revisions, the authors have addressed most concerns raised by the editor and reviewers. The authors have not provided any additional data that address my main concern: the sensor-helper relationships for the other Lactuca species are inferred from sequence similarity. Strong statements, such as the heading in L391-392 (“Sensors dependent on Ast-LsatNRC1 are under the higher positive selection compared to LsatNRC0-dependent sensors“) remain without experimental validation of the sensor-helper relationships. Should these assumed relationships not hold, those groups were incorrectly defined, and the consequent interpretations on differences in selection are invalid.

I understand that there are financial and other constrains that prevent the authors from including additional experiments. To strengthen the authors hypothesis, I would appreciate if the authors acknowledged correlative evidence in their favour: NRC0 is absent from L. saligna, and so are class I sensors (Figure 2a). Explicitly mentioning this fact would strengthen the claim of class 2 and 3 sensors acting through NRC1.

Notably, the absence of NRC0 in L. saligna may conflict with L32 of the abstract: “the phylogenetically conserved helper NRC0” and similar statements on the conservation of NRC0 (e.g. L497, L558), which I think should be consolidated with the findings presented in the manuscript. I was unable to find any mention regarding the absence of NRC0 from L. saligna in the manuscript main text, although there is extensive discussion of NRC0, specifically regarding the degree of conservation, but not in regard to presence / absence variation.

Absence of NRC0 and Class 1 sensors in L. saligna raises the question if the difference in species composition of the three sensor classes has any implications for the diversity & selection analysis, since there is species imbalance between classes of NLR sensors.

“To address the reviewer’s concern, we have added disclaimers to the text (L334-337, L625-628) stating that the interactions in related species are phylogenetic inferences that serve as a predictive model requiring future experimental validation.“

I assume the authors refer to L293-296 and L586-590, which include statements that future work is required.

Minor comment:

The observation in L290 (“Interestingly, NRC-S that functioned with both helpers are clade 2 members positioned between clades 1 and 3“) is not very meaningful, as one could flip the node where clades 2 and 3 split without affecting the overall tree.

Reviewer #2: I judge the authors have addressed the majority of concerns raised by the reviewers.

Reviewer #3: I commend the authors for revising their article. I agree with most of the changes, but I regret the use of Shannon entropy on this dataset and in previous studies, even if the authors are satisfied with the results. It's a bit like using the dN/dS test at the intraspecific level: common in the literature despite being inappropriate. I also stand by my comment about the term "clade" because I believe (1) word meanings matter, especially in the post-truth era, and (2) there's no obligation in science to perpetuate past errors. The authors still use "clade" to refer to what is clearly a "gene family." A clade is a monophyletic group of organisms, while a gene family is a group of homologous sequences. Keeping this confusion is like mixing up the passenger (the NLR gene) with the vehicle (the Asterales/Solanales taxa). For clarity in specialized literature, it's important to use "gene family" or "orthogroup" instead of "clade" (or even worse, "superclade") to prevent a fundamental biological category error. L96: monophyletic clade is tautological.

Reviewer #4: "A hierarchical immune receptor network in lettuce reveals contrasting patterns of evolution in sensor and helper NLRs" (Pai et al.), revision.

I have evaluated the first revision of the manuscript and I would like to thank the authors for taking my comments on the previous version in consideration.

Below are my comments for this revised version. Line numbers correspond to revised version of the manuscript (without edition marks). My main comments concern (1) the definition of objectives of the conducted research, (2) clarity of the text about clusters1, (3) definition of NRC sensors clades, and (4) remarks on molecular evolution analysis.

1. Questions and objectives.

I made the remark that the paper was sometimes difficult to follow because research questions were not clearly expressed. This was also the cause of a comment of Reviewer #3 concerning the paragraph starting at L.202 (new version). My comment was addressed by adding the sentence, "the degree to which evolutionary patterns reflect functional divergence remains poorly understood, notably in important crop species." I still think the Introduction should list the objectives of the paper and why they are important. To be more specific, the last paragraph of the Introduction doesn't introduce why it is interesting to monitor the number of NRC copies in Asterales and to look into they genomic organization.

2. Clusters.

There was some confusion around the use of the term "clustered". I agree that in most cases the meaning is understandable through context but it is better to avoid relying on context. For example, I understand tht RGCs are phylogenetic clusters and MRCs are physical clusters (L.126 and following). I find that counter-intuitive. Of course one cannot rewrite the litterature but it would clarify to write explicitly that "cluster" has completely different meanings for RGCs and MRCs. Rather subtly, the term "further" (L.128) even reinforces the idea that we speak of the same type of clusters. For me "genetically clustered" is ambiguous because, when copies are clustered in a tree, it is also connected to genetic information. So I would suggest using "physically" rather than "genetically" (or "clustered in the genome") versus "phylogenetically clustered" or, better for me, "grouped in tree" (possibly "phylogenetically grouped").

3. Definition of NRC sensors clades.

L.211: Figure 2A is used to define three sensor clades based on ambiguous criteria. I assume that the definition of a subclade here is the smallest possible monophyletic group with a basal node with at least 70% bootstrap support, but I might assume incorrectly.

4. Molecular evolution.

M2a vs. M1a and M8 vs. M8a are two different tests that are essentially identical in spirit. Why perform both? I would say that the latter is more conservative and (in general) should be preferred if the amount of information available is limited, but might be too simplified if data allow to discriminate between 10 different classes of dN/dS ratios.

A critical threshold of 1% has been used for PAML model comparisons while 5% is classically used. At 5%, there is also mildly convincing evidence of positive selection (with the M8 model) in the helper clade. These tests for positive selection, especially M8 vs. M8a, are known to be rather liberal and, furthermore, sensitive to alignment issues (see comments by Reviewer #3) as well as gene conversion events. Besides, the existence / location of predicted sites in the helper clade and in sensor clades 1 and 2 does not support strongly the hypothesis of position selection in these clades. Finally, the likelihood ratios for clade 3 are overwhelming (even if this may just reflect an increased power of detection). So I find it convincing to conclude that positive selection seems to be convincingly concluded for sensor clade 3 but should be considered with caution for the other clades.

Of course, the comparison of the different clades is difficult due to the fact that they comprise different numbers of gene copies, but also they seem the have different depths (sensor clade 3 seems to be composed of more similar sequences, which also limits power but also maybe increases the risk of false positives). Possibly, the total length of the different trees (PAML reports dS total length, which is informative) can help argue that the four subtrees are not so different as it seems (the depth of other clades compensating their smaller size) but I am not sure it would set the debate. Nevertheless, in the end of the day, the evidence of positive selection in sensor clade 3 is still conclusive enough to warrant the conclusion that molecular adaptation is acting specifically in this clade.

The free ratio branch model of PAML was used to estimate dN/dS for all individual branches. The amount of information available for each branch is ofter too low to make this model really useful. The mere fact branch-specific dN/dS estimates "saturate" due to a lack of dS variation is an indication that this is a somewhat unreliable metric. The objective here is again not very explicit ("to further investigate lineage-specific selection pressures"). If the aim is to demonstrate that positive selection is occurring specifically in either subclade, running branch-site model A of PAML might be appropriate. In my view it would partially alleviate the issue of different clade sizes. If the aim is to show that some sites evolve under different selective pressures according to the clade (without specifically searching for positive selection), branch-site models C and D of PAML are appropriate. Finally, if the aim is to show that the overall dN/dS ratio across the whole protein is different in the different clades, it is possible to compute an average ratio per clade using the branch model 2. The interest of these three options is that they all allow formal hypothesis testing against an appropriate null model. This does not eliminate the interest of having branch-specific dN/dS ratios but allow designing a test and precisely answers to a given question.

It seems to me that the branch model 2, which I proposed above, would be a better way to compare average dN/dS per clade, while, as I said, allowing formal testing (this relates to the paragraph 426-435).

Other comments:

Title:

I agree that the title is more understandable now but now I have the feeling that it contains too much information. What I precisely mean is that the title is a sentence where the subject is separated from the verb by an information-rich clause ("in a genetic network of NLR immune receptors in lettuce"), making the title difficult to grasp fully in one go.

L.96 & 162 If you use the term "clade" you can't qualify it as "monophyletic" because a clade is by definition monophyletic

L.105 "sensor NLRs evolve rapidly in response to pathogen evolution": I would call it a possibility, but is it demonstrated at this point? Similarly, "diversification pressures that minimize cross-activation" is awkwardly formulated (do you mean divergent selection?) and I also wondered whether it is demonstrated.

L.168 "much less expanded": the formulation implies that both lineages underwent an expansion of NRC gene families. Is there evidence for that?

L.202: the header does not seem to describe accurately the content of the section.

Figure 2A: I would have appreciated a highlight on the copies used in the functional analysis (i.e. those who are used in Figure 3).

L.313 and 332: I tend to agree with Reviewer #1's comment on the ability of AlphaFold 3 to discriminate helper vs. sensor NRCs. For me it is merely an issue of wording. As I understand, AlphaFold is not used as a classificiation model but provide a prediction that allows to clearly assign proteins to either category. In that case it is more correct to say that AlphaFold can be used to distinguish helper vs. sensor NRCs.

L.347: every protein family has a "common evolutionary origin", which makes the whole sentence pointless. Do you mean a "recent" origin?

L.367-369: a sentence has been added to mention the limitations of using entropy statistics. I feel this sentence causes more harm than good because the reader may wonder whether these analyses are relevant at all. Despite these limitations, what can we do of these values? Do they confirm/complement selective pressure (i.e. dN/dS) analysis? Obviously, more detailed discussion should go in the Discussion section.

L.550-552: similarly, a sentence has been added to acknowledge limitations of the phylogenetic approach, but tends to break the development since we don't clearly understand what conclusion should be trusted before moving on to the next paragraph.

L.391: please review spelling of the header.

L.408: don't you mean "both" comparisons?

L.510-512 "Our phylogenetic analysis provides a high-confidence framework for the evolution of these clades across the Lactuca genus": sentence not clear.

L.558 and following: I would not cite Figure 7 more that once per paragraph and, besides, I would introduce it explicitly as a proposed model of evolution of the NRC network.

L.576 "certain nodes": I agree that a member of a genetic network can be designed as a node (or possibly a vertex) but in this context I think that "genes" will appear more natural.

L.698: the use of the term "significant" might cause confusion with a frequentist-type statistical test, which the BEB procedure is not.

Minor typos:

L.30 "Lactuca": in italics

L.46 "are remarkably consistent that sensors": miss a few words

L.47 "the evolutionary tempo of sensors is also shaped by": maybe use "is associated with" to avoid the risk of a premature conclusion.

L.79 maybe it could clarify to use the terms "homologous/heterologous complex" around here.

L.158 I wonder whether Figure 1A should be cited in the Introduction.

L.523 a comma is needed between "we demonstrated that" and "in monocot NLR pairs".

L.679 "to calculate" - typo.

**********

**Have all data underlying the figures and results presented in the manuscript been provided?**

Large-scale datasets should be made available via a public repository as described in the *PLOS Genetics*  data availability policy, and numerical data that underlies graphs or summary statistics should be provided in spreadsheet form as supporting information.

Reviewer #1: Yes

Reviewer #2: Yes

Reviewer #3: Yes

Reviewer #4: Yes

**********

PLOS authors have the option to publish the peer review history of their article (what does this mean?). If published, this will include your full peer review and any attached files.

If you choose “no”, your identity will remain anonymous but your review may still be made public.

**Do you want your identity to be public for this peer review?**  For information about this choice, including consent withdrawal, please see our Privacy Policy.

Reviewer #1: **Yes:** Niklas Schandry

Reviewer #2: No

Reviewer #3: No

Reviewer #4: No

[NOTE: If reviewer comments were submitted as an attachment file, they will be attached to this email and accessible via the submission site. Please log into your account, locate the manuscript record, and check for the action link "View Attachments". If this link does not appear, there are no attachment files.]

**Figure resubmission:**

-->While revising your submission, we strongly recommend that you use PLOS’s NAAS tool (https://ngplosjournals.pagemajik.ai/artanalysis) to test your figure files. NAAS can convert your figure files to the TIFF file type and meet basic requirements (such as print size, resolution), or provide you with a report on issues that do not meet our requirements and that NAAS cannot fix.

After uploading your figures to PLOS’s NAAS tool - https://ngplosjournals.pagemajik.ai/artanalysis, NAAS will process the files provided and display the results in the "Uploaded Files" section of the page as the processing is complete. If the uploaded figures meet our requirements (or NAAS is able to fix the files to meet our requirements), the figure will be marked as "fixed" above. If NAAS is unable to fix the files, a red "failed" label will appear above. When NAAS has confirmed that the figure files meet our requirements, please download the file via the download option, and include these NAAS processed figure files when submitting your revised manuscript.

**Reproducibility:**

To enhance the reproducibility of your results, we recommend that authors deposit laboratory protocols in protocols.io, where a protocol can be assigned its own identifier (DOI) such that it can be cited independently in the future. Additionally, PLOS ONE offers an option to publish peer-reviewed clinical study protocols. Read more information on sharing protocols at https://plos.org/protocols?utm_medium=editorial-email&utm_source=authorletters&utm_campaign=protocols

# Author comment: Author response to Decision Letter 2

**DOI:** 10.1371/journal.pgen.1012245.r004

1 Jul 2026

Attachment Submitted filename: PGENETICS_reviewer_comments_response_v4.docx

# Editor report: Decision Letter 2

**DOI:** 10.1371/journal.pgen.1012245.r005

**Authors:** Quan Wang, Tatiana Giraud

3 Jul 2026

PGENETICS-D-25-01114R2

Contrasting evolutionary patterns in lettuce NRC NLR immune network reflect functional divergence

PLOS Genetics

Dear Dr. Toghani,

Thank you for submitting your manuscript to PLOS Genetics. After careful consideration, we feel that it has merit but does not fully meet PLOS Genetics's publication criteria as it currently stands. Therefore, we invite you to submit a revised version of the manuscript that addresses the points raised during the review process.

Please submit your revised manuscript within by Aug 02 2026 11:59PM. If you will need more time than this to complete your revisions, please reply to this message or contact the journal office at plosgenetics@plos.org. When you're ready to submit your revision, log on to https://www.editorialmanager.com/pgenetics/ and select the 'Submissions Needing Revision' folder to locate your manuscript file.

Please include the following items when submitting your revised manuscript:

* A letter that responds to each point raised by the editor and reviewer(s). You should upload this letter as a separate file labeled 'Response to Reviewers'. This file does not need to include responses to formatting updates and technical items listed in the 'Journal Requirements' section below.

* A marked-up copy of your manuscript that highlights changes made to the original version. You should upload this as a separate file labeled 'Revised Manuscript with Track Changes'.

* An unmarked version of your revised paper without tracked changes. You should upload this as a separate file labeled 'Manuscript'.

If you would like to make changes to your financial disclosure, competing interests statement, or data availability statement, please make these updates within the submission form at the time of resubmission. Guidelines for resubmitting your figure files are available below the reviewer comments at the end of this letter.

As the corresponding author, your ORCID iD is verified in the submission system and will appear in the published article. PLOS supports the use of ORCID, and we encourage all coauthors to register for an ORCID iD and use it as well. Please encourage your coauthors to verify their ORCID iD within the submission system before final acceptance, as unverified ORCID iDs will not appear in the published article. Only the individual author can complete the verification step; PLOS staff cannot verify ORCID iDs on behalf of authors.

We look forward to receiving your revised manuscript.

Kind regards,

Tatiana Giraud

Academic Editor

PLOS Genetics

Quan Wang

Section Editor

PLOS Genetics

Aimée Dudley

Editor-in-Chief

PLOS Genetics

Anne Goriely

Editor-in-Chief

PLOS Genetics

**Additional Editor Comments (if provided):**

This manuscript has been further improved and the referees’s comment have been taken into account. I only have a few minor suggestions left.

-In the title, it is unclear contrasting and divergence between what and what

-L43: consistent with what?

-L143 : unclear, do you mean « We further showed that, unlike NRC-H, NRC.. » ?

-L154, L194 : comma after analysis

-L157 : NRC-type

-L162 : the bracket is misplaced

-L165 : distinct from what ? specific ?

-L166 : delete phylogenetically

-Define all abbreviations in all legends

-L201 : unclear what this means

-L320, L363 : « under higher positive selection » : awkward wording ; stronger ?

-L331-332 : the two sentences seem contradictory. The next sentence is more clearly phrased ; An additional explanation would be better.

-L335 : the groups do not show « support » but « evidence » or signs » ; analyses show support

-L343 : differed

-keep homogeneous tenses within sentences

-L393 : it is not the lineage that expanded but the gene family within this lineage

-L397 : appear ?

-L400 and elsewhere : why network and not gene family for example ? how is it a network ?

-L404 : suggested that

-L406 : it is (no abbreviation like this in written text)

-L407 : cut the sentence in two, thie sentence is too long and complex.

-L439 : The phylogenetic analysis or plural

**Reviewers' comments:**

[NOTE: If reviewer comments were submitted as an attachment file, they will be attached to this email and accessible via the submission site. Please log into your account, locate the manuscript record, and check for the action link "View Attachments". If this link does not appear, there are no attachment files.]

**Figure resubmission:**

-->While revising your submission, we strongly recommend that you use PLOS’s NAAS tool (https://ngplosjournals.pagemajik.ai/artanalysis) to test your figure files. NAAS can convert your figure files to the TIFF file type and meet basic requirements (such as print size, resolution), or provide you with a report on issues that do not meet our requirements and that NAAS cannot fix.

After uploading your figures to PLOS’s NAAS tool - https://ngplosjournals.pagemajik.ai/artanalysis, NAAS will process the files provided and display the results in the "Uploaded Files" section of the page as the processing is complete. If the uploaded figures meet our requirements (or NAAS is able to fix the files to meet our requirements), the figure will be marked as "fixed" above. If NAAS is unable to fix the files, a red "failed" label will appear above. When NAAS has confirmed that the figure files meet our requirements, please download the file via the download option, and include these NAAS processed figure files when submitting your revised manuscript.

**Reproducibility:**

To enhance the reproducibility of your results, we recommend that authors deposit laboratory protocols in protocols.io, where a protocol can be assigned its own identifier (DOI) such that it can be cited independently in the future. Additionally, PLOS ONE offers an option to publish peer-reviewed clinical study protocols. Read more information on sharing protocols at https://plos.org/protocols?utm_medium=editorial-email&utm_source=authorletters&utm_campaign=protocols

# Author comment: Author response to Decision Letter 3

**DOI:** 10.1371/journal.pgen.1012245.r006

6 Jul 2026

Attachment Submitted filename: PGENETICS_reviewer_comments_response_v5.docx

# Editor report: Decision Letter 3

**DOI:** 10.1371/journal.pgen.1012245.r007

**Authors:** Quan Wang, Tatiana Giraud

7 Jul 2026

Dear Dr Toghani,

We are pleased to inform you that your manuscript entitled "Contrasting evolutionary patterns of helper and sensor NRC NLRs in lettuce reflect functional divergence following subfunctionalization" has been editorially accepted for publication in PLOS Genetics. Congratulations!

Before your submission can be formally accepted and sent to production you will need to complete our formatting changes, which you will receive in a follow up email. Please be aware that it may take several days for you to receive this email; during this time no action is required by you. Please note: the accept date on your published article will reflect the date of this provisional acceptance, but your manuscript will not be scheduled for publication until the required changes have been made.

Once your paper is formally accepted, an uncorrected proof of your manuscript will be published online ahead of the final version, unless you’ve already opted out via the online submission form. If, for any reason, you do not want an earlier version of your manuscript published online or are unsure if you have already indicated as such, please let the journal staff know immediately at plosgenetics@plos.org.

In the meantime, please log into Editorial Manager at https://www.editorialmanager.com/pgenetics/, click the "Update My Information" link at the top of the page, and update your user information to ensure an efficient production and billing process. Note that PLOS requires an ORCID iD for all corresponding authors. Therefore, please ensure that you have an ORCID iD and that it is validated in Editorial Manager. To do this, go to ‘Update my Information’ (in the upper left-hand corner of the main menu), and click on the Fetch/Validate link next to the ORCID field.  This will take you to the ORCID site and allow you to create a new iD or authenticate a pre-existing iD in Editorial Manager.

If you have a press-related query, or would like to know about making your underlying data available (as you will be aware, this is required for publication), please see the end of this email. If your institution or institutions have a press office, please notify them about your upcoming article at this point, to enable them to help maximise its impact. Inform journal staff as soon as possible if you are preparing a press release for your article and need a publication date.

Thank you again for supporting open-access publishing; we are looking forward to publishing your work in PLOS Genetics!

Yours sincerely,

Tatiana Giraud

Academic Editor

PLOS Genetics

Quan Wang

Section Editor

PLOS Genetics

Aimée Dudley

Editor-in-Chief

PLOS Genetics

Anne Goriely

Editor-in-Chief

PLOS Genetics

www.plosgenetics.org

BlueSky: @plos.bsky.social

----------------------------------------------------

Comments from the reviewers (if applicable):

----------------------------------------------------

**Data Deposition**

If you have submitted a Research Article or Front Matter that has associated data that are not suitable for deposition in a subject-specific public repository (such as GenBank or ArrayExpress), one way to make that data available is to deposit it in the Dryad Digital Repository. As you may recall, we ask all authors to agree to make data available; this is one way to achieve that. A full list of recommended repositories can be found on our website.

The following link will take you to the Dryad record for your article, so you won't have to re‐enter its bibliographic information, and can upload your files directly:

http://datadryad.org/submit?journalID=pgenetics&manu=PGENETICS-D-25-01114R3

More information about depositing data in Dryad is available at http://www.datadryad.org/depositing. If you experience any difficulties in submitting your data, please contact help@datadryad.org for support.

Additionally, please be aware that our data availability policy requires that all numerical data underlying display items are included with the submission, and you will need to provide this before we can formally accept your manuscript, if not already present.

----------------------------------------------------

**Press Queries**

If you or your institution will be preparing press materials for this manuscript, or if you need to know your paper's publication date for media purposes, please inform the journal staff as soon as possible so that your submission can be scheduled accordingly. Your manuscript will remain under a strict press embargo until the publication date and time. This means an early version of your manuscript will not be published ahead of your final version. PLOS Genetics may also choose to issue a press release for your article. If there's anything the journal should know or you'd like more information, please get in touch via plosgenetics@plos.org.

# Editor report: Acceptance letter

**DOI:** 10.1371/journal.pgen.1012245.r008

**Authors:** Quan Wang, Tatiana Giraud

PGENETICS-D-25-01114R3

Contrasting evolutionary patterns of helper and sensor NRC NLRs in lettuce reflect functional divergence following subfunctionalization

Dear Dr Toghani,

We are pleased to inform you that your manuscript entitled "Contrasting evolutionary patterns of helper and sensor NRC NLRs in lettuce reflect functional divergence following subfunctionalization" has been formally accepted for publication in PLOS Genetics! Your manuscript is now with our production department and you will be notified of the publication date in due course.

The corresponding author will soon be receiving a typeset proof for review, to ensure errors have not been introduced during production. Please review the PDF proof of your manuscript carefully, as this is the last chance to correct any errors. Please note that major changes, or those which affect the scientific understanding of the work, will likely cause delays to the publication date of your manuscript.

Soon after your final files are uploaded, unless you have opted out or your manuscript is a front-matter piece, the early version of your manuscript will be published online. The date of the early version will be your article's publication date. The final article will be published to the same URL, and all versions of the paper will be accessible to readers.

For Research Articles, you will receive an invoice from PLOS for your publication fee after your manuscript has reached the completed accept phase. If you receive an email requesting payment before acceptance or for any other service, this may be a phishing scheme. Learn how to identify phishing emails and protect your accounts at https://explore.plos.org/phishing.

Thank you again for supporting PLOS Genetics and open-access publishing. We are looking forward to publishing your work!

With kind regards,

Janani Seenivasan

PLOS Genetics

On behalf of:

The PLOS Genetics Team

Carlyle House, Carlyle Road, Cambridge CB4 3DN | United Kingdom

plosgenetics@plos.org | +44 (0) 1223-442823

plosgenetics.org | Twitter: @PLOSGenetics
